# Genomic and transcriptomic studies on flavonoid biosynthesis in *Lagerstroemia indica*

**DOI:** 10.1186/s12870-024-04776-4

**Published:** 2024-03-05

**Authors:** Chunmei Yu, Guoyuan Liu, Jin Qin, Xi Wan, Anfang Guo, Hui Wei, Yanhong Chen, Bolin Lian, Fei Zhong, Jian Zhang

**Affiliations:** 1 School of Life Science, Nantong University, No. 9 Seyuan Road, Nantong, Jiangsu Province 226019 China; 2 Key Lab of Landscape Plant Genetics and Breeding of Nantong, No. 9 Seyuan Road, Nantong, Jiangsu Province 226019 China

**Keywords:** *Lagerstroemia indica*, Whole genome triplication, Anthocyanin biosynthesis, MYB transcriptional factor, LiTTG1

## Abstract

**Background:**

*Lagerstroemia indica* is a widely cultivated ornamental woody shrub/tree of the family *Lythraceae* that is used as a traditional medicinal plant in East Asia and Egypt. However, unlike other ornamental woody plants, its genome is not well-investigated, which hindered the discovery of the key genes that regulate important traits and the synthesis of bioactive compounds.

**Results:**

In this study, the genomic sequences of *L. indica* were determined using several next-generation sequencing technologies. Altogether, 324.01 Mb sequences were assembled and 98.21% (318.21 Mb) of them were placed in 24 pseudo-chromosomes. The heterozygosity, repeated sequences, and GC residues occupied 1.65%, 29.17%, and 38.64% of the genome, respectively. In addition, 28,811 protein-coding gene models, 327 miRNAs, 552 tRNAs, 214 rRNAs, and 607 snRNAs were identified. The intra- and interspecies synteny and Ks analysis revealed that *L. indica* exhibits a hexaploidy. The co-expression profiles of the genes involved in the phenylpropanoid (PA) and flavonoid/anthocyanin (ABGs) pathways with the R2R3 MYB genes (137 members) showed that ten R2R3 MYB genes positively regulate flavonoid/anthocyanin biosynthesis. The colors of flowers with white, purple (PB), and deep purplish pink (DPB) petals were found to be determined by the levels of delphinidin-based (Dp) derivatives. However, the substrate specificities of LiDFR and LiOMT probably resulted in the different compositions of flavonoid/anthocyanin. In *L. indica*, two *LiTTG1s* (*LiTTG1-1* and *LiTTG1-2*) were found to be the homologs of *AtTTG1* (*WD40*). LiTTG1-1 was found to repress anthocyanin biosynthesis using the tobacco transient transfection assay.

**Conclusions:**

This study showed that the ancestor *L. indica* experienced genome triplication approximately 38.5 million years ago and that *LiTTG1-1* represses anthocyanin biosynthesis. Furthermore, several genes such as *LiDFR*, *LiOMTs*, and R2R3 *LiMYB*s are related to anthocyanin biosynthesis. Further studies are required to clarify the mechanisms and alleles responsible for flower color development.

**Supplementary Information:**

The online version contains supplementary material available at 10.1186/s12870-024-04776-4.

## Background

**Fig. 1:**
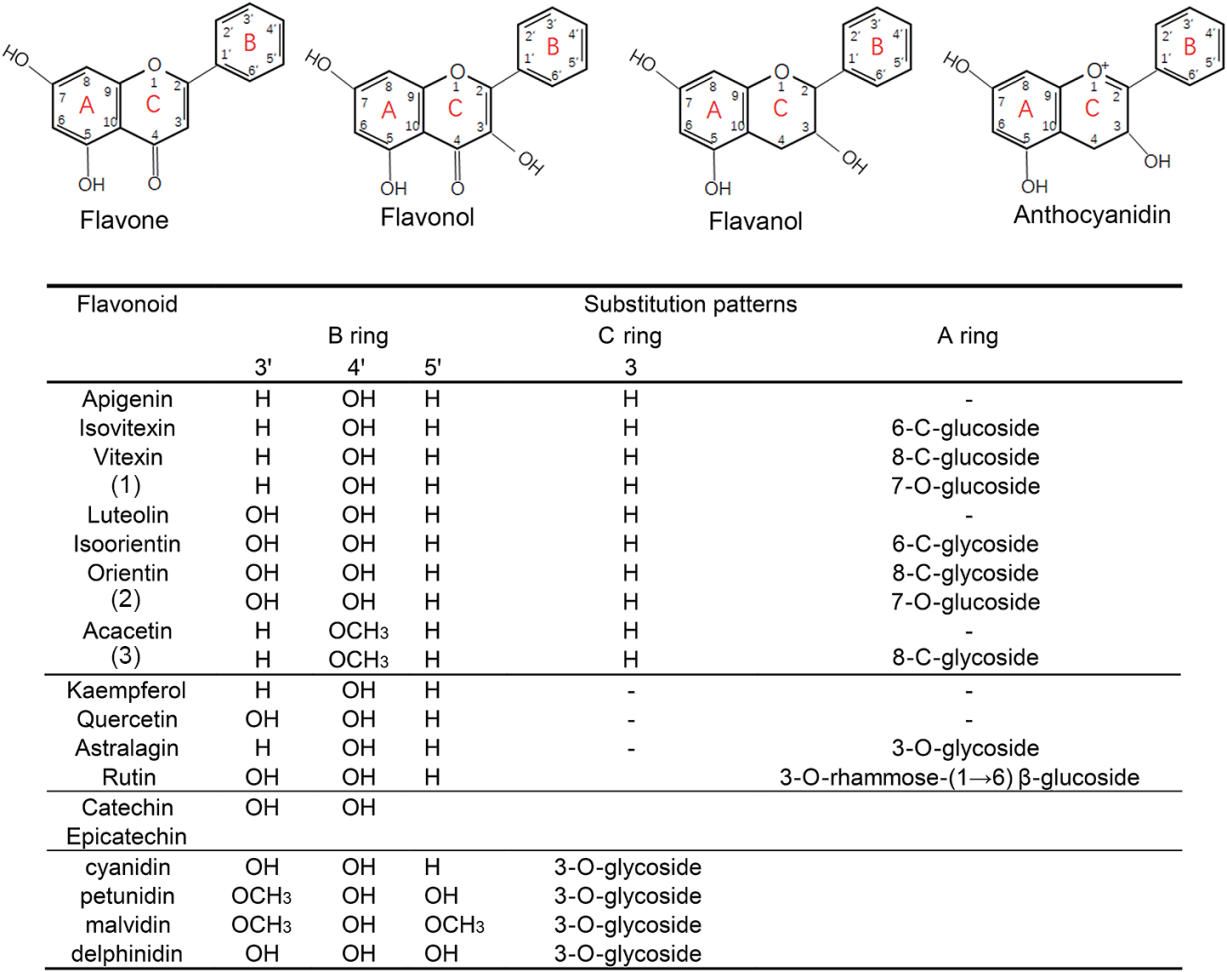
Four classes of flavonoids in crape myrtle. Compounds (1), (2), and (3) are derivatives of vitexin, luteolin, and acacetin, respectively

*Lagerstroemia indica* (crape myrtle) belongs to *Lythraceae* in *Myrtale* [1]. It is used as an ornamental plant due to its long flowering period, flowers of different colors, and smooth trunk. To create new varieties with special traits, such as purple leaves, deep red flowers, dwarf, and weep architecture, breeders have made great efforts to identify genes associated with these traits [2–6]. Moreover, it is used as a traditional medical plant in Asia and Egypt due to its analgesic, antipyretic, antihyperglycemic, antioxidant, hepatoprotective, and antimicrobial activities. Phenolic compounds, such as gallic acid and its derivatives, and different classes of flavonoids have been identified as the active constituents of the plant (Fig. 1) [7].

The precursor of all flavonoids is phenylalanine, which is catalyzed to 4-coumaroyl-CoA (*p*-coumaroyl-CoA) by phenylalanine ammonialyase (PAL, EC: 4.3.1.5), cinnamate 4-hydroxylase (C4H, EC: 1.14.13.11), and 4-coumarate CoA ligase (4CL, EC: 6.2.1.12). The genes encoding these three enzymes are called the phenylpropanoid (PA) pathway genes. 4-coumaroyl-CoA (p-coumaroyl-CoA) is converted to either a flavonoid, lignin, lignan, coumarin, or stilbenoid, depending on the downstream enzymes [8]. Chalcone synthase (CHS) and chalcone isomerase (CHI) convert p-coumaroyl-CoA to form the flavonoid skeleton, which is then converted into any of the different types of flavonoids. For example, it can be converted to flavone by flavone synthase (FNS); flavonol by flavanone 3-hydroxylase (F3H), flavonoid 3ʹ-hydroxylase (F3ʹH), and flavonol synthase (FLS); or anthocyanin by F3H, F3ʹH or flavonoid 3ʹ5ʹ-hydroxylase (F3ʹ5ʹH), dihydro-favonol-4-reductase (DFR), leucoanthocyanidin oxygenase/anthocyanidin synthase (LDOX/ANS), and glycosyltransferase (UFGT) [9]. The genes encoding CHS, CHI, F3H, and F3ʹH are called early biosynthesis genes (EBGs), while those encoding DFR, ANS, and UFGT are late biosynthesis genes (LBGs). So far, more than 8000 flavonoids resulting from the modification of the core structure have been discovered in nature [10]. Both flavonoid O- and C-glycosides have been identified in crape myrtle [5, 7] (Fig. 1). In plants, two types of glycosyltransferases (GTs) can transfer the sugar moiety to the flavonoid aglycons: the cytoplasmic UDP-sugar dependent glycosyltransferase (UFGT), and the vacuolar acyl-glucose dependent glycosyltransferase (Glycoside Hydrolase Family 1 beta-glucosidase, GH1-GT) [11]. Methylation is also an important reaction involved in flavonoid biosynthesis. In high vascular plants, two types of O-methyltransferases (OMT) (metal independent, or dependent) have been identified to participate in the methylation of flavonoids. For example, VvAOMT in grape, AnthOMT in tomato, and PtAOMT and PsAOMT in *Paeonia* participate in anthocyanin/flavonoid biosynthesis [12–15]. The types and content of flavonoids depend on the activity or substrate specificity of the enzymes at the branch point and the O-, C-glycoside, and methylation of the core structure [9].

The mechanisms that regulate flavonoid/anthocyanin biosynthesis have been extensively studied. Different MYB transcription factors regulate EBG expression, while a ternary complex of a MYB, a basic helix-loop-helix (bHLH), and a WD40 repeat protein, known as MBW, controls LBG expression [16]. The type of MYB determines whether the MBW is a positive or negative regulator. Furthermore, MYB expression is tissue-specific and determines the content and distribution of the flavonoid/anthocyanin in a plant [17–20]. The WD protein (called TRANSPARENT TESTA GLABRA1, TTG1 in *Arabidopsis thaliana*) consists of 4–16 WD domain repeats without catalytic and DNA binding activity. Its propeller structures form a stable platform that can form complexes reversibly with bHLH and MYB [17]. Studies on *Arabidopsis thaliana* and *Arabis alpine* showed that TTG1 participates in anthocyanin and pro-anthocyanin biosynthesis, trichome and root hair differentiation, and seed mucilage deposition [17, 21]. In other plants, homologs of TTG1, such as *AN11* in petunia [22], *PgTTG1* in pomegranate (*Punica granatum* L.) [23], *VfTTG1* in fava bean (*Vicia faba* L.) [24], *OsTTG1* in rice [25], and *RsTTG1* in radish (*Raphanus sativus*) [26] are involved in anthocyanin biosynthesis. Loss-of-function of TTG1 results in plants lacking pigment accumulation in vegetable tissue or flowers.

In *L. indica*, some of the genes involved in anthocyanin biosynthesis have been identified based on transcriptomic data [2, 3, 27]. A recent study showed that a bZIP TF LfiHY5 and LfiMYB75 activate the anthocyanin biosynthesis in leaves of a crape myrtle varieties ‘Ebony Embers’ [28]; However, the mechanism of the biosynthesis of the different types of flavonoids in this plant should be elucidated (Fig. 1). Furthermore, *Lythraceae spp.* are widely distributed and economically significant; for instance, pomegranate and guava trees are used for fruit production, while *Heimia myrtifolia* and *Lythrum salicaria* flowers are used as medicinal herbs [1]. New genomic data will be helpful for molecular breeding through whole genomic selection in *L. indica.*

To further optimize the use of the resources of crape myrtle and related species, this study aims to decode the genome of the *L. indica* and its evolution history, identify the genes associated with flavonoids biosynthesis in crape myrtle, and elucidate the mechanism by which LiTTG1 regulates anthocyanin biosynthesis. The reference genome sequences obtained from this study can be used for evolutionary analysis of *Lythraceae spp.* and clarify the mechanisms by which the biosynthesis of medicinal compounds and ornamental traits are regulated.

## Results

### Genome sequencing, assembly, and annotation

**Fig. 2:**
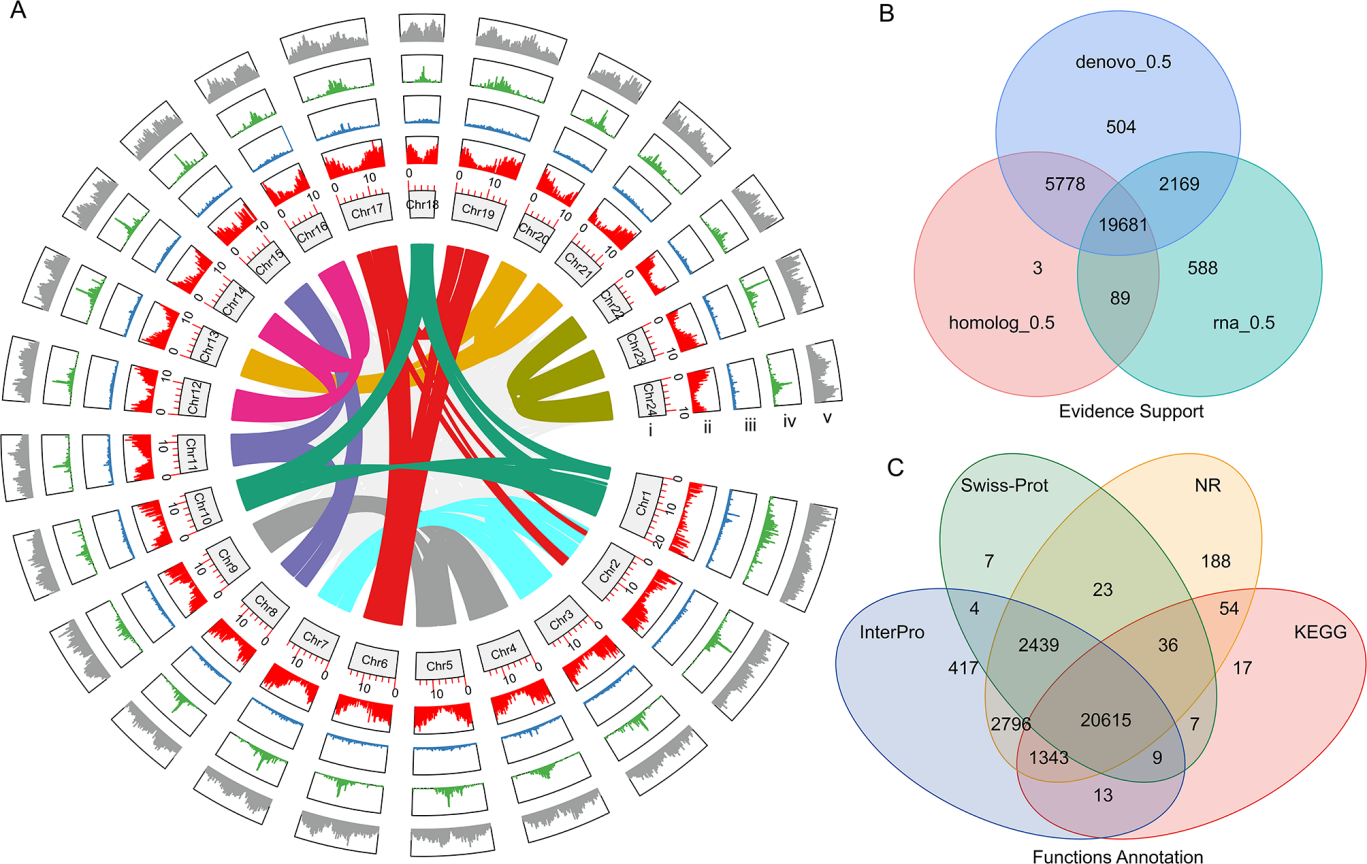
Genome structure and annotation of the *L. indica. ***(A)**, Circos map of the genome, including (i) the length of the 24 pseudo-chromosomes, (ii) the protein-encoded gene map, (iii) tandem repeat sequences, (iv) transposon-encoded proteins, and (v) transposons. The synteny region of the 24 pseudo-chromosomes is shown by different colors. Minor tick bar = Mb. **(B)** Gene prediction by three different methods. **(C)** Gene function annotation in different databases

In this study, we found the genome size of *L. indica* (NTU-1) (2n = 2x = 48) to be approximately 315 Mb to 326. 43 Mb based on flow cytometry and a 17-mer survey (Figs. S1 and S2). We further sequenced the plant’s genome using the PacBio Sequel platform, HiC, and Illumina PE150 and found the final assembled contig sequence to be 324.01 Mb, with heterozygosity and repeat contents of 1.65% and 29.17%, respectively (Figs. S1 and S2, Table 1). The assembly comprised 115 contigs with an N50 of approximately 4.14 Mb (Table 1) and was further assembled into 49 scaffolds. Among these, 98.21% (318.21 Mb) of sequences were used to construct 24 pseudo-chromosomes (Table 1; Figs. 2 and S3). The GC content of the entire genome was 38.64%, which is similar to the genome of the closest species of pomegranate (*P. granatum*) in *Lythraceae* [29, 30] (Fig. S4). The BWA software was used to align 95.42% of the Illumina short reads to the assembly, which covered 99.72% of the entire genome, of which 99.69% covered at least 4X, 99.66% at least 10X, and 96.1% at least 20X (Tables S1, S2). The Benchmarking Universal Single-Copy Orthologs (BUSCO) analysis results showed that 98.1% of the 1614 samples were complete single or duplicated BUSCO, 0.7% were fragmented, and 1.2% were missing BUSCO groups (Table S3). The results of the Core Eukaryotic Genes Mapping Approach (CEGMA; http://korflab.ucdavis.edu/datasets/cegma/) analysis showed that 93.95% of the core eukaryotic genes (233 out of 248) were complete genes (Table S4). In addition, 96.7% of the Illumina RNA-Seq reads obtained from four different tissues (shoot tip, shoot bottom mixed with leaves, flower bud, and flower petal) could map to the assembled genome. Collectively, the results suggest that a high-quality assembly of the *L. indica* genome was obtained.

**Table 1:** Summary statistics of the genome assembly and annotations of *L. indica*

Feature	Value
Estimated genome size (Mb)	326.43
Total size of assemble scaffold (Mb)	324.01
Number of scaffolds	49
Scaffold N50 (Mb)	$\sim$ 13.25
Longest scaffold (Mb)	$\sim$ 20.01
Total size of assembled contigs (Mb)	324.01
Number of contigs (≥ 1 kb)	115
Largest contig (Mb)	$\sim$ 12.61
GC content	38.64%
Heterozygosity	1.65%
Repeat content	29.17%
Protein coding genes (number)	28,811

To annotate the protein-coding genes in the genome of *L. indica*, de-novo-, homologs-, and transcriptome-based strategies were employed for prediction. In total, 28,811 protein-coding gene models were identified with an average gene length of 2,912.85 bp (Figs. 2 and S5, Tables S5 and 6). Mapping these genes on chromosomes showed that the gene density decreased from the telomeres to the centromeres on most of the chromosomes (Fig. 2A–ii). The total gene number was close to that of the sequenced genome of pomegranate (29,229) [29, 30], larger than that of *Psidium guajava* (25,601) [31], and significantly smaller than that of *Eucalyptus grandis* (35,931) (Table S6) [32]. Among the 28,811 genes, 27,968 (97.06%) were functionally annotated (Fig. 2C, Table S7). The KEGG analysis results showed that 22,094 (76.68%) of the annotated genes participated in special pathways, while the gene ontology (GO) analysis results showed that 17,514 (60.79%) genes were assigned to biological processes, cellular components, and molecular functions (data not shown). Non-coding RNAs accounted for approximately 0.1% of the *L. indica* genome and included 327 miRNAs, 552 tRNAs, 214 rRNAs, and 607 snRNAs (Table S8).

The *L. indica* genome included approximately 141.58 Mb of repetitive sequences, which accounted for 43.69% of the genome, of which 32.08% (approximately 103.26 Mb) were long terminal retrotransposons (LTR) and 11. 61% were other types of repeat sequences (Tables S9 and S10, Fig. S6). Transposons and transposon-coding proteins were found to be unevenly distributed among the 24 pseudo-chromosomes (Fig. 2A-iv and v), while tandem repeats were more or less evenly distributed throughout the genome (Fig. 2A-iii). These results indicate that LTR may have contributed to the evolution of the *L. indica* genome.

We found that the 24 pseudo-chromosomes could be divided into 8 groups according to their synteny relationship, each containing three chromosomes (Fig. 2A, inner circle). These results indicate that the *L. indica* genome exhibits hexaploidy to some degree.

### *L. indica* is a palaeohexaploid species

**Fig. 3:**
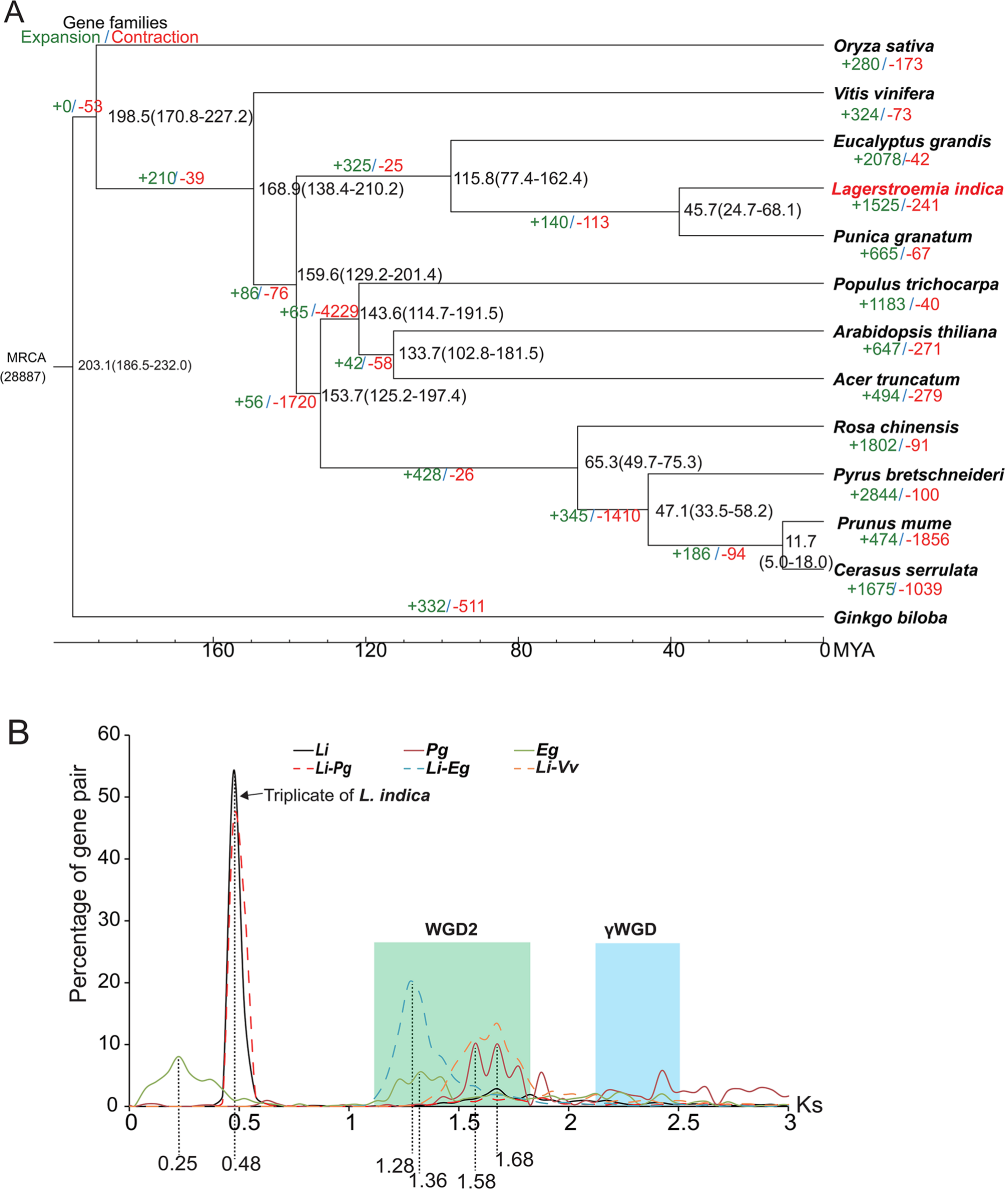
Evolution analysis of the *L. indica*. **(A)** The phylogenetic tree was constructed from a concatenated alignment of 327 single-copy gene families of 13 species. Gene family expansions and contractions are indicated in red and green, respectively. **(B)** Ks distribution of paralogs (intraspecies, solid lines) and orthologs (interspecies, dashed lines). *Li*, *Lagerstroemia indica*; *Pg*, *Punica granatum*; *Eg*, Eucalyptus grandis; *Vv, Vitis vinifera.* WGD, whole genome duplication. MRCA, the most recent common ancestor

A phylogenic tree of 13 species (details in materials and methods) was constructed using 327 single-copy gene families to disclose the evolution of *L. indica*, (Fig. 3). The tree shows that three *Myrtales* species (*E. grandis*, *Punica granatum*, and *L. indica)* are in one clade, which is a sister clade to the rosids species (such as *Populus trichocarpa*, *Arabidopsis thiliana*, *Rosa chinensis*, *Prunus mume)* (Fig. 3). This result is consistent with those of previous studies [31–33]. The divergence time between *Myrtales* and *Rosids*, *Eucalyptus grandis*, and *Lythraceae* species (*L. indica* and *Punica granatum*), were found to be approximately 159.6 (129.2–201.4), 115.8 (77.4–162.4), and 45.7 (24.7–68.1) million years (MYAs), respectively (Fig. 3A).

Of the 28,887 gene families found in the 13 species, 466 genes were found to be unique to *L. indica* (Fig. 3, Table S11). When five species (*Arabidopsis thaliana*, *Populus trichocarpa*, and three *Myrtale* species) were analyzed (Fig S7), 660 genes (in 345 gene families) were found to be unique to *L. indica*. The GO annotations for these 660 unique genes showed that they are enriched in molecular functions, including catalytic activity, RNA–DNA hybrid ribonuclease, hydrolase activity, and enzyme inhibitor; cellular components, including TRAPP complex-transport proteins; and biological processes, including cellular response to nitrogen starvation, auxin, and osmotic stress (Fig. S8). The KEGG pathway enrichment analysis of these unique genes indicated that they are involved in signal transduction pathways, such as environmental information processing, plant hormone, and phophadilinositol signaling; metabolic pathways, such as amino acid metabolism and phenylpropanoid and diterpenoid biosynthesis (Fig. S9). Except for the unique genes, 1521 and 242 gene families underwent expansion and contraction, respectively, in the crape myrtle genome compared with its most recent common ancestor (Fig. 3A). According to the KEGG pathway enrichment analysis, these expanded gene families are probably related to metabolic pathways, such as sugar, galactose, starch, sucrose, ascorbate, and sphingolipid metabolism; environmental adaption; plant–pathogen interaction; and enzymes, such as glycosyltransferase and kinase (Fig. S10). These unique and expanded genes provide insights into the biological activities of compounds in *L. indica.*

**Fig. 4:**
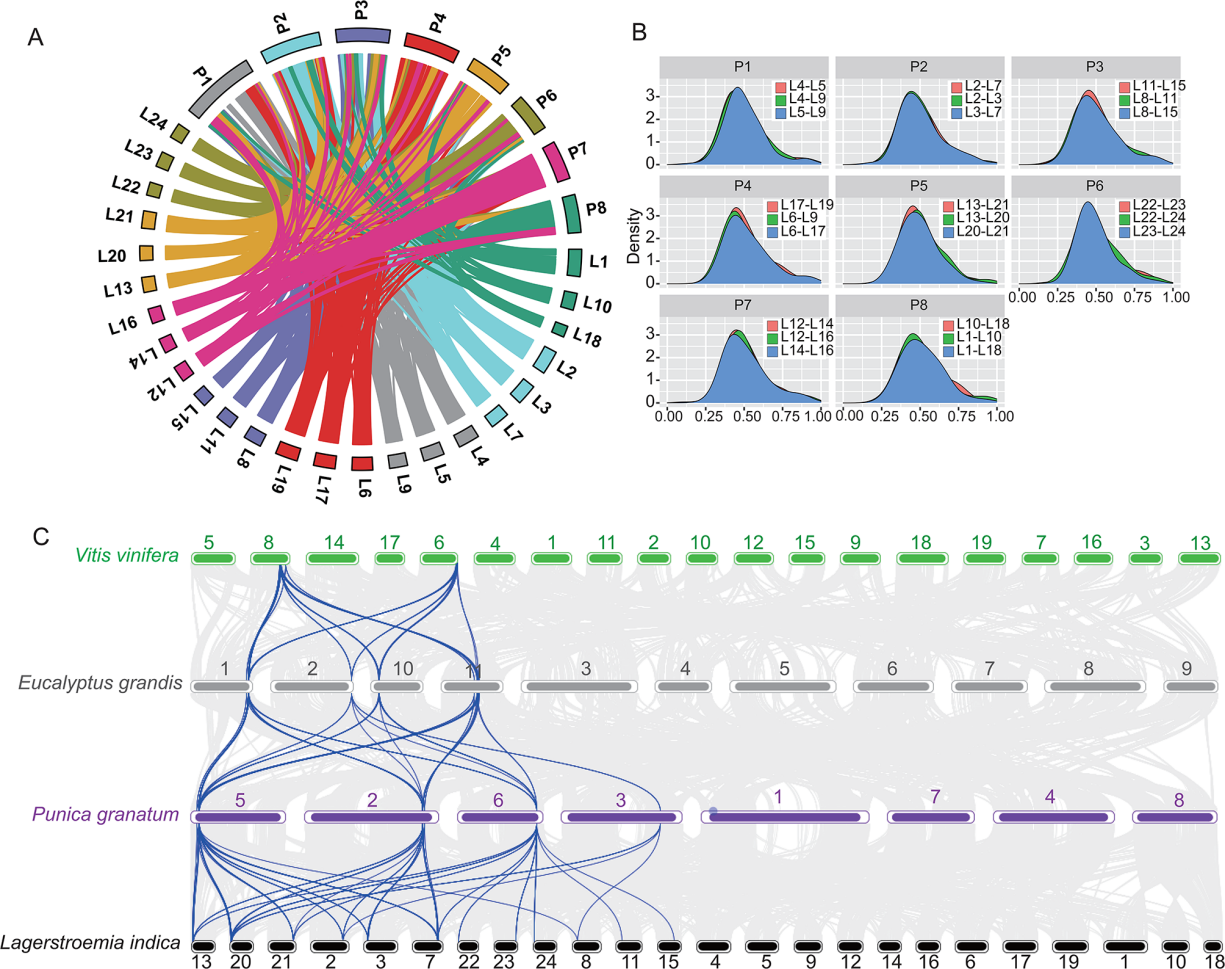
Genome collinearity among *L indica*, *Punica granatum*, *Eucalyptus grandis, and Vitis vinifera. ***(A) ***Lagerstroemia indica–Punica granatum*; **(B)** Ks plots of 8 groups of *L.indica*. **(C)** Genome collinearity among the four genomes

We calculated the synonymous substitutions per synonymous site (Ks) of paralogous and orthologous genes of *L. indica*, *Punica granatum* (pomegranate), *Eucalyptus grandis* (eucalyptus), and *Vitis vinifera* (grape) to clarify the whole genome duplication (WGD) of the *L. indica* genome. All three *Myrtales* species showed several median Ks peaks. The Ks of angiosperms were between 2 and 2.5, indicating the palaeohexaploidy of the WGD event (γWGD) [34], and those of eucalyptus and pomegranate were approximately 1.5, indicating the reported previously linkage-specific WGD event (WGD2) [29, 32]. Further, in *L. indica*, the median Ks of more than 50% of the paralogs were 0.48, while those of about 5% of the paralogs were 1.68, indicating that they experienced another WGD event that is more recent than those that occurred in pomegranate and eucalyptus. This recent WGD event was superimposed on the former WGD2 event (Fig. 3B). Combining the hexaploidy characteristic of the genome (Fig. 2A), we suggest a triplicate of WGD events to have occurred in *L. indica* at approximately 38.5 MYA according to the divergence time in Fig. 3A. To verify this hypothesis, we compared the chromosome synteny relationship between pomegranate and *L. indica* and found that the three chromosomes in a group of *L. indica* was collinear with same chromosome of pomegranate (Fig. 4A and S11), and also the Ks plots of each chromosome in a homologous group are very similar (Fig. 4B). From the synteny relationship among the four species (*Vitis vinifera, E. grandis, Punica granatum*, and *L. indica*) (Fig. 4C), we found that the chromosome blocks maintain a ratio of 2:4:4:12. For example, the two terminal ends of *Vv_chr6* and *Vv_chr8* are collinear to the four sites of *E. grandis* (*Eg_chr1, Eg_chr2, Eg _chr10, and Eg _chr11*) and *Punica granatum* (*Pg_chr2*, *Pg_chr3*, *Pg_chr5*, and *Pg_chr6*), respectively. In *L. indica*, 12 chromosomal loci (*Li_chr13, Li_chr20, Li_chr21, Li_chr2, Li_chr3, Li_chr7, Li_chr22, Li_chr23, Li_chr24, Li_chr8, Li_chr11, and Li_chr15*) (Fig. 4C) were found to be collinear to the ends of *Vv_chr6* and *Vv_chr8* (Fig. 4C). The gene numbers retained at each cognate locus in *L. indica* were reduced, indicating that although the genome of *L. indica* was triplicated, many genes were purged during the genome diploidization. Therefore, *L. indica* is a palaeohexaploidic species (Figs. 2A and 3B, and 4).

### Anthocyanin content and composition of three petal types are different

**Fig. 5:**
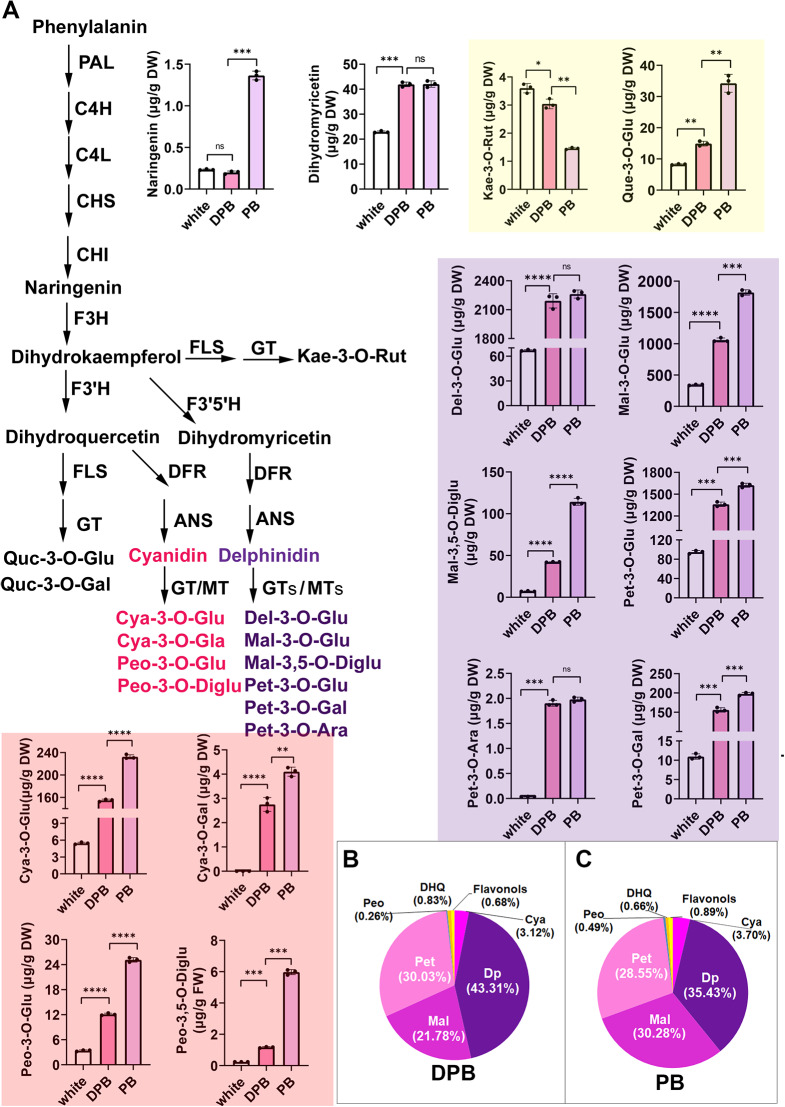
The composition of anthocyanin and other flavonoids in three differently colored petals of *L. indica. ***(A)** contents of different flavonoids. The reactions of enzymes are shown on the pathway. **(B)** and **(C)** Percentages of different flavonoids in **(B)** DPB and **(C)** PB petals. Mean ± SD, *n* = 3, *p < 0.05, **p < 0.01, ***p < 0.001, ****p < 0.0001 (*t*-test). PAL, phenylalanine ammonialyase; C4H, cinnamate 4-hydroxylase; 4CL, 4-coumarate CoA ligase; CHS, chalcone synthase; CHI, chalcone isomerase; F3H, flavanone 3-hydroxylase; F3’H, flavonoid 3’-hydroxylase; F3’5’H, flavonoid 3’5’-hydroxylase; FLS, flavonol synthase; FNS, flavone synthase; DFR, dihydroflavonol-4-reductas; LDOX/ANS, leucoanthocyanidin oxygenase /anthocyanidin synthase; GTs, glycosyltransferases; MTs, methyltransferases; . DHQ, dihydroquercetin; Peo, peonidin derivative; Pet, petunidin derivative; Mal, malvidin derivative; Dp, delphinidin derivative; Ara, arabinoside; Gal, O-galactoside; Glu, glucoside; Rut, rutinoside; DPB, Deep purplish pink;PB, purple

Previous reports showed that anthocyanins are the major inherent pigment in petals [3, 5] and that the color of petals is affected by the pH value of the vacuole, as well as by the shape of the epidermal cells. To elucidate the mechanism that regulates anthocyanin biosynthesis in *L. indica*, we analyzed the anthocyanin/flavonoid composition and content in the white, PB, and DPB flowers using UPLC and tandem mass spectrometry (MS/MS). Altogether, 44 different flavonoids/anthocyanidins were detected in the three petal types (Table S12). Of these, 10 types of anthocyanidins, constituting more than 1 µg per gram of dry weight, were detected (Table S12, Fig. 5). In the white flower, the total anthocyanin/flavonoid content was approximately 8.9% and 11.34% of that in DPB and PB, respectively; however, the composition of the anthocyanidins was almost the same. The total content of anthocyanins in DPB was approximately 80% of that in PB which included four types of delphinidin- and cyanidin-based pigments. Blue or purple delphinidin (Dp) derivative reached 95.12% and 94.26% in DPB and PB, respectively (Fig. 5). However, cyanidin-based (Cy) anthocyanins constituted no more than 4% of total anthocyanin. In addition to the composition, the ratios of delphinidin and malvidin (Mv) in DBP and PB were different: 43.31% Dp and 27.78% Mv in DBP (Fig. 5B), and 35.43% Dp and 30.28% Mv in PB (Fig. 5C). The peonidin derivatives were first discovered in *L. indica*, although their contents were negligible (Fig. 5A). In addition to anthocyanins, traces of naringenin, dihydroquercetin, and flavonols (e.g., quercetin, kaempferol, and rutin) were found in all three petals. The levels of anthocyanin precursors (dihydroquercetin, naringenin, and dihydrokaempferol (DHK)) were low in all three petal types. Based on the content, compositions, and ratio of the different flavonoids/anthocyanidins, we concluded that: (i) LiDFR preferentially metabolizes dihydromyricetin (DHM), because dihydroquercetin- and dihydrokaempferol-based pigments did not exceed 4% (Fig. 5, Table S12); (ii) Almost all precursor substances are converted as evidenced by the low levels of naringenin; (iii) The white color in flowers was attributed to their low anthocyanin contents, however, the content and ratio of Dp3G and Mv3G in DPB and PB petals were significantly different. The intracellular pH of the three petal types was between 4.10 and 4.23, with that of the white petal being slightly higher than that of DPB and PB (data not shown). Further in vitro experiments should be conducted using contents and composition of Dp3G and Mv3G that are similar to those in vivo to confirm that this tiny discrepancy could lead to color change. Scanning electron microscopy showed that the epidermal cells of the three petal types were multiangular and irregular. These results indicate that the contents and ratio of Dp3G and Mv3G were the main factors that contribute to the different colors of DPB and PB.

### Genes participate in flavonoid/anthocyanin biosynthesis

The four classes of flavonoids in crape myrtle (Fig. 1), the different compositions of anthocyanidin in the three differently-colored petals (Fig. 5), and the enrichment of phenylpropanoid biosynthesis genes (Fig. S9) indicate the importance of flavonoid biosynthesis in *L. indica*. Hence, we identified the genes involved in the flavonoid/anthocyanidin biosynthesis at the genome level. We divided the pathway into five parts: phenylpropanoid pathway, flavonoid skeleton biosynthesis, anthocyanin branching pathway, other flavonoid pathways, and modification pathway.

#### Phenylpropanoid pathway (PA pathway)

The PA pathway includes 3 enzymes: Phenylalanine ammonialyase (PAL), Cinnamate 4-hydroxylase (C4H), and 4-coumarate CoA ligase (4CL). The products of the pathway are the precursors of various plant-specific metabolic pathways, such as those of anthocyanin, flavonoids, lignin, and alkaloid biosynthesis.

**Fig. 6:**
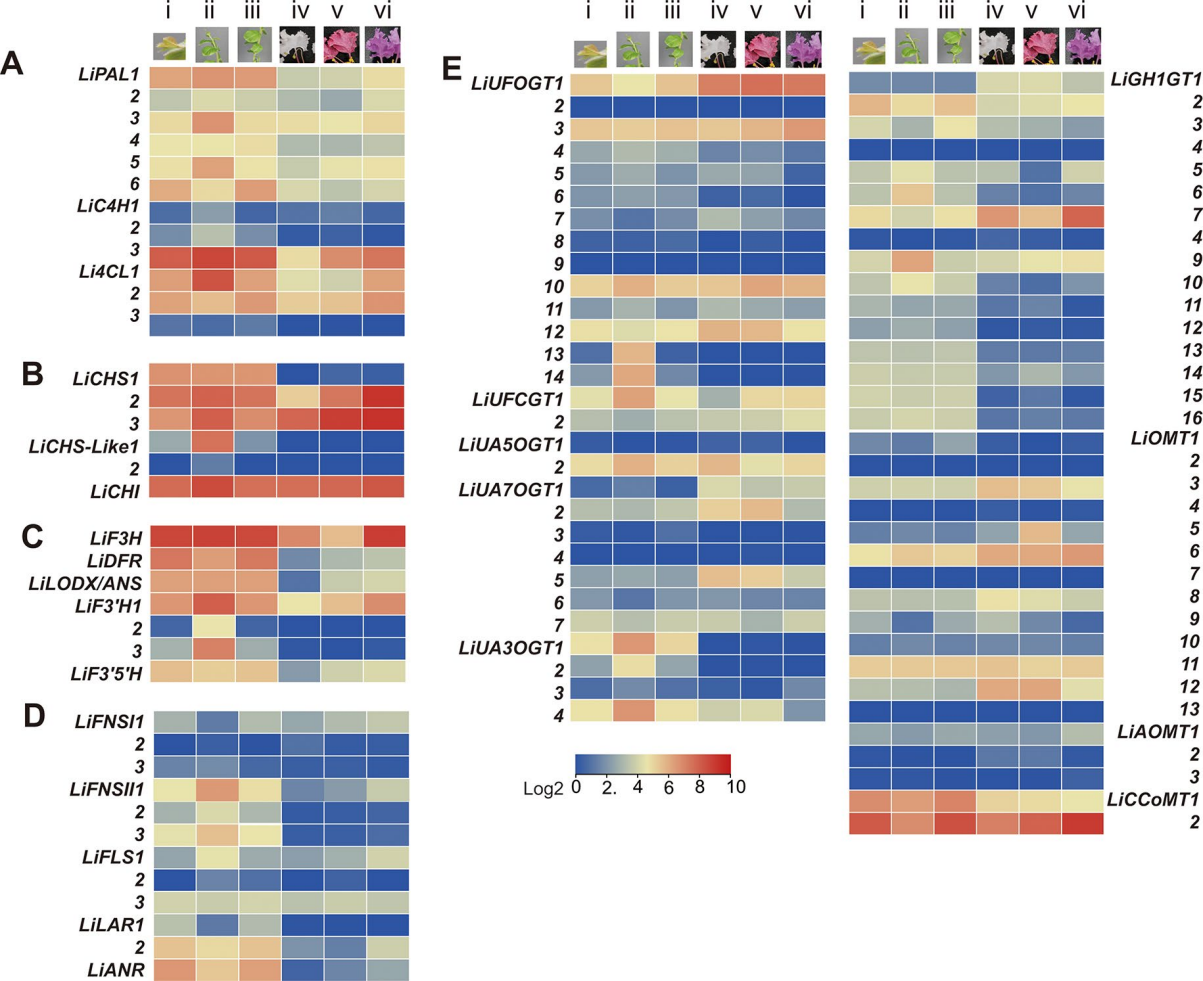
Expression profiles of putative flavonoid biosynthesis genes. The tip (i), up (ii), and bottom (iii) parts of the young shoots (*L. indica* var Ebony Embers “pure white”); Three local varieties with different petals color: white (iv), DPB (68A) (v) PB (75A) (vi). **(A)** Phenylpropanoid pathway. **(B)** flavonoid skeleton biosynthesis. **(C)** Anthocyanin branching pathway; **(D)** Other flavonoid pathways. **(E)**Modification pathway. PAL, phenylalanine ammonialyase; C4H, Cinnamate 4-hydroxylase; 4CL, 4-coumarate CoA ligase; CHS, Chalcone synthase; CHI, Chalcone isomerase; F3H, Flavanone 3-hydroxylase; DFR, Dihydroflavonol-4-reductase; LDOX/ANS, Leucoanthocyanidin oxygenase /anthocyanidin synthase; F3’H, Flavonoid 3’-hydroxylase; F3’5’H, Flavonoid 3’5’-hydroxylase; FNS, flavone synthase; FLS, flavonol synthase; LAR, Leucoanthocyanidin reductase; ANR, Anthocyanidin reductase; UFOGT, UDP-glucose: Flavonol O- glycosyltransferases; UFCGT, UDP-glucose:Flavonol C-glycosyltransferases; UA5OGT, UDP-glucose: anthocyanin-5-O glycosyltransferase; UA7OGT, Anthocyanin 3’ or 7-O-glycosyltransferase; UA3OGT, Anthocyanidin 3-O-glycosyltransferase; GH1-GT, glycoside hydrolase family 1 glycosyltransferase; OMT, O-methyltransferase; DPB, deep purplish pink; PB, purple

Six LiPALs with more than 60% similarity to EgrPAL*s* and AtPALs were identified (Fig. S12). According to the NJ phylogenic tree, three *LiPALs* (*LiPAL3*, *-4*, and *-5*) are clades of *EgrPAL1* located on the homologous chromosomes 2, 3, and 7 (L2, L3, and L7) of *L. indica* (Fig. 4, Table S13), indicating that these genes remained intact during the evolution of *L. indica*. The remaining *LiPALs* are orthologs of *EgrPAL* that were also found on the homologous chromosomes. RNA-seq experiments showed that all six *LiPALs* were constitutively expressed in all tissues, where the expression of *LiPAL1* and *LiPAL6* was relatively high in green tissues while that of *LiPAL2* was the lowest (Fig. 6 and S12).

Three *C4H* genes can be divided into two classes in *L. indica* (Fig. S12). *L. indica*, *Punica granatum*, and *Eucalyptus grandis* were found to maintain one copy of a class II member, while there were two *C4H* orthologs of *EgrC4H1* in pomegranate and *L. indica*. The expression of the *LiC4H3* was several dozen-fold higher than that of *LiC4H1* and *2* in all tissues (Fig. 6 and S12).

Altogether, three *Li4CLs* belonging to types I (lignin biosynthesis) and II (phenylpropanoids derivatives other than lignin) were found in the *L. indica* genome. RNA-Seq experiments showed that *Li4CH1* and *2* were highly expressed, while *Li4CH3* expression was not detected (Fig. 6 and S12). This result indicated that *L. indica* maintained one active 4CL for the biosynthesis of lignin and phenylpropanoids derivatives. The expression of *Li4CL1* (Type II) was lower than that of *Li4CL2* (Type I) in the flower but higher in young shoots (YS) (Fig. 6 and S12), which can be attributed to the high demand for lignin biosynthesis during growth.

#### Flavonoid skeleton biosynthesis

CHS catalyzes the first commit step from activated 4-coumaroyl-CoA to flavonoid compound, and the CHI closed the C ring of the flavonoid C6-C3-C6 skeleton (Fig. 1). We identified five putative *LiCHSs* and one *LiCHI* gene in *L. indica*. The phylogeny tree shows that only three *LiCHSs* are in the same clade as *AtCHS*, while the others belong to a different group. *LiCHS1-3* is highly expressed in YS, but *LiCHS1* expression was not detected in blooming flowers (Figs. 6 and S13). The expression of two *LiCHS-lik*e genes was not detected in six tissues, except the upper part of the YS. Therefore, the three *LiCHSs* participate in flavonoid biosynthesis in all tissues, while the two *LiCHS-like* genes probably function under special conditions (Figs. 6 and S13). The expression of *LiCHI* is relatively stable in different tissues. This result indicates that *LiCHI* is not the main regulator of flavonoid biosynthesis in *L. indica* (Figs. 6 and S13).

#### Branches of the anthocyanin biosynthesis pathway

Naringenin is metabolized into several flavonoid compounds by different enzymes. Anthocyanidin is one of the four types of flavonoids in *L. indica* (Fig. 1). There are at least three enzymes (F3H, DFR, ANS) downstream of the CHI for the synthesis of anthocyanidins. Since F3H, ANS, FNS I (flavone synthesis, FNS), and FLS belong to the 2-oxoglutarate-dependent dioxygenase superfamily, and F3H and FNS I compete for the same substrate [35], we detected both *F3H* and *FNSI*. Only one gene was found to be phylogenetically close to *VvF3H* and *AtF3H* (Fig. S14), the rest belonged to the same clade as *AtDMR6* (downy mildew resistant 6, named *LiFNSI*) which may be involved in the hydroxylation of salicylic acid at the C-5 position [35]. *LiF3H* is highly expressed in six tissues, particularly in the YS and PB petals. However, the three *LiFNSI* were expressed in YS but not in flowers. Only one DFR and ANS gene were identified in the *L. indica* genome. Their expression is almost undetectable in the white blooming flower and is relatively high in YS and colorful petals (Figs. 6 and S14).

Flavonoid 3’-hydroxylase (F3’H) and flavonoid 3’5’-hydroxylase (F3’5’H) catalyzed the hydroxylation at the C3’ and C3’5’ positions, respectively, and they determined the diversity of the product, such as red anthocyanin pigment cyanidin and purple or blue pigment delphinidin (Fig. 5). In *L. indica*, there are three members of *LiF3’H* and one *LiF3’5’H*, respectively. *LiF3’H1* is highly expressed in colorful petals and YS, while *LiF3’H2* and *3* are only expressed in the upper part of the YS. Furthermore, the expression of *LiF3’5’H* is lower than that of *LiF3’H1*. Interestingly, although *LiF3’5’H* expression was low, the levels of its products (delphinidin and its derivatives) were high (Figs. 5 and 6, and S15). This can be attributed either to the higher catalytic activity or the higher efficiency of downstream enzymes of LiF3’5’H compared to that of LiF3’H1.

#### Genes related to Flavone, Flavonol, and Flavanol pathway

FNS, FLS, LAR, and ANR catalyze the biosynthesis of flavone, flavonol, and flavanol, respectively (Fig. 5). We identified three *LiFNSIIs*, three *LiFLSs*, two *LiLAR*s, and one *LiANR* in the genome of *L. indica*. The expression of *LiFNS1-3, LiLAR1-2*, and *LiANR* in the YS exceeds that in flower petals, while that of *LiFLS3* is similar in all tissues (Figs. 6 and S16, Table S13). Generally, the expression of these genes in petals is low but high in vegetable tissue, indicating higher levels of flavone, flavonol, and flavanol than anthocyanin in green tissues (Table S12, Fig. S16) and low levels in the petals (Fig. 5, and Table S12). This justifies why the leaves of *L. indica* are used in traditional herbal medicine.

#### Modification pathway

The glycosidation of flavonoids at the hydroxyl or C-C bond by GTs increases their stability. Usually, the sequence similarity of GT orthologs among plants is relatively low. The putative GTs and their classes are presented in Table S14, and their phylogenic relationship is shown in Figs. S17 and S18. Of the 45 different GTs, only four *LiUFOGT* (UDP-glucose: Flavonol O- glycosyltransferases, UFOGT) (*LiUFOGT 1, 3, 10, 12*), LiUFCGT2 (flavonoid O or C-glycosyltransferases), *LiUA5OGT2* (anthocyanin 5 -O-glycosyltransferases, *UA5OGT*), three *LiGH1GT* (Glycoside hydrolase family 1 glycosyltransferase) are highly expressed in all six tissues (Fig. 6). Three *LiUA7OGT* (1, 2 and 5) (anthocyanin 3’ or 7-O-glycosyltransferases, *LiUA7OGT* for simple) are specifically expressed in flowers, while the *LiUA3OGTs* (anthocyanin 3-O-glycosyltransferases, UA3OGT) are mainly expressed in green tissues. This expression pattern could explain the glycoside diversity in the different parts of *L. indica* (Fig. 1) but not between the three different petals.

Methylation of the 3’ or 3’5’ hydroxyl of anthocyanin (Fig. 1, B ring) is catalyzed by S-adenosyl-L-methionine (SAM)-dependent O-methyltransferases (OMTs) (EC 2.1.1). Altogether, eighteen OMTs were identified in *L. indica*. Phylogenic analysis showed that these *LiAOMTs* belong to the two subclasses reported in other plants [36], and we named these genes according to their orthologs in the subclade (Table S14, Fig. S19). The transcriptional profiles show that two *LiCCoAOMTs* (class I) and *LiAOMT6* and *11* (class II) are highly expressed in all tissues, while *LiOMT-9* and*-12* are highly expressed in blooming flowers (Table S14, Fig. S19).

Collectively, the expression of the anthocyanin branching pathway genes does not correlate with the anthocyanin content of the petal. The higher expression of these genes in YS (Fig. 6) indicates the antioxidant function of the flavonoid, the growing demand for lignin, and the second cell wall.

### MYB gene family

Using a combination of several methods (Materials and methods), we identified 137 members of MYB with intact R2R3 motifs (Table S15). A phylogenetic tree of *AtMYBs* (137), *EugrMYBs* (147), and *LiMYBs* (137) MYBs showed that most subgroups have corresponding orthologs in three species, except for six subgroups that were absent in crape myrtle (Table S16) and *LiMYB128* and *LiMYB134* that did not belong to any subgroup (SG). Of the six WPS MYBs (woody-preferential subgroup), five WPSs were found in *L. indica* (Fig. S20, Table S16). Previous studies have shown that the expression of SG5 and SG6 R2R3 MYBs participating in lignin and other phenolic compound biosynthesis was upregulated in woody plants [37, 38]; however, this is not the case for SG6 in *L. indica.* The intra-species collinearity analysis showed that the cognate *LiMYBs* were linked to 24 pseudo-chromosomes and were divided into eight groups (Fig. S21). This result is in line with the whole genome collinearity relationship (Fig. 2). Interestingly, we found that approximately 84% of the genes were duplicated and only 16% had more than two copies (for instance, evm.model.Chr15.444/ LiMYB93 collinearity with that of evm.model.Chr8.905 /LiMYB64 and evm.model.Chr11.918/ LiMYB76) (Fig. S21). This indicates that although *L. indica* exhibited hexaploidy, the low frequency of the three copies of the homologous genes was maintained during evolution.

#### Co-expression of *LiMYBs* and the flavonoid biosynthesis genes

**Fig. 7:**
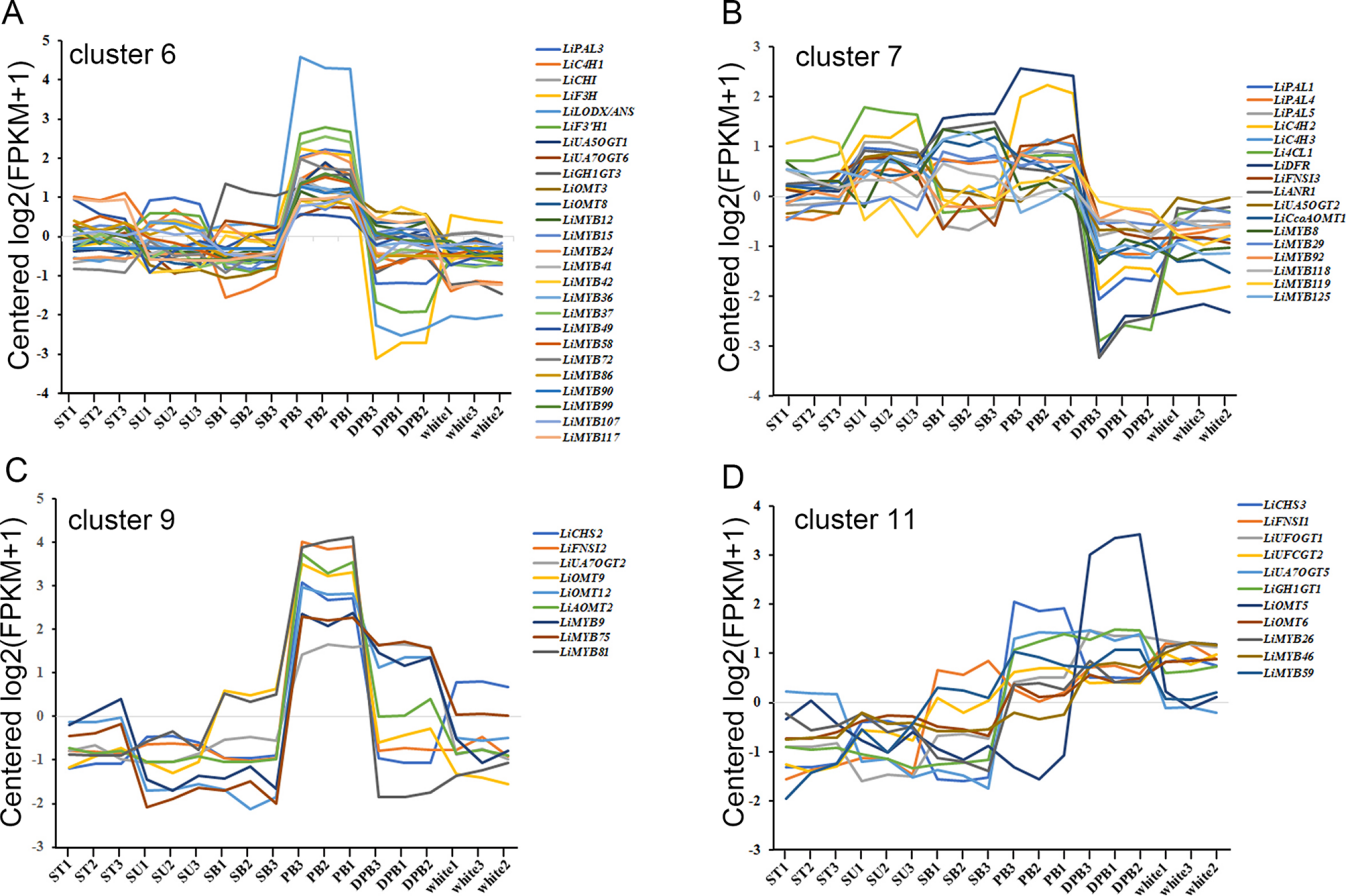
The co-expression patterns of the flavonoid pathway and the LiMYB genes. **(A)** cluster 6; **(B)** cluster 7; **(C)** cluster 9; **(D)** cluster 11. All the data were obtained from the whole genome transcriptional co-expression analysis illustrated in Fig. S22. Abbreviations of gene names are shown in Fig. 6

Many R2R3 *MYB* genes have been reported to regulate the expression of the flavonoid biosynthesis pathway in higher plants. To excavate the *MYB* members that may participate in flavonoid biosynthesis in *L. indica*, we analyzed the co-expression patterns of differently expressed genes (DEGs) of the transcriptome data of six tissues. Altogether, fourteen clusters of DEGs were identified (Fig. S22); cluster 6 contained genes such as PAL, C4H, CHI, ANS/LODX, GTs, and MTs related to flavonoid biosynthesis and modification; cluster 7 exhibited PA pathway and branch pathway genes (e.g., FNS); and clusters 9 and 11 contained CHS, FNS, and modification genes (Figs. 7 and S22). In these 4 clusters, 28 R2R3 MYBs, which belong to 14 different subgroups (Table S17), co-expressed the flavonoid-related genes. The expression levels of genes in cluster 6 were higher in PB petals than in other tissues and low in DPB and white petals in cluster 7. The expression of genes in cluster 9 was high in the PB flowers, moderate in the DPB flowers, and low in the white flowers as well as in the YS. The expression of genes in cluster 11 was higher in the three different flowers than in the YS (Figs. 7 and S22). Therefore, from the co-expression patterns of the anthocyanin biosynthesis genes and the MYBs, we found that the MYBs in clusters 6 and 9, which belong to S2, S3, S4, S6, and S20 ($\sim$ 10 members) may positively regulate the gene expression and the anthocyanin content. On the other hand, genes related to modification are regulated coordinately with the biosynthesis genes.

### LiTTG1-1 inhibits anthocyanin biosynthesis

**Fig. 8:**
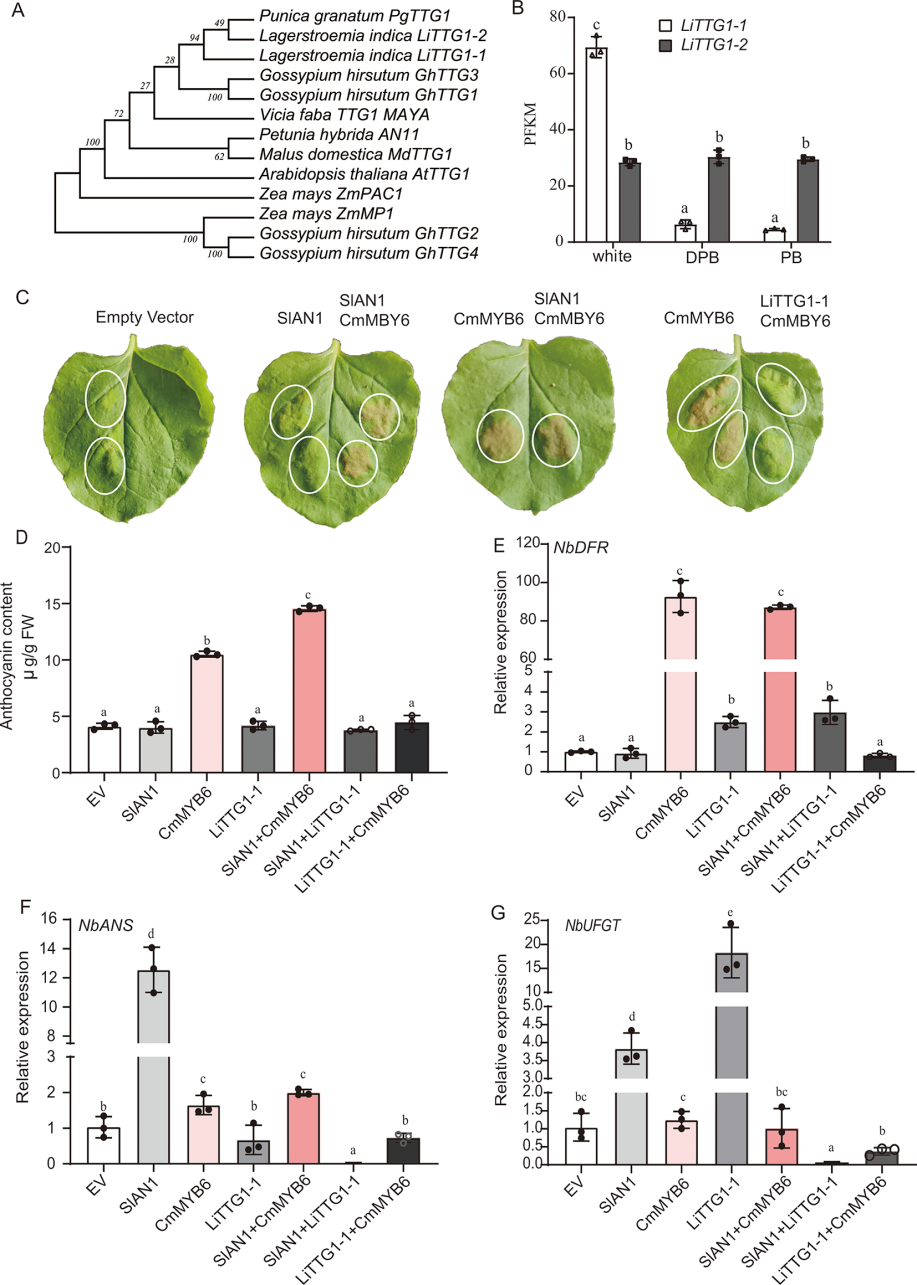
Characteristics and function of LiTTG1-1. **(A)** A neighbor-join tree of the TTG1 proteins involved in flavonoid biosynthesis. AtTTG1, *Arabidopsis thaliana*, accession number AJ133743; GhTTG, *Gossypium hirsutum* (GhTTG1, accession number AF336281; GHTTG2, accession number AF530912; GhTTG3, accession number AF530911; GHTTG4, accession number AF530910); MdTTG1, *Malus domestica*, accession number AF220203; LiTTG1, *Lagerstroemia indica*. PhAN11, *Petunia hybrida*, accession number U94748; PgTTG1, *Punica granatum* (pomegranate), accession number HQ199314; *Vicia faba*, VfTTG1, accession number MN119531; *Zea mays*, PAC1, accession number AY115485; *Zea mays*, MP1, accession number AY339884. **(B)** Transcriptional level of *LiTTG1*s among the three colored petals. FPKM, fragments per kilobase million. **(C)** The phenotype of tobacco leaves. The injection combinations were indicated above the leaves and circles indicated the injection zone. **(D)** Total anthocyanin. **(E–G)** Relative expression levels of the *NbDFR***(E)**, *NbANS***(F)**, and *NbUFGT***(G)**. Internal control, Actin gene; Sample control, empty pWM101 vector. Mean ± SD. All data were collected from at least three individual plants and three technical repeats. One-way ANOVA was used to analyze differences among samples

Due to the conserved functions of TTG1 homologs among plant species, we used AtTTG1 and PgTTG1 as templates to conduct a BLAST search on the *L. indica* genome and found two proteins with high similarity among them. The neighbor-joining phylogenic tree shows they were in the AtTTG1 subclass, but not in the maize MP1 subclass (Fig. 8A). Hence, they were named LiTTG1-1 (evm.model.Chr19.137) and LiTTG1-2 (evm.model.Chr17.140), respectively (Fig. 8A). Transcriptomic analysis results showed that *LiTTG1-1* expression is negatively correlated with total anthocyanin in flower petal (*r* = -0.981, Pearson Correlation Coefficient) (Figs. 5 and 8B), while *LiTTG1-*2 expression was similar among the petals of the three different flower types. Hence, the function of the *LiTTG1-1* gene was further investigated.

We used the tobacco transient assay to explore the function of LiTTG1-1. In this experiment, CmMYB6 and SlAN1 were used as positive controls since they upregulate anthocyanin biosynthesis in tobacco [39, 40]. As anticipated, CmMYB6 induced anthocyanin biosynthesis alone and in combination with SlAN1 (Fig. 8C and D). Surprisingly, we found that LiTTG1-1 eliminated the effect of CmMYB6. Further, the qPCR results showed that CmMYB6 induced *NbDFR* and *NbANS* expression, SlAN1 induced *NbANS* and *NbUFGT* expression, and LiTTG1-1 induced *NbDFR* and *NbUFGT* expression. However, when LiTTG1-1 was co-transfected with CmMYB6, the expression of the three genes was similar to that when using the empty vector (pWM101). The transfection of SlAN1 with LiTTG1-1 weakly upregulated *NbDFR* expression and strongly down-regulated *NbANS* and *NbUFGT* expression (Fig. 8E-G).

We also induced the ectopic overexpression of *LiTTG1-1* in *Arabidopsis* and found that the colors of the seed coat and YS of the WT and LiTTG1-1 lines were similar. These results indicated that LiTTG1-1 does not negatively regulate anthocyanin or proanthocyanin biosynthesis in *Arabidopsis* (Fig. S23).

Collectively, since *LiTTG1-1* expression reduced anthocyanin levels in the flower petals of *L. indica* and antagonized the effects of CmMYB6 and SlAN1, LiTTG1-1 can be considered as a repressor of anthocyanin biosynthesis.

## Discussion

### Palaeohexaploid of *L. indica*

In this study, we sequenced the genome of *L. indica* using a combination of several next-generation sequencing technologies. By comparing the synteny relationship between inter- and intra-species (grape, eucalyptus, pomegranate, and crape myrtle) (Figs. 2, 3 and 4), we found that *L. indica* is a palaeohexaploid species and that the *L. indica* triplication occurred after the divergence of the pomegranate and crape myrtle (38.5 MYA). Early chromosome number analysis indicated that the basic chromosome number of *Lythraceae* is eight [41, 42] and that more than half of the *Lythraceae* genera are polyploids without apparent close diploid relatives [43]. *Lagerstroemia* and *Duabanga* may share a tetraploid ancestor because they are sister genera based on the phylogenetic analysis of the chloroplast (cp.) *rbcL* gene and nuclear rDNA internal transcribed spacer (ITS) and have the same chromosome numbers (2n = 48) [43, 44]. The genomic data presented in this study strongly indicate that *L. indica* is a palaeohexaploid, not a palaeotetraploid. Zhou et al. [45] have recently published the results of their genomics study on *L. indica*, showing findings similar to those of our study regarding the evolution of this species.

### Downsizing of the genome after triplication of *L. indica*

The assembled genome of *L. indica* is 324.01 Mb (99.2% of the whole genome), which is similar to that of pomegranate (320 Mb) [33]. An interesting phenomenon of *L. indica* is the hexaploidy of its chromosome number despite the small size of its genome. This indicates that the chromosomes of *L. indica* did not experience fusion, but many duplicated sequences were lost (downsized) after the whole genome was triplicated. The decreased gene number at collinearity loci of *L. indica*, including copies of the genes involved in flavonoid biosynthesis (Figs. 4 and 6, Table S13) and those that encode MYB transcription factor, indicate the downsizing of the genome (Fig. S22, Table S16). Genome downsizing after polyploidy is very common in angiosperms [46]. This phenomenon explains the natural selection of flowering species with low requirements for nitrogen (N) and phosphate (P) due to their small-sized genome, thereby promoting CO_2_ uptake and accelerating the response to low environmental moisture [46, 47]. According to the gene balance hypothesis, products of the gene that form the components of the signal transduction pathway, and transcription factors are retained after WGD [48]. In a previous study, we found that approximately 80% of the recently duplicated pairs of the *LiCIPKs* (CBL-interacting protein kinase (CIPK); involved in calcium signaling) genes are preserved [49]; however, only five out of fifteen carotenoid cleavage oxygenase genes were duplicated (manuscript submitted). In this study, approximately 50% of the R2R3 *LiMYB* members were found to have a synteny homologous pair. These findings suggest that genes retained in the *L. indica* genome also followed some of the common laws of angiosperms during the genome downsizing. In the future, further studies on *Myrtales* species are required to elucidate the genome evolution of *L. indica*.

### The mechanism of petal color regulation in *L. indica*

In this report, we detected the content and composition of anthocyanin in blooming petals of white, PB, and DPB flowers (Fig. 5), the expression profiles of anthocyanin biosynthesis-related genes (ABGs) (Fig. 6), the co-expression patterns of the R2R3 MYB and ABGs (Fig. 7), and the function of LiTTG1-1 using transient assay. We investigated the effects of different combinations of three components in the MBW complex and the activity or substrate specificity of enzymes, and the results showed that the anthocyanin biosynthesis in *L. indica* is regulated at the transcription level.

The expression of all ABGs except for *LiCHI* and *LiF3H* was found to be associated with the content of anthocyanin (Figs. 5 and 6). In higher plants, the MYB-bHLH-WD40 triple complex (MBW) is the conserved regulating modular of ABG expression. Among the MBW, MYB TFs are the most sophisticated factors, as they could act as activators/repressors and/or targets of the upstream signal [9, 50]. From the co-expression patterns of *LiMYBs* and all the flavonoid-related genes, MYBs in clusters 6 and 9 may be positive regulators (Figs. 7 and S22). The phylogenic tree showed that these MYBs belong to the SG2 and SG3, SG6, and WSP sub-groups (Fig. S20, Tables S15 and S16). SG6 MYBs are well-known anthocyanin/flavonoid biosynthesis genes, of which *LiMYB72* (SG6 type) is co-expressed with several ABG genes. SG2 and SG3 type MYBs, such as AtMYB15 (AT3G23250), AtMYB58 (AT1G16490), and AtMYB63 (AT1G79180) participate in the lignin or monolignol biosynthesis [50–52]. Regarding woody plants, EgMYB1 and EgMYB88 (WPS-I group) in *E. grandis* [53, 54] and PtrMYB221 in hybrid poplar [55] are involved in the biosynthesis of phenylpropanoid-derived secondary metabolites including lignin. The *PAL*, *C4H*, *4CL*, *OMT*, and *CCoMTs* are the upstream genes for the biosynthesis of the phenylpropanoid-derived compounds (lignin, monolignol, flavonoid, etc.). Further studies are required to clarify how these LiMYBs fine-tune the expression of phenylpropanoid pathway genes in *L. indica*.

In the MBW complex, TTG1(WD40 protein) is relatively conserved. The AtTTG1 and its homologs were reported to enhance anthocyanin biosynthesis in different plants. In contrast to the effect of TTG1 in *Arabidopsis* [56], maize [57], petunia [22], radish [26], and rice [25], LiTTG1-1 repressed anthocyanin biosynthesis in tobacco. It down-regulated the expression of *NbDFR* and *NbUFGT* when co-transfected with the CmMYB or SlAN1 (Fig. 8E and F). We compared the amino acid sequence of LiTTG1-1 with other TTG1s and found three mutation sites that may affect its function (Fig. S24A). At the M1 site, the five TTG1s are quite different, LiTTG1-1 has two arginine residues (RQHR), while the other TTG1s only have one arginine residue. At the M2 site, which is just before the first WD40 repeat domain, alanine (A) replaced proline (P). At the M3 site, aspartic acid (D) replaced glycine (G). We further compared the 3D structure of LiTTG1-1 (Fig. S24 B-G) with that of AtTTG1 and PgTTG1 and found that the M1 site forms a loop on the surface of the TTG1 proteins (Fig. S24 B and E), the M2 site forms an anti-parallel β-sheet in each of PgTTG1 and AtTTG1 and a big loop structure in LiTTG1-1 (Fig. S24 B and E), and the M3 site is similar among the TTG1 proteins. Therefore, the electric charge and structure of LiTTG1-1 are different from those of PgTTG1 and AtTTG1 which might affect the protein interaction. On the other hand, the amino acids of these three sites are conserved compared to the PgTTG1 which positively regulates anthocyanin biosynthesis. We hypothesized that LiTTG1-1 and LiTTG1-2 may competitively bind to MYB and bHLH, and thus the amount of active MBW complex gradually decreases with an increase in LiTTG1-1 expression. We are currently experimentally verifying this hypothesis.

We further investigated the possible substrate specificity of the biosynthesis pathway according to the components of anthocyanin in different colored petals. The amounts of the purple-based pigments (delphinidin, malvidin, and petunidin) are determined by the substrate specificity of LiDFR and the activity of the F3’5’H (Fig. 5). Famous commercial bluish flowers such as carnations, roses, and chrysanthemums were engineered by the heterologous expressions of F3’5’H and DFR [58]. DFR has been reported to be substrate-specific. For example, PhDFR (*Petuni*a × *hybrid* DFR) and FhDFR (*Freesia hybrid* DFR) preferentially use DHM over DHK as a substrate [59, 60], while the DFR of strawberry preferentially use DHK [61]. Furthermore, our findings suggest that the substrate preference of LiDFR determines the conversion rate of LiF3’H and LiF3’5’H. This may explain the low expression of *LiF3’5’H* despite the high product content of the LiF3’5’H branch of the pathway (Figs. 5 and 6). However, LiDFR itself does not explain the ratio discrimination of Mv3G and Dp3G between the DPB and PB. The methylation of the 3’ and 5’ hydroxyl groups of Dp3G is catalyzed by OMTs (Fig. S19), but no correlations were identified between LiOMT expression and Mv3G content; hence, the difference in ratios can be attributed to the activity of LiOMT. In *Paeonia spp.*, the activity of PsAOMT in the purple-flower plant is 60-fold higher than that of PtAOMT in the red-flowered plant [15]. The effects of genetic polymorphisms on the activities of LiDFR and LiOMT should be investigated to determine how they fine-tune the flower color in *L. indica.*

## Materials and methods

### Plant materials, de novo sequencing, and assembly

A local *L. indica* tree (NTU-1) growing in the Seyuan campus of Nantong University was selected for genome sequencing (E:120.623910, N:32.129528, Nantong, Jiangsu province). Young shoots and leaves following the cutting of branches were collected for genomic DNA extraction using a CTAB method. The PacBio, High-throughput Chromosome Conformation Capture (Hi-C), and Illumina technologies were combined to sequence the *L. indica* genome. For PacBio library construction, the genomic DNA was sheared to approximately 20 kb to prepare a SMRT library for the PacBio Sequel System. The Hi-C sequencing library was constructed according to the methods described by Niu et al. [62]. Sequencing was conducted on the Illumina PE150 platform using the paired-end method. Raw data were filtered using the HiCUP software [63]. The genome was assembled using Nextdenovo software (v2.3.1) with default parameter settings. The software included three modules: NextCorrect for the correction of errors in raw data, NextGraph for contig assembly, and Nextpolish for the correction of the errors in the assembled contigs (https://github.com/Nextomics/NextDenovo). The obtained contigs were further assembled at the chromosome level using ALLHIC software (v0.9.8) [64, 65].

### Genome quality assessment

The Benchmarking Universal Single-Copy Orthologs (BUSCO; http://busco.ezlab.org/) and Core Eukaryotic Genes Mapping Approach (CEGMA; http://korflab.ucdavis.edu/datasets/cegma/) were used to evaluate the completeness of the genome assembly. The short reads of Hi-C were aligned to the assembled genome to evaluate the mapping rate, and the sequencing depth using the BWA soft (http://bio-bwa.sourceforge.net/). The heterozygosity and accuracy of construction of the *L. indica* genome were evaluated using the samtools (http://samtools.sourceforge.net/) by SNP calling (Single-nucleotide polymorphism).

### Genome annotations

#### Repeat annotation

A combination of homology alignment and de novo search was used to identify the whole genome repeats in our annotation pipeline. Tandem repeats were extracted using TRF (http://tandem.bu.edu/trf/trf.html) by ab initio prediction [66]. Homolog prediction was performed using the Repbase database (http://www.girinst.org/repbase) and the RepeatMasker software (http://www.repeatmasker.org/) with default parameters to extract the repeat regions. In addition, ab initio prediction was used to build a database of de novo repetitive elements using LTR_FINDER (http://tlife.fudan.edu.cn/ltr_finder/) [67], LTR harvest (http://genometools.org/), LTR_retriever [68], RepeatScout (http://www.repeatmasker.org/), and RepeatModeler (http://www.repeatmasker.org/RepeatModeler.html) with default parameters. The raw transposable element (TE) library included all repeat sequences with lengths > 100 bp and gap ‘N’ less than 5%. Finally, a non-redundant library was generated by combining Repbase and our de novo TE library and was processed by RepeatMasker for DNA-level repeat identification. The LTR insertion time was conducted according to the methods described by Hu et al. [69] and the Wright Lab (https://github.com/SIWLab/Lab_Info/wiki/Ageing-LTR-insertions).

#### Protein coding sequence identification

Three approaches were used to predict the target genes: homolog-based, de novo-based, and RNA-Seq-based approaches. Sequences of homologous proteins from plant genomes, including *Eucalyptus grandis*, *Vitis vinifera*, *Populus trichocarpa*, *Arabidopsis thaliana*, and *Punica granatum* were downloaded from the Ensembl plant (http://plants.ensembl.org/index.html), phyotozome (https://phytozome-next.jgi.doe.gov/) [70], or NCBI databases. Protein sequences were aligned to the *L. indica* genome using TBASTN (v2.2.26; E-value ≤ 1e^− 5^) [68], and then the matching proteins were aligned to the homologous genome sequences for accurate spliced alignments with GeneWise software (https://www.ebi.ac.uk/Tools/psa/genewise, v2.4.1) that was used to predict the gene structure contained in each protein region. For gene prediction based on ab initio (*de novo*) methods we used the Augustus (http://augustusgobics.de/ v3.2.3), Geneid (http://genome.crg.es/software/geneid/ v1.4), GENESCAN (http://genes.mit.edu/GENSCAN/html, v1.0), GlimmerHMM (http://ccb.jhu.edu/software/glimmerhmm/, v3.04), and SNAP (http://korflab.ucdavis.edu/software.html) software in our automated pipeline. For RNA-Seq based gene prediction, different tissues (new roots, buds from cutting branches, young stem, leaves, flowers) were used for RNA-Seq, and RNA reads were assembled using the Trinity software (https://github.com/trinityrnaseq/trinityrnaseq/releases, v2.9.0) and aligned to genome sequences in fasta format using TopHat (http://ccb.jhu.edu/software/tophat/index.shtml, v2.0.11) to identify the exons region and splice positions. The alignment results were then used as inputs for StringTie (http://ccb.jhu.edu/software/stringtie/, v2.1.4) with default parameters to perform genome-based transcript assembly. The non-redundant gene set was generated by merging genes predicted by the three above-mentioned methods with EvidenceModeler (EVM) (http://evidencemodeler.sourceforge.net/, v1.1.1) using PASA (Program to Assemble Spliced Alignment, http://pasapipeline.github.io/, version 2.3.3). Gene functions were assigned using previously described methods [71, 72]. In brief, the datasets from databases such as Swissprot (http://web.expasy.org/docs/swiss-prot_guideline.html, version 05-24-2016), and NR database (E-value ≤ 1e^− 5^), InterPro (http://www.ebi.ac.uk/interpro/, version 32.0), Gene Ontology (GO, http://www.geneontology.org/page/go), Pfam (http://pfam.xfam.org/, version 27.0), and KEGG (Kyoto Encyclopedia of Genes and Genomes, http://www.kegg.jp/kegg/kegg1.html, release 53) were used to conduct gene function annotation.

#### ncRNA prediction

The rRNA sequences of *Populus trichocarpa* and *Arabidopsis thaliana* were used as references to predict that of *L. indica* using BLAT search. The tRNAs were predicted using the tRNAscan-SE program (http://lowelab.ucsc.edu/tRNAscan-SE/). Other ncRNAs, including miRNAs and snRNAs, were identified by searching against the Rfam database with default parameters using the infernal software (http://infernal.janelia.org/).

### Comparative genome analysis

Peptide sequences from *Arabidopsis thaliana* (TAIR10), *Acer truncatum* [73], *Cerasus serulata* [74], eucalyptus (*Eucalyptus grandis*) (https://phytozome-next.jgi.doe.gov/), *Ginkgo biloba* [75], *Oryza sativa*, *Populus trichocarpa*, *Prunus mume* [76], *Pyrus bretschneideri* [77], *Punica granatum* [33], *Rosa chinensis* [78], and Grape (*Vitis vinifera*) (https://phytozome-next.jgi.doe.gov/) were used to analyze genome evolution, including protein ortholog analysis, phylogeny construction, divergence time assessment, expansion and contraction of gene family, and chromosome collinearity. Peptides with lengths of no more than 50 amino acids were filtered, and only the longest predicted transcript per locus was retained for further analysis.

Orthologous relationships of the 13 species were inferred using all-against-all protein sequence similarity searches by OthoMCL (http://orthomcl.org/orthomcl/) (E value = 10^− 5^) [79], with the inflation value set at 1.5 and using other default parameters. The gene families of these 13 species, as well as those of a subset of five species (Arabidopsis, *E. grandis*, *P. granatum*, *P. trichocarpa*, and *L. indica)*, were also determined. Based on the results of the OthoMCL gene clustering, 327 single-copy gene families were aligned using the Muscle software (http://www.drive5.com/muscle/) [80], and the divergent and ambiguously aligned blocks of proteins were trimmed using Gblocks (http://molevol.cmima.csic.es/castresana/Gblocks.html) [81], and a Maximum Likelihood phylogenic tree was built using RAxML8 (http://sco.h-its.org/exelixis/software.html) [82]. The MCMC algorithm for Bayesian inference (Markov Chain Monte Carlo) was used to estimate the divergence times of species using the PAML software (http://abacus.gene.ucl.ac.uk/software/paml.html) [83]. The timescale of the plants was checked on the TimeTree website (http://www.timetree.org/) [84].

To identify the gene families undergoing expansion or contraction, the likelihood model in the software package Café(http://sourceforge.net/projects/cafehahnlab/) was implemented [85]. Both the phylogenetic tree topology and branch lengths were considered to infer the significance of a change in the gene family size in each branch. The orthologous or paralogous gene pairs were extracted from the syntenic blocks of *L. indica*, *Punica granatum, Eucalyptus grandis*, and *Vitis vinifera*. The 4DTv and the average synonymous substitutions per synonymous site (Ks) of the three *Myrtle* species were calculated via the methods reported by Qin et al. [29].

The synteny among *L. indica*, *Punica granatum*, *Eucalyptus grandis*, and *Vitis vinifera* was conducted using TBtools with default parameters (One Step for MCScanX, and Multiple Synteny Plot) [86].

### Transcriptome sequencing

*Lagerstroemia indica* cv. Ebony Embers series: “pure white” (different parts of YS, flower) was used for transcriptome sequencing. The transcriptomic data of PB and DPB were obtained from our previous report [3].

### Identification of gene families involved in flavonoid/anthocyanin pathway

The enzyme-coding genes involved in the PAL pathway (PAL, C4H, 4CL), flavonoid (including anthocyanin) biosynthesis (CHS, CHI, FNS, F3H, F3ʹH, F3ʹ5ʹH, FLS, DFR, LDOX/ANS), and decoration of flavonoid (UFO/CGT, UFOMT, GH1-GT), as well as the MYB gene family and the the LiTTG1 gene, were identified in the reference genome of *L. indica.* Homologous genes were screened using the methods described in our previous study [85]. In brief, HMM (http://pfam.xfam.org/) and batch CD searches (https://www.ncbi.nlm.nih.gov/Structure/bwrpsb/bwrpsb.cgi) were conducted, sequences were aligned using ClustalW software (http://www.clustal.org/omega/), and very long or short members were removed. Tbtools [86] and MEGAX (https://www.megasoftware.net/) were further used to construct the phylogenetic tree and the gene structure.

### UPLC-MS/MS to detect flavonoid/anthocyanin

Petals of fully blooming flowers were collected, frozen in liquid nitrogen, and stored at -80. Before detection, the samples were freeze-dried and ground into powder and flavonoids/anthocyanins were extracted and detected as previously described [87].

### Determination of the intracellular pH of petals and observation of epidermal cells by scanning electron microscopy

The intracellular pH in the petals of three different flower types was measured and the shapes of epidermal cells were observed using a JSM-6510 scanning electron microscope (Japan, Tokyo) as previously described [88].

### RNA extraction, first-strand cDNA synthesis, and qPCR

RNA extraction and first-strand cDNA synthesis were performed using kits obtained from Tiangen (DP441, Beijign) and TaKaRa (RR037A, Beijing) according to the manufacturer’s instructions. The qPCR mix was prepared using a SYBR Green PCR kit (CW2601H) (Kangwei, Beijing). Relative expression levels were calculated using the 2^−△△Ct^ method with Excel software and normalized using the internal and sample controls.

### *LiTTG1-1* gene cloning and plasmid construction

The primers specific to *LiTTG1-1* genes were designed according to the genomic data and the transcriptome data obtained in this study (Table S18). Full-length *LiTTG1-1* was cloned from the red flower cDNA and sub-cloned into the plant expression vector pWM101.

### Transient transformation of tobacco leaves

Experiments were conducted by the agrobacterium-mediated transient expression in tobacco (*Nicotiana benthamiana*) according to a previously described method. In brief, the pWM101-LiTTG1-1 plasmid was transformed into agrobacterium GV3101 and infiltrated into 5-week-old leaves. Agrobacterium containing the empty vector pWM101 was used as the negative control and that containing CmMYB6 from chrysanthemum or SlAN1 (bHLH) from tomato as the positive control [40]. Samples were collected 72 h after infiltration, and quantitative reverse transcript PCR (qRT-PCR) was conducted according to the manufacturer’s instructions. Photos of tobacco leaves and total anthocyanin [3] were detected after infiltration of 9 days.

### Over-expression of LiTTG1-1 in *Arabidopsis* and the phenotype observation

The *35 S::LiTTG1-1* was over-expressed into *Arabidopsis thaliana* (Col-0) through agrobacterium-mediated floral dip transformation. Positive lines were screened by 1/2 MS with 2% sucrose and hygromycin (20 ug/ L) and the genotypes were identified by genomic PCR [49]. To induce anthocyanin accumulation during the germination, seeds were grown on 1/2 MS with or without 3% sucrose. Phenotypes of seeds and YS were observed by a Lecia S8AP0 dissecting microscope and photos were taken using a Leica DFC 295 CDD camera and processed using the Leica QWin V3 software.

### Data analysis and graph drawing

Heatmaps, Venn diagrams, genome circle graphs, and genome collinearity diagrams were drawn using TBtools. Column figures were drawn using GraphPad Prism 9.0.0, and differences within the data were analyzed using one-way ANOVA by GraphPad software (https://www.graphpad.com/).

### Electronic supplementary material

Below is the link to the electronic supplementary material.

**Supplementary Material 1:** Supplementary figures

**Supplementary Material 2:** Supplementary tables

## Data Availability

The datasets generated and/or analysed during the current study are available in the China National GeneBank DataBase (CNGBdb) repository with accession number CNP0003018 (genomic data), CNP0001693, CNP0003990 and CNP0003991.
