# Supplementary material for: Genomic and transcriptomic studies on flavonoid biosynthesis in Lagerstroemia indica
Source: BMC Plant Biol. 2024 Mar 5;24:171. doi: 10.1186/s12870-024-04776-4 (PMC10913235; doi:10.1186/s12870-024-04776-4)
Supplement: Supplementary file 1 — Supplementary Material 1: Supplementary figures [file 12870_2024_4776_MOESM1_ESM.docx]

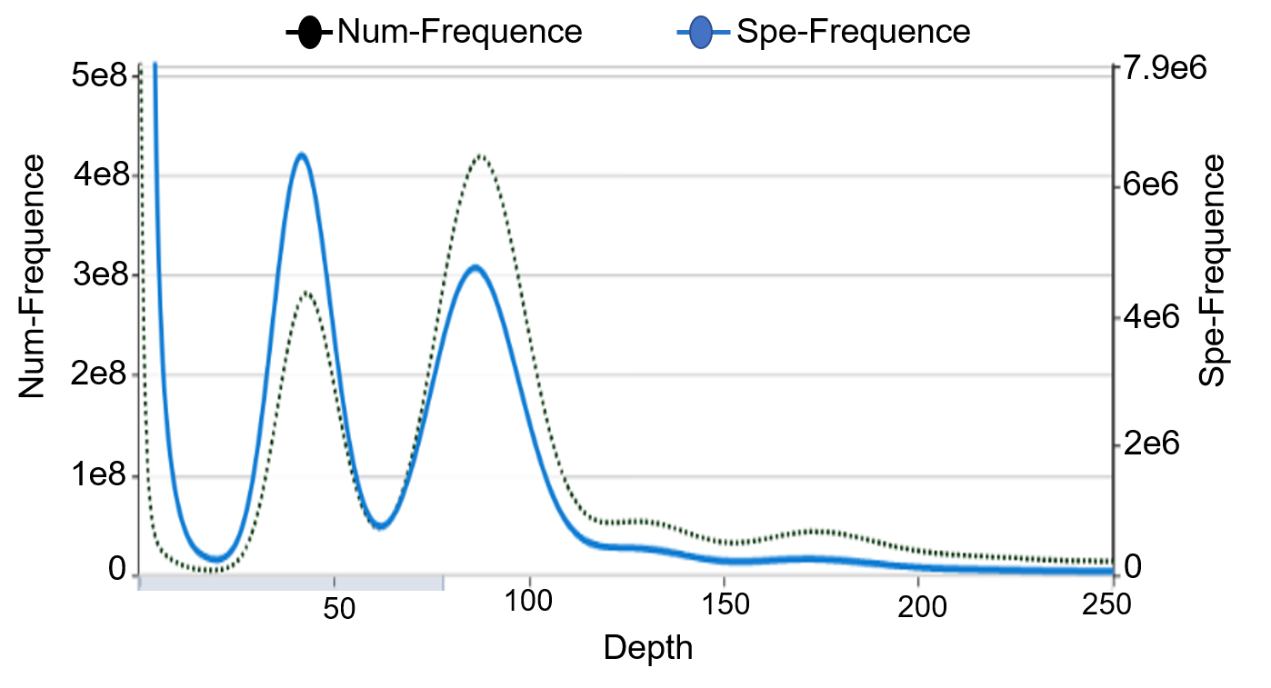


Supplementary Fig. S1 Estimate the genome size of *Lagerstroemia indica* by 17-mer based methods*.*


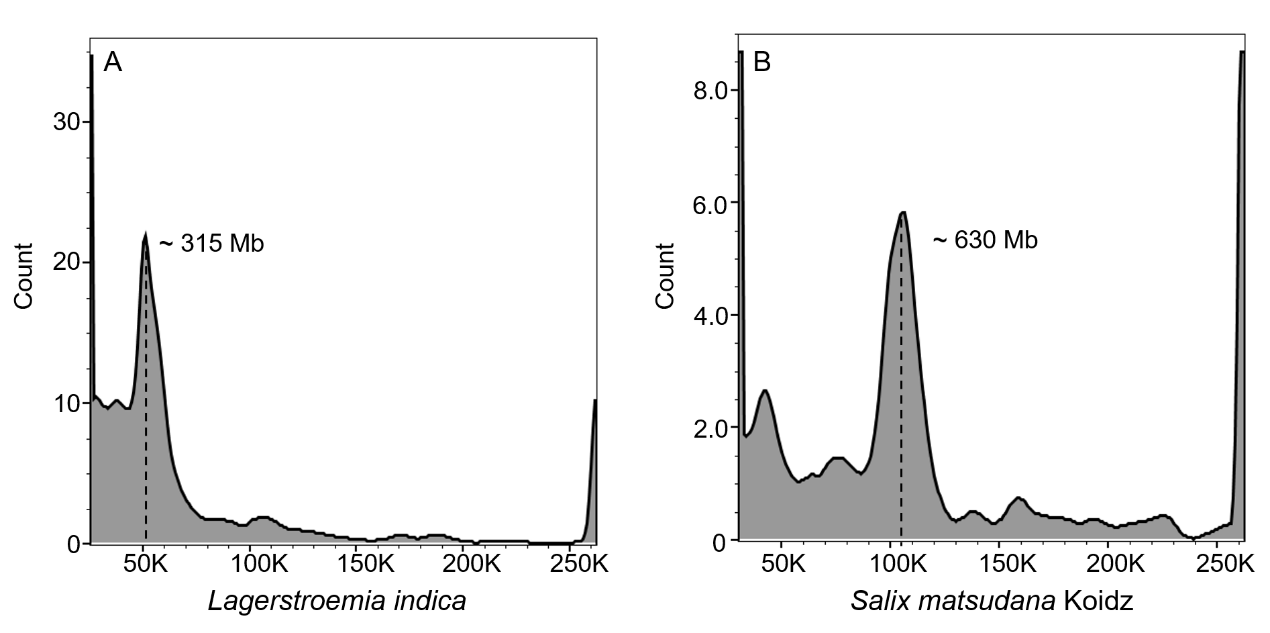


Supplementary Fig. S2 Detection genome size of *L. indic*a using flow cytometry. The genome of *L. indica* (A) was estimated by using our previously reported genome *Salix matsudana* Koidz (B) as controls.


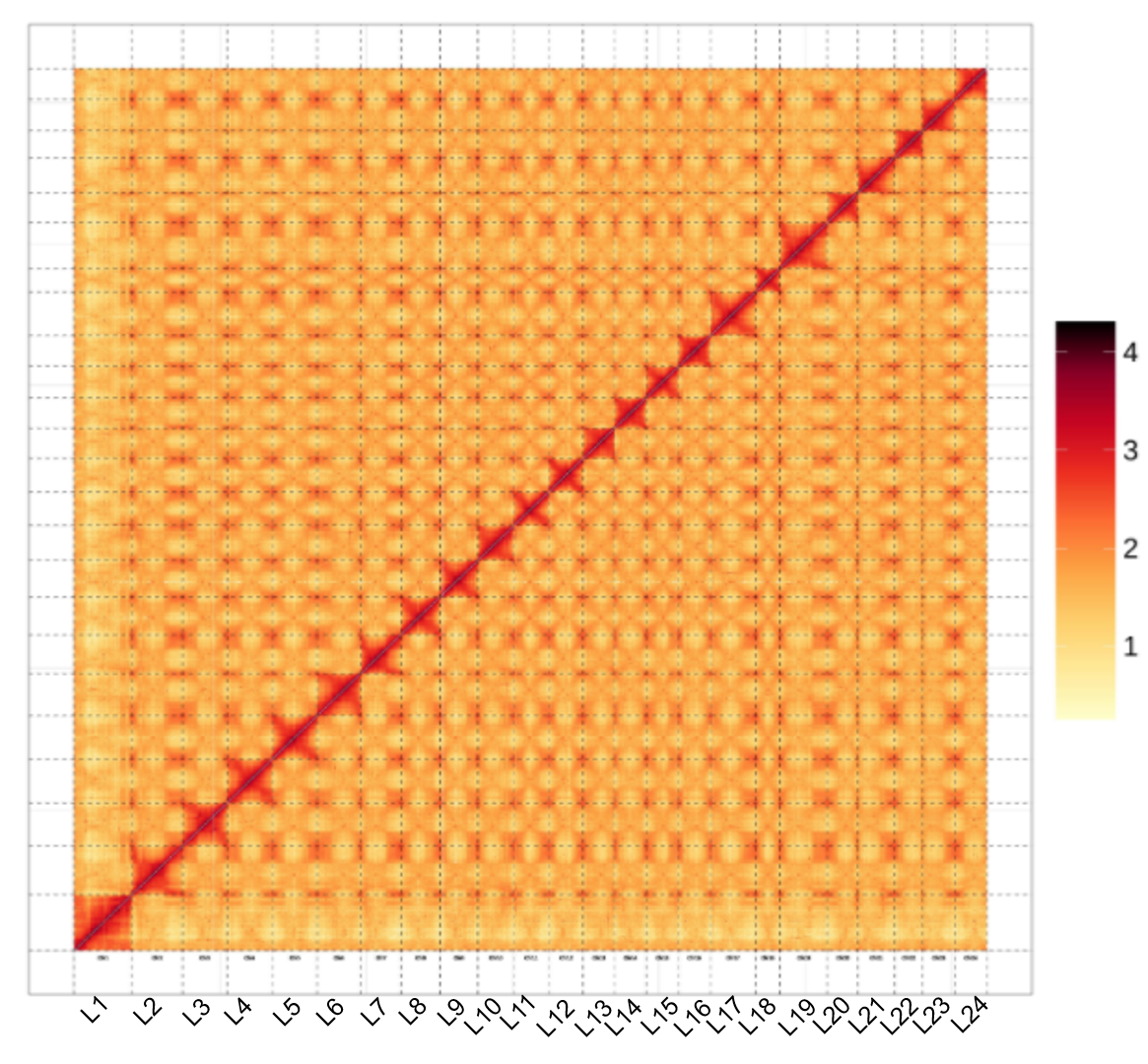


Supplementary Fig. S3 Chromosomal Hi-C contact map of the *L. indica* genome. The intensity of each pixel represents the count of Hi-C links between 500 kb windows on chromosomes on a logarithmic scale. The strongest and weakest contact is shown in red and yellow, respectively.


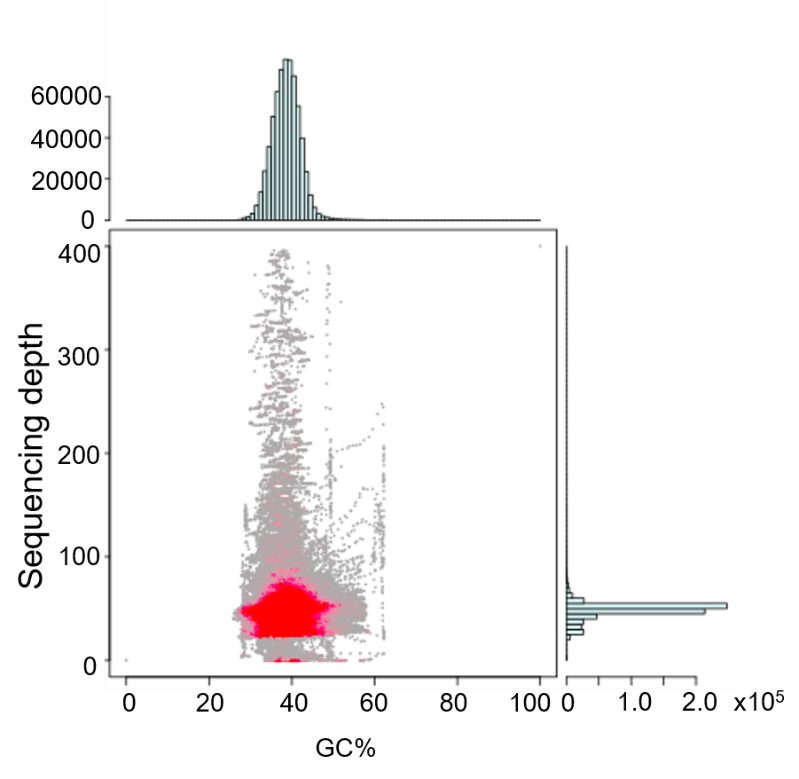


Supplementary Fig. S4 The distribution of GC content in *L. indica* genome. The GC content is about 38.64% of the genome. The dots distributed in the scatter diagram indicated there was no pollution of the genome.


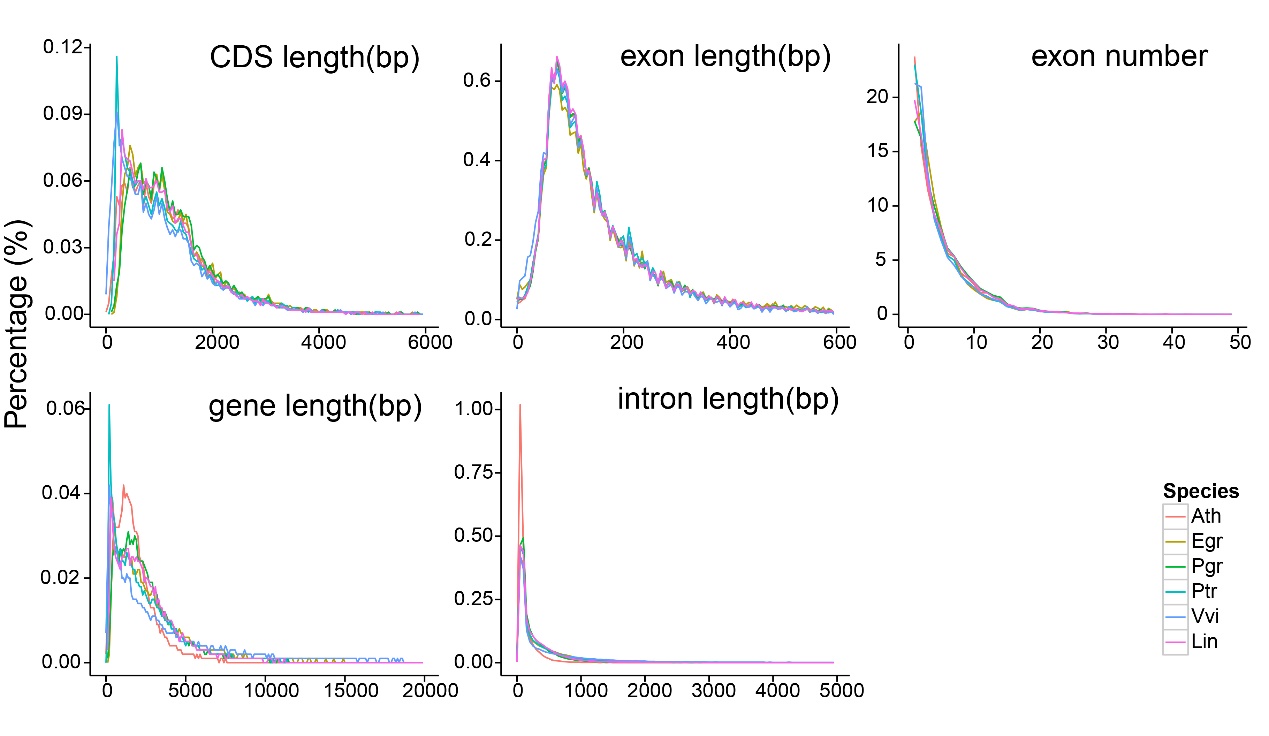


Supplementary Fig. S5 The gene characterize of the six genomes. Ath, *Arabidopsis thilian*a; Egr, *Eucalyptus grandis*; Pgr, *Punica granatum*; Vvi, *Vitis vinifera*; Lin, *Lagerstroemia indica.*


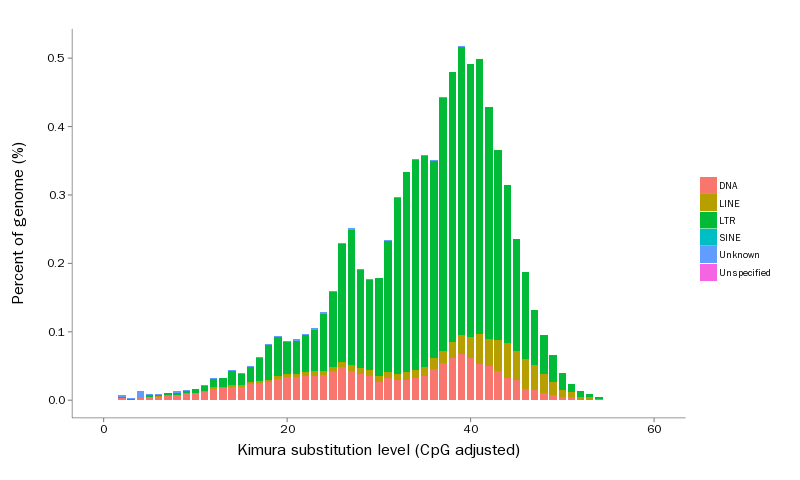


Supplementary Fig. S6 Distribution of the transposons. LINE: long interspersed nuclear element; SINE: short interspersed nuclear elements; LTR: long terminal retrotransposons.

*
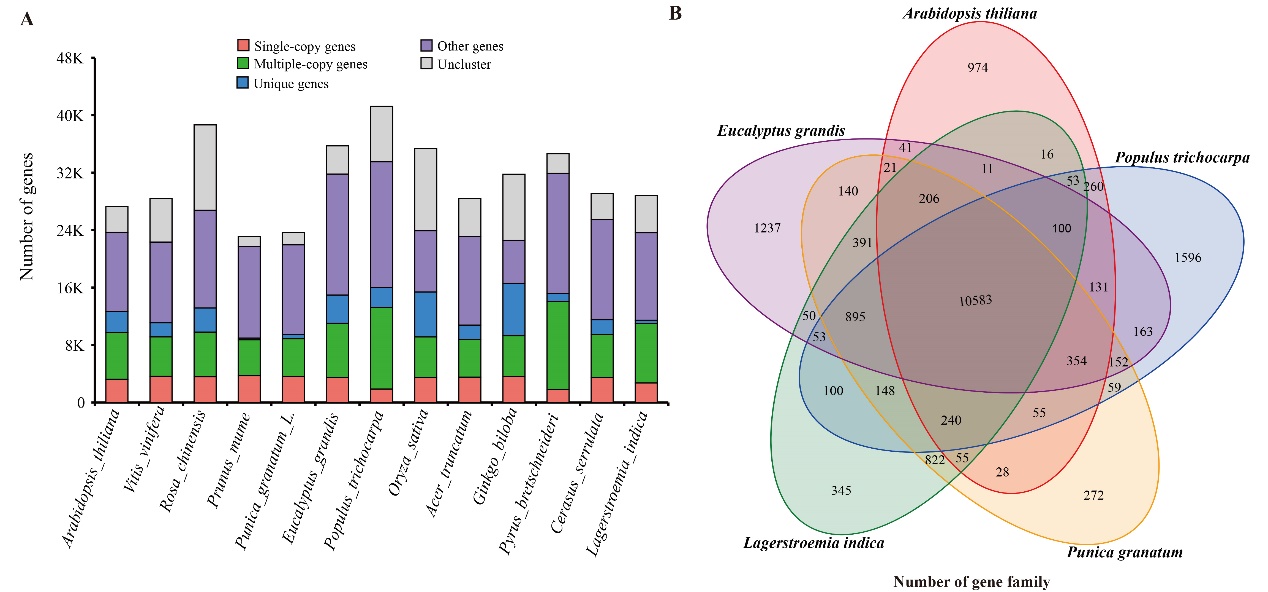
*

Supplementary Fig. S7 Comon and unique gene families among five species.


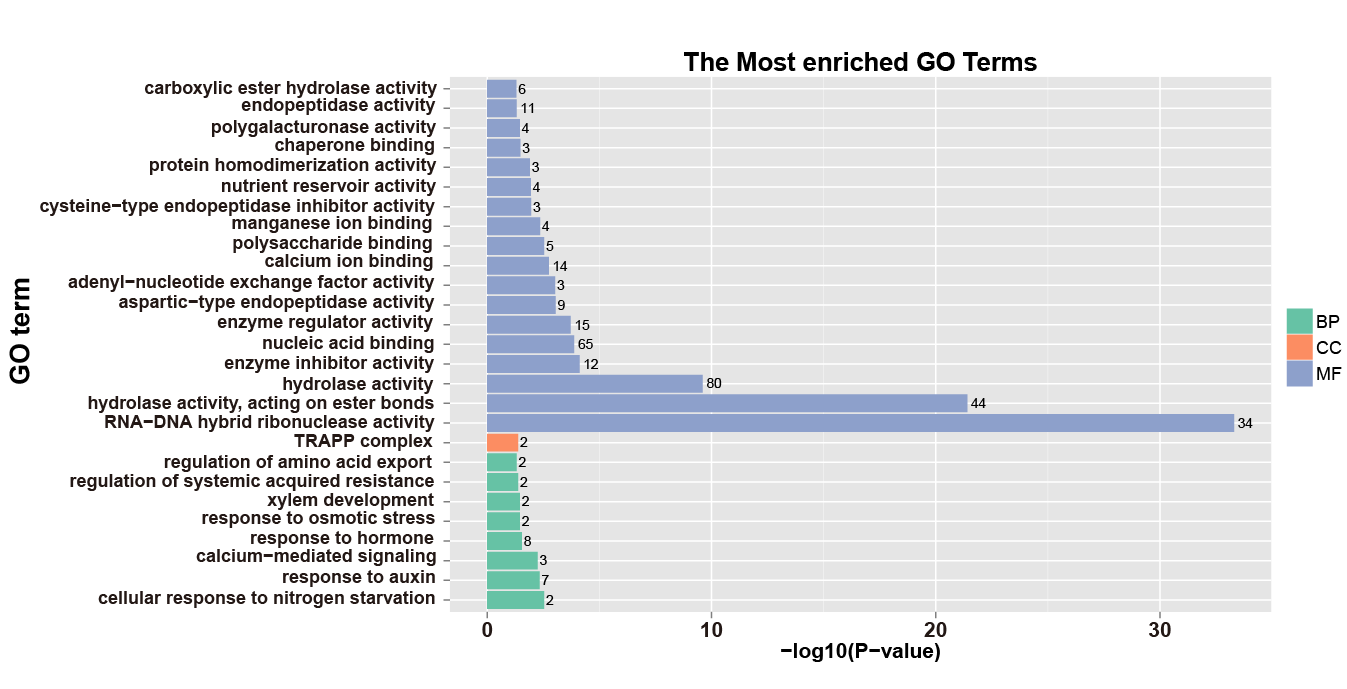


Supplementary Fig. S8 GO enrichment of *L. indica* special genes families. BP, Biological Process; CC, Cell component; MF, molecular function.


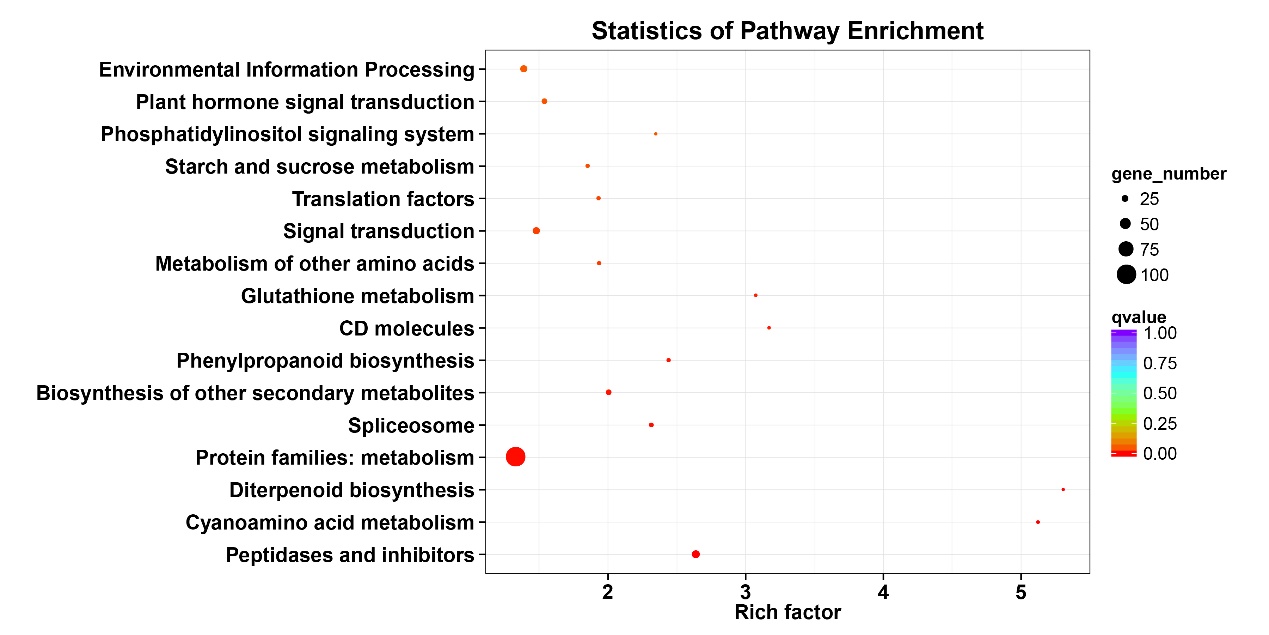


Supplementary Fig. S9 KEGG enrichment of *L. indica* unique genes.


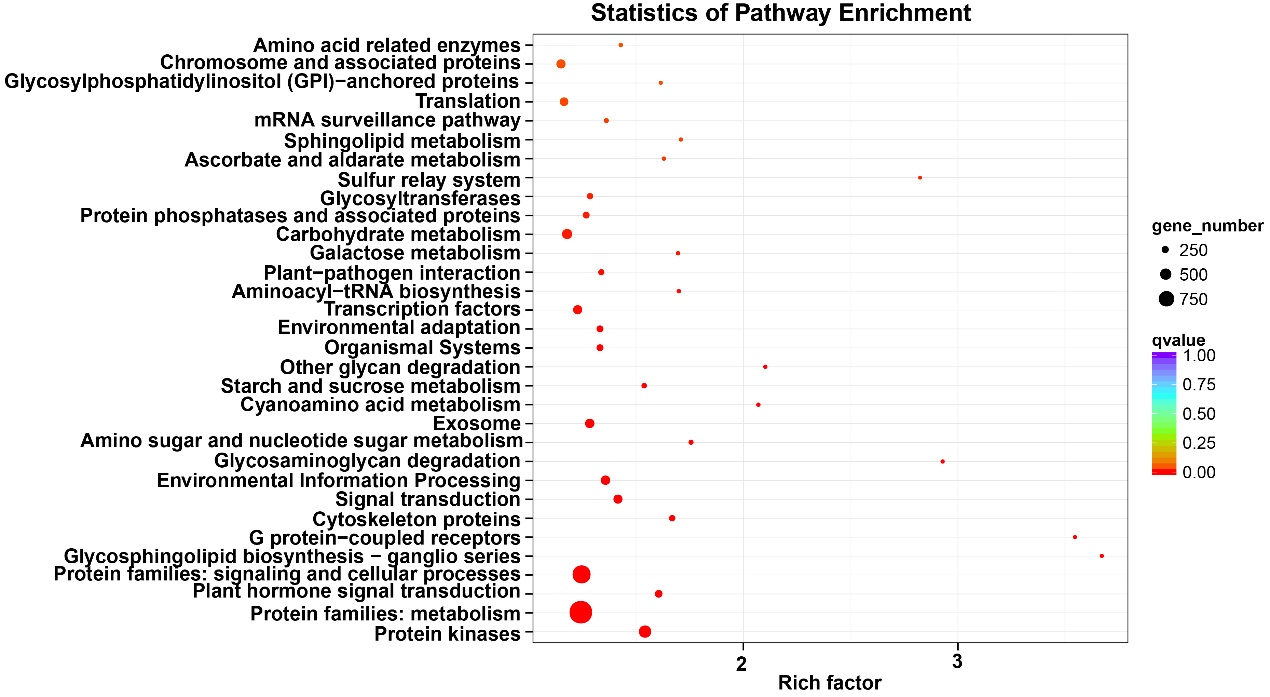


Supplementary Fig. S10 KEGG enrichment of *L. indica* expansion genes.


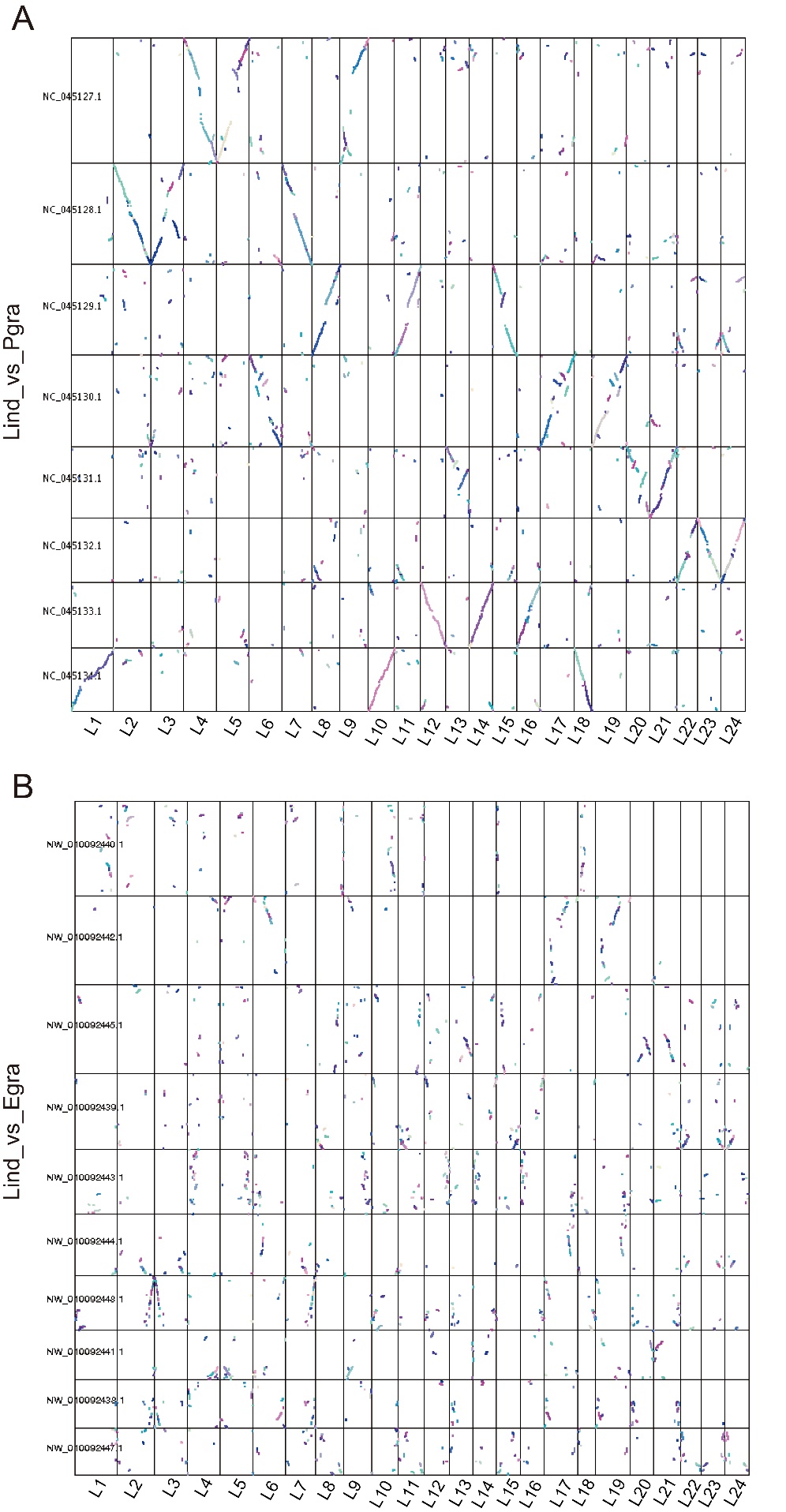


Supplementary Fig. S11 Chromosome synteny relationship interspecies. *L. indica* and *P. granatum* (Lind_vs_Pgra) (A). *L. indica* and *E. grandis* *(Lind*_vs_*Egra*) (B).


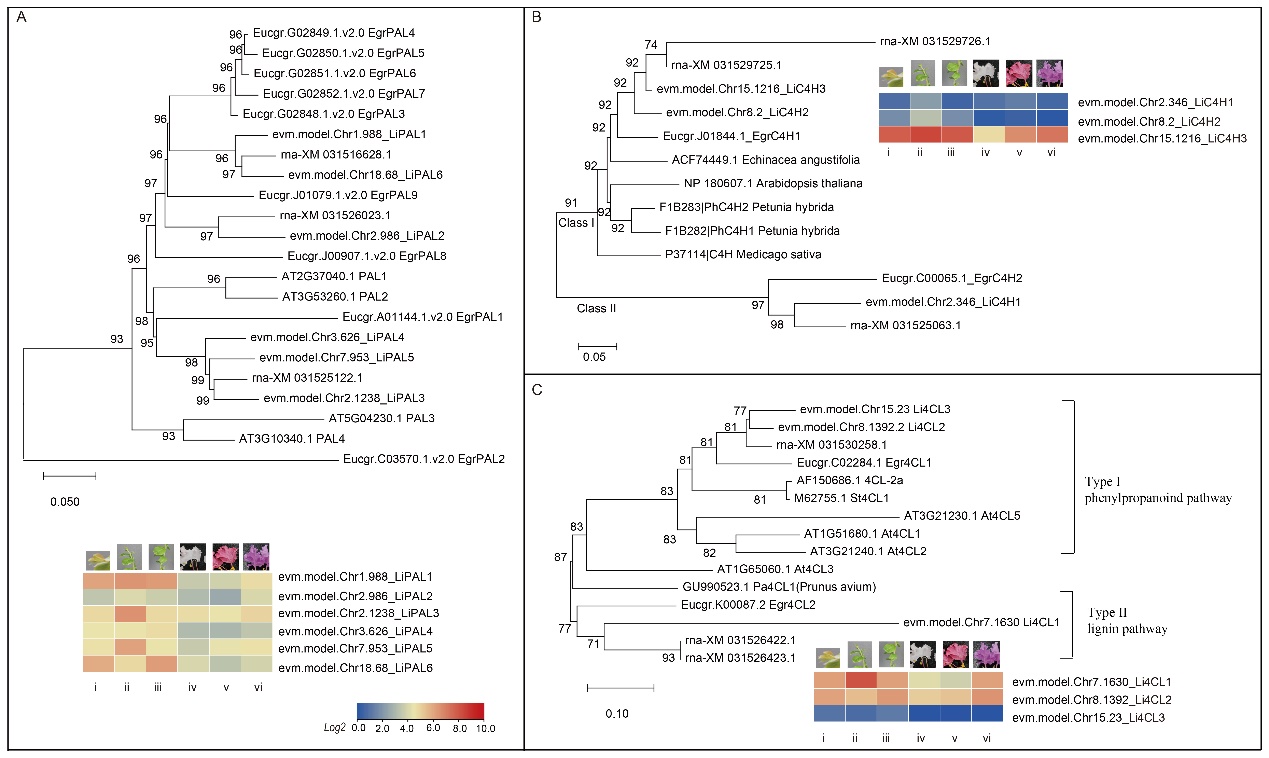


Supplementary Fig. S12 Phylogenic tree and expression of three phenylpropanoid pathway genes. PAL(A), C4H (B) and 4CL(C). The tip (i), up (ii) and bottom (iii) parts of the young shoots (*L. indica* var Ebony Embers “pure white”); Three local varieties with different petals color: white (iv), deep purplish pink (DPB, 68A) (v), light purple (PB, 75A) (vi). *Eucgr* (*Egr*), *Eucalyptus grandis*; *AT*, *Arabidopsis thaliana*; *Li*, *Lagerstroemia indica; rna-XM*, *Punica granatum*; *St*, *Solanum tuberosum.* Abbreviation of gene name are depicted in Table S13.


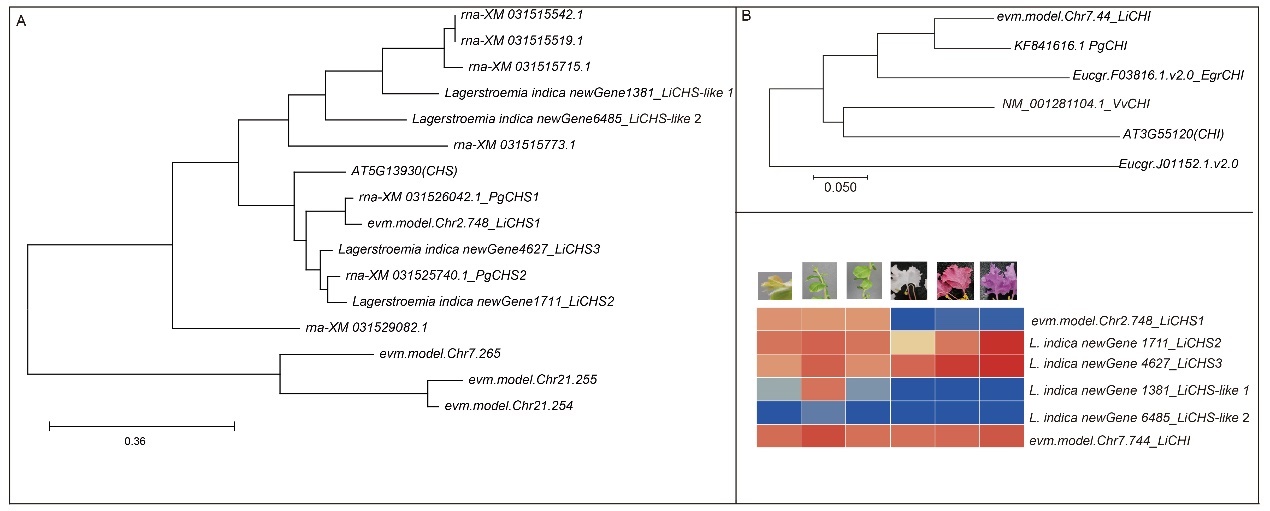


Supplementary Fig. S13 Phylogenetic tree and expression of CHS and CHI. A, CHS genes from *Largestromia indica, Punica granatum* and *Arabidopsis.* B, CHI*.* Eucgr (Egr), *Eucalyptus grandis*; AT, *Arabidopsis thaliana*; Li, *Lagerstroemia indica*; rna-XM, *Punica granatum*; Vv*, Vitis vinifere.* Plant materials used in this figure and the hot of the expression level are same as that of Fig. S12, abbreviation of gene name are depicted in Table S13.


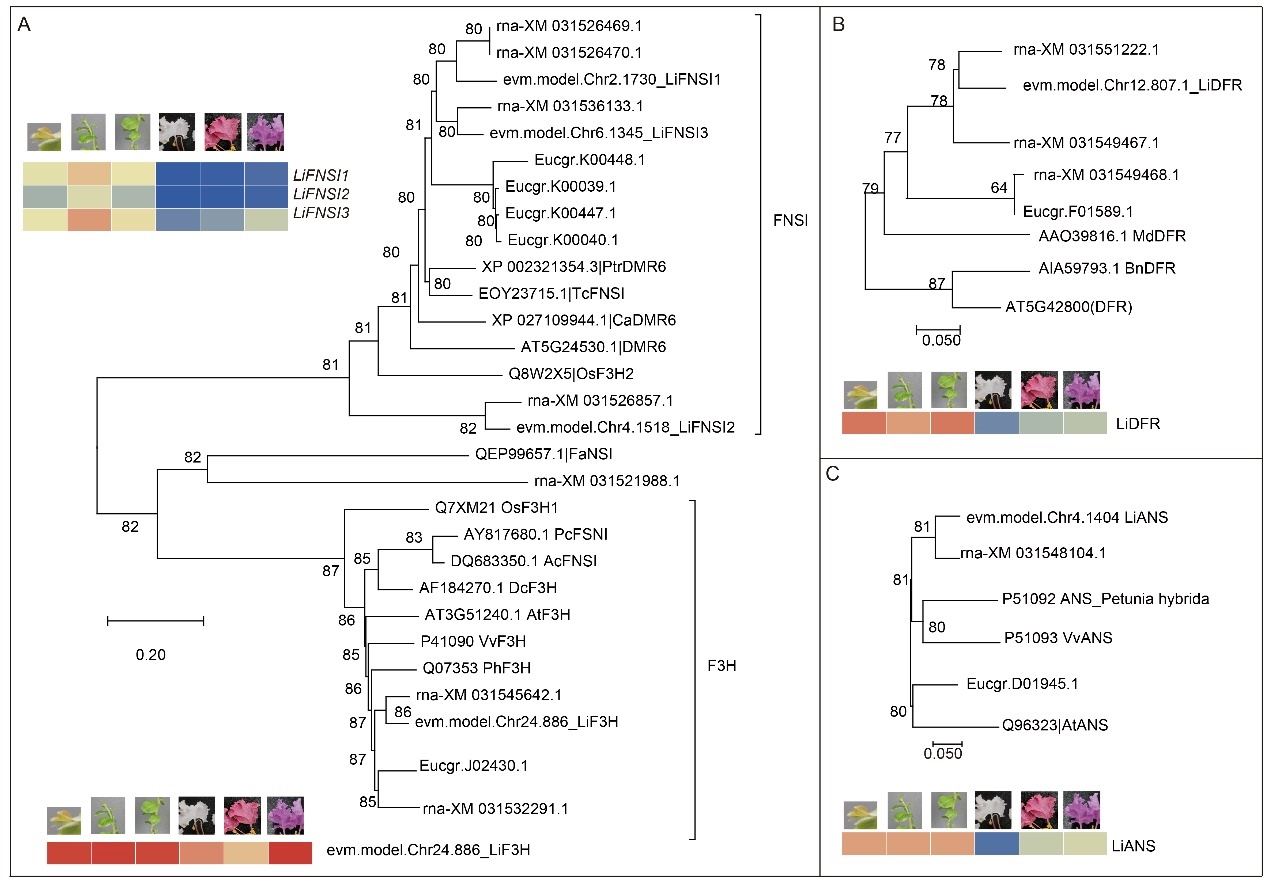


Supplementary Fig. S14 Phylogenetic tree of FNSI, F3H, DFR and ANS. A, FNSI and F3H. B, DFR. C, ANS. Ac*,* *Aethusa cynapium*; Bn, *Brassica napus*; *AT*, *Arabidopsis thaliana;* Ca, *Coffea arabica*; Dc, *Daucus carota*; Eucgr, *Eucalyptus grandis*; Md, *Malus domestica*. Tc, *Theobroma cacao*; rna-XM, *Punica granatum*; Pa, *Plagiochasma appendiculatum*; Ph, *Petunia hybrid*; Os, *Oriyza sativa*; Vv, *Vitis vinifere*. Tissues and the abbreviations are illustrated in Fig. S12, abbreviation of gene name is listed in Table S13.


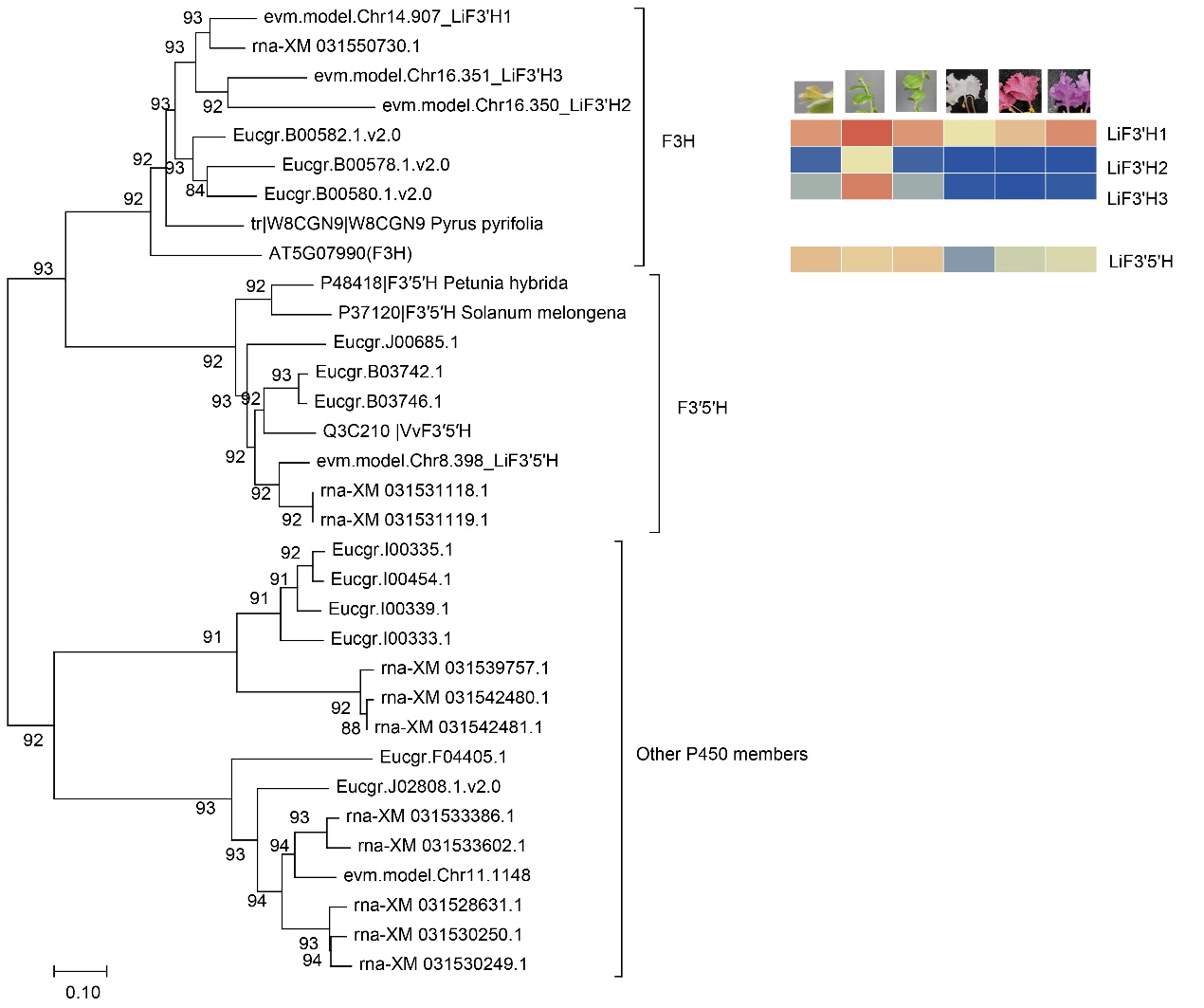


Supplementary Fig. S15 Phylogenetic tree of F3′H and F3′5′H. Tissues and the abbreviations are illustrated in Fig. S12, abbreviation of gene name is listed in Table S13. UniProtKB accession numbers of other species except that of *E. grandis*, *V. vinifere*, *P. granatum* are listed in the figure.


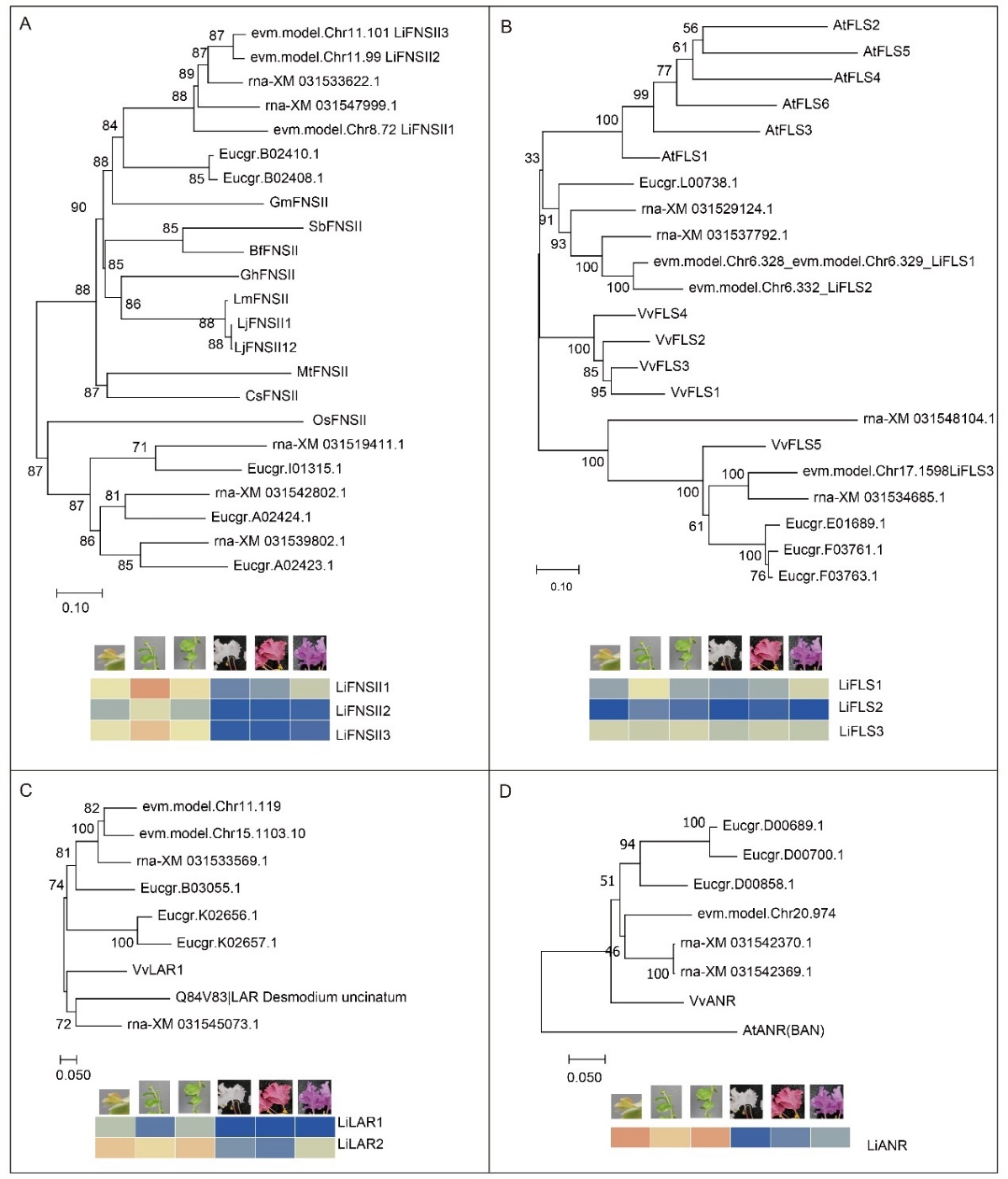


Fig. Supplementary Fig. S16 Phylogenetic trees of genes involved in branch pathway of flavone, flavonol and flavanol. A, FNS II. CsFNSII*,* *Camellia sinensis* (ACH99109.1); GhFNSII, *Gerbera hybrid*(C93B2); GmFNSII, *Glycine max* (E9KBR8); GhFNSII, *Gerbera hybrid* (Q9XGT9) LjFNSII, *Lonicera japonica* (AMQ91111.1, AMQ91109.1); LmFNSII, *Lonicera macranthoides* (AMQ91113.1); MtFNSII, *Medicago truncatula* (Q0ZM38); OsFNSII, *Oryza sativa* (Q0JFI2); PfFNSII, *Perilla frutescens* (BAB59004.1); SbFNSII, *Scutellaria baicalensis* (AMW91729.1). B, FLS. AtFLSs (AT5G08640, AT5G63580, AT5G63590, AT5G63595, AT5G63600, and AT5G43935); VvFLSs (Q2PHK5, Q2PHK4, Q2PGC6, Q2PHJ7 and Q2PGC5). C, LAR. *DuLAR*, *Desmodium uncinatum* (Q84V83); VvLAR1, (Q4W2K4). D, ANR. VvANR*,* *Vitis vinifera* (D7U6G6); AtANR (BAN), Q9SEV0. At, *Arabidopsis thaliana*; Vv, *Vitis vinifera.* Tissues are illustrated in Fig. S12, abbreviation of gene name is listed in Table S13.


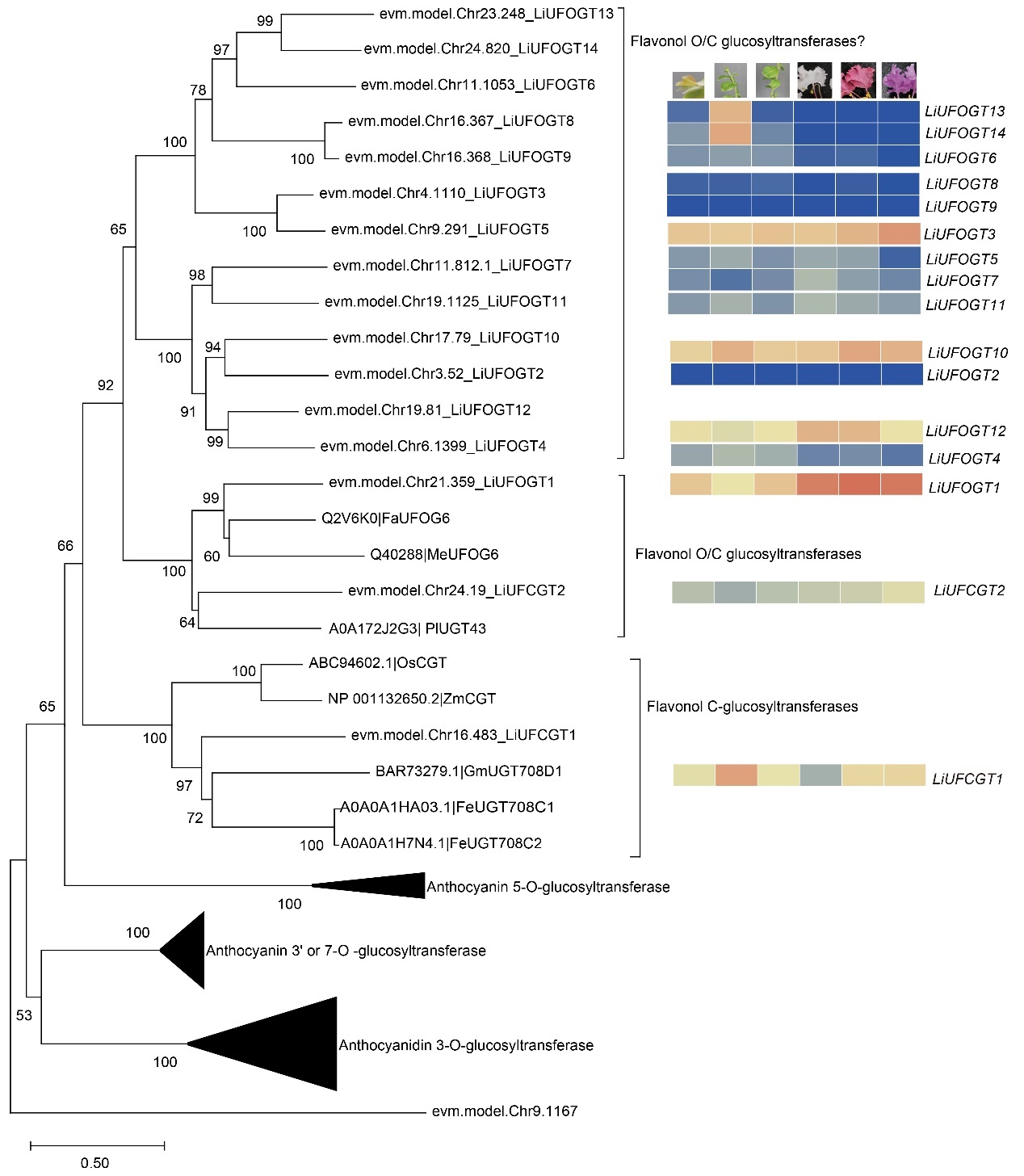

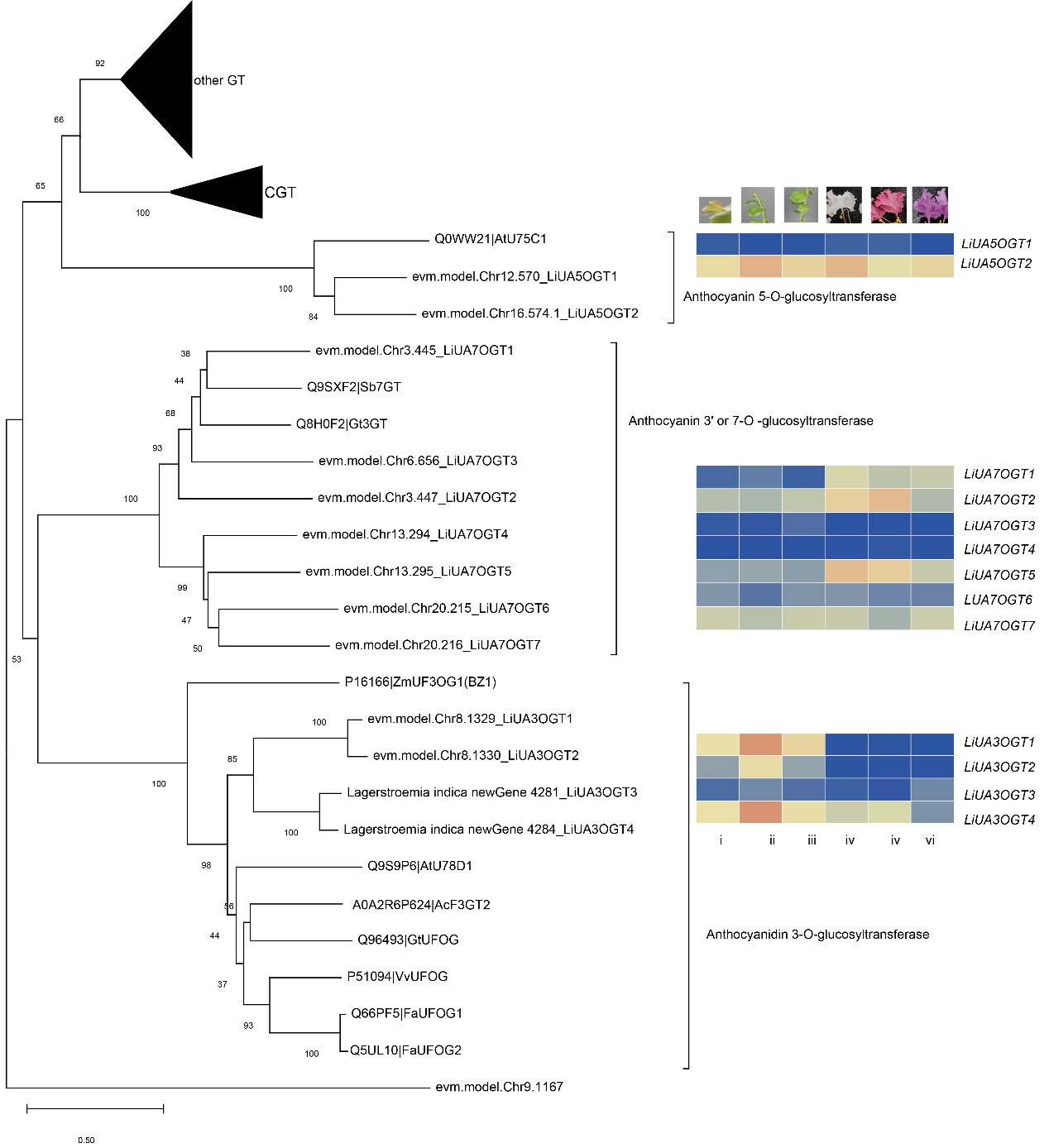
Supplementary Fig. S17 Phylogenetic tree of UDP-glucose O-glucosyltransferases (GT).

At, *Arabidopsis thaliana*; Ac, *Actinidia chinensis* var. *chinensis*; Fa, *Fragaria ananassa*; Fe, *Fagopyrum esculentum*; Gm, *Glycine max;* Gt, *Gentiana triflora*; Me, *Manihot esculenta*; Os, *Oryza staiva*; Pl, *Pueraria montana var. lobata*; Pm, *Pueraria montana*; Sb, *Scutellaria baicalensis*; Vv, *Vitis vinifera*; Zm, *Zea mays*. Numbers are GenBank and UniProtKB accession number. Tissues are illustrated in Fig. S12, the abbreviation of gene name are listed on Table S14.


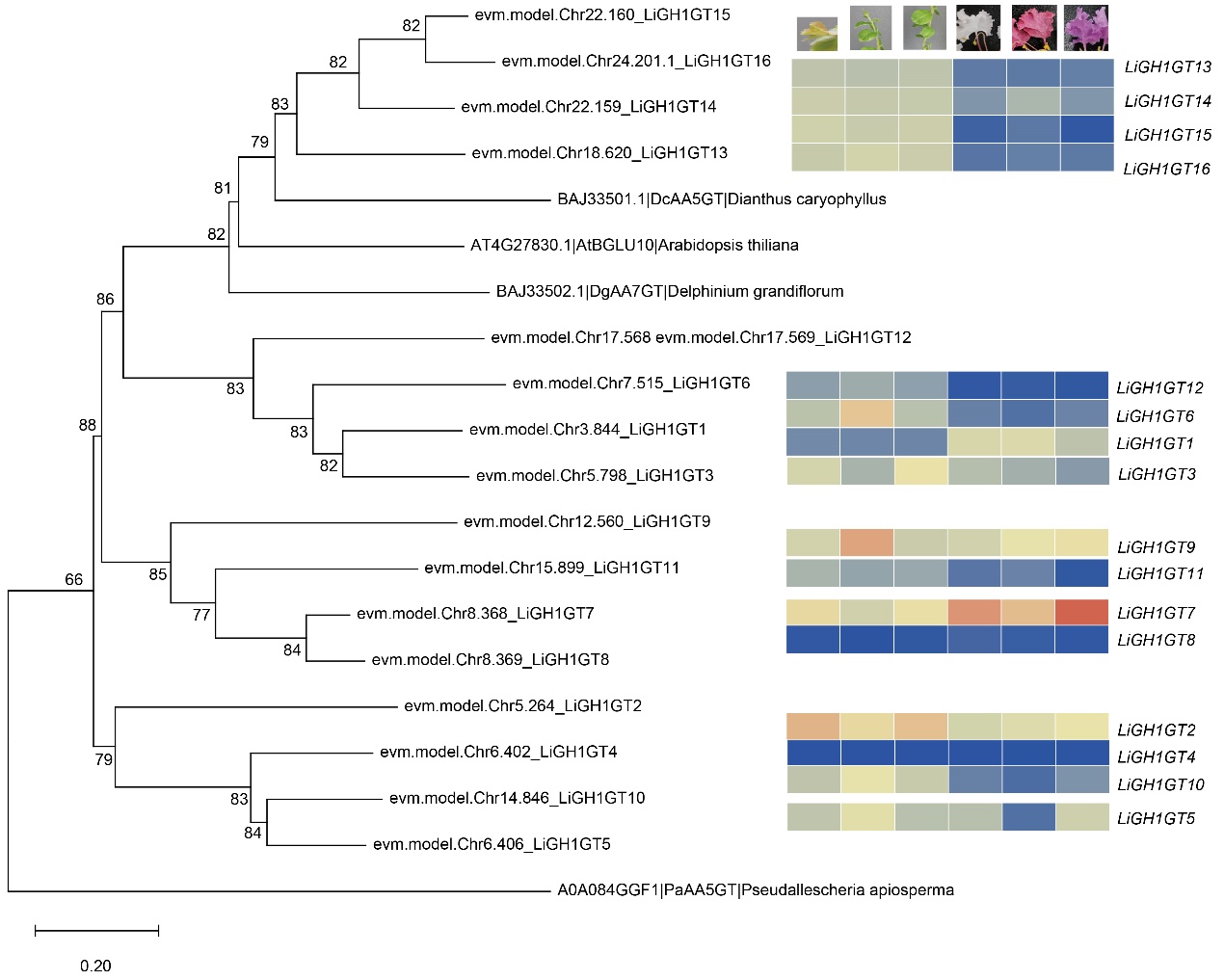


Supplementary Fig. S18 Phylogenetic tree and expression of Glycoside hydrolase family 1 glucosyltransferase (GH1-GT). Numbers are GenBank and UniProtKB accession number or genome ID. Species are shown on tree. Tissues are illustrated in Fig. S12, the abbreviation of gene name are listed on Table S14.


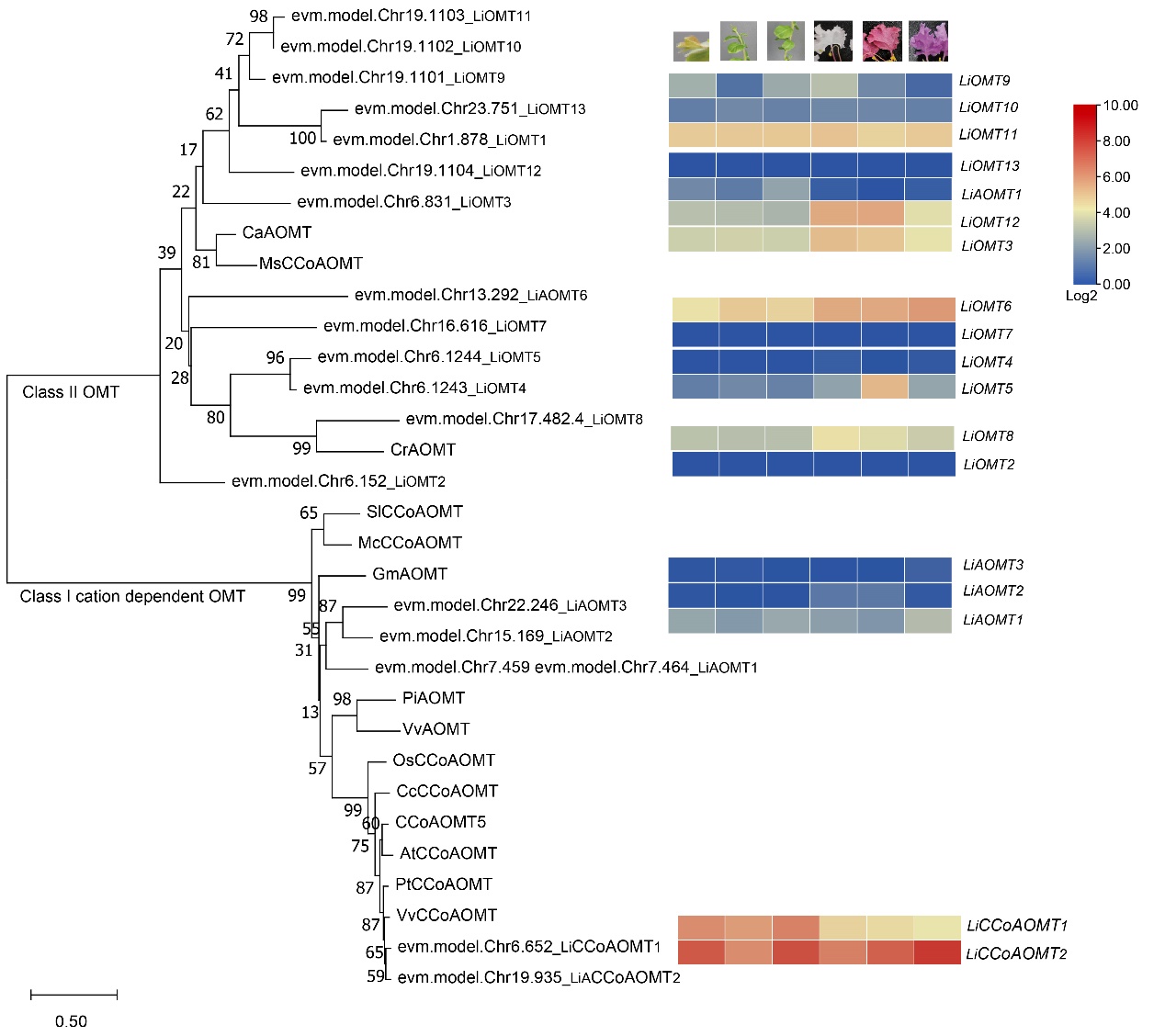


Supplementary Fig. S19 Phylogenetic tree and expression S-adenosyl-L-methionine (SAM)- dependent O-methyltransferases. AtCCoAOMT, *Arabidopsis thiliana* (AT4g34050); CaAOMT, *Chrysosplenium americanum* (U16794.1); CcCCoAOMT, Cryophytum crystallinum (O65162); CrAOMT, *Catharanthus roseus* (AY127568.1); GmAOMT, *Glycine max* (ADX43927.1); MsCCoAOMT, *Medicago sativa* (P28002); McCCoAOMT, *Mesembryanthemum crystallinum* (AY145521.1); NtCCoAOMT5, *Nicotiana tabacum* (O04899); OsCCoAOMT, *Oryza sativa* Japonica Group (BAA78733.1); PiAOMT, *Petunia integrifolia* (AIE77048.1); PtCCOAOMT, *Populus trichocarpa* (CAA12198.1); SlCCoAOMT, *Stellaria longipes* (L22203.1); VvAOMT, *Vitis-vinifera* (ACO52469.1); VvCCoAOMT, *Vitis vinifera* (Q43237.1). Numers are GenBank ID or UniProtKB. CCoAOMT, Caffeoyl-CoA O-methyltransferase; AOMT, anthocyanin O- methyltransferase. Plant materials used in this figure and the hot of the expression level are same as that of Fig. S12.


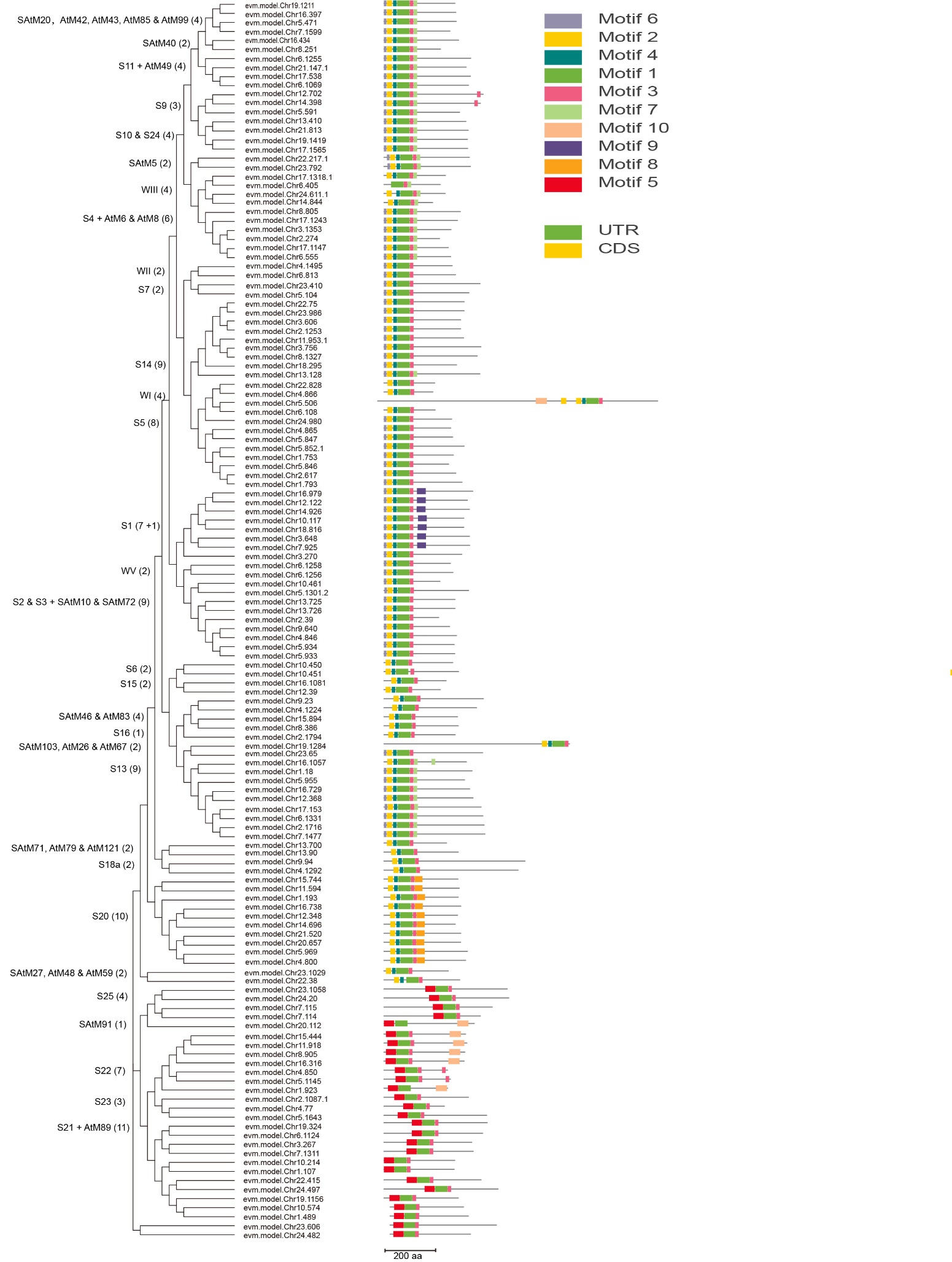


Supplementary Fig. S20 Characteristics of LiR2R3 MYB. Left, NJ phylogenic tree of LiR2R3 MYB; Right, conserved motifs of the LiR2R3 MYB protein. Motifs were showed by different colored box, the other sequences by gray line.


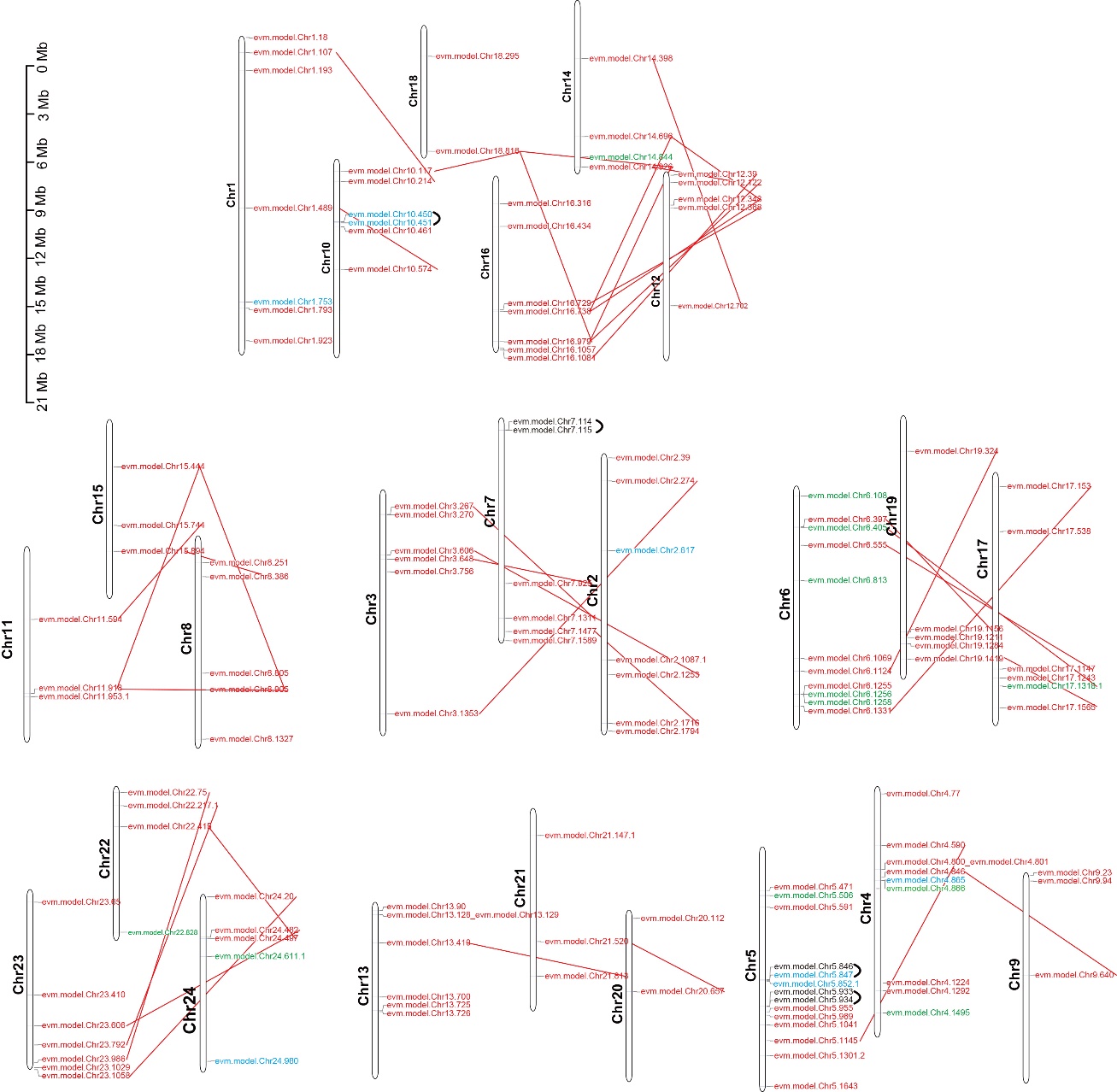


Supplementary Fig. S21 Chromosomes location of R2R3 LiMYBs. Tandem repeats show by black lines, fragment duplication gene in red lines；members belong to subgroup 5 (SG5) and SG6 in blue, members belong to woody-preferential subgroup WPSI-V are in green.
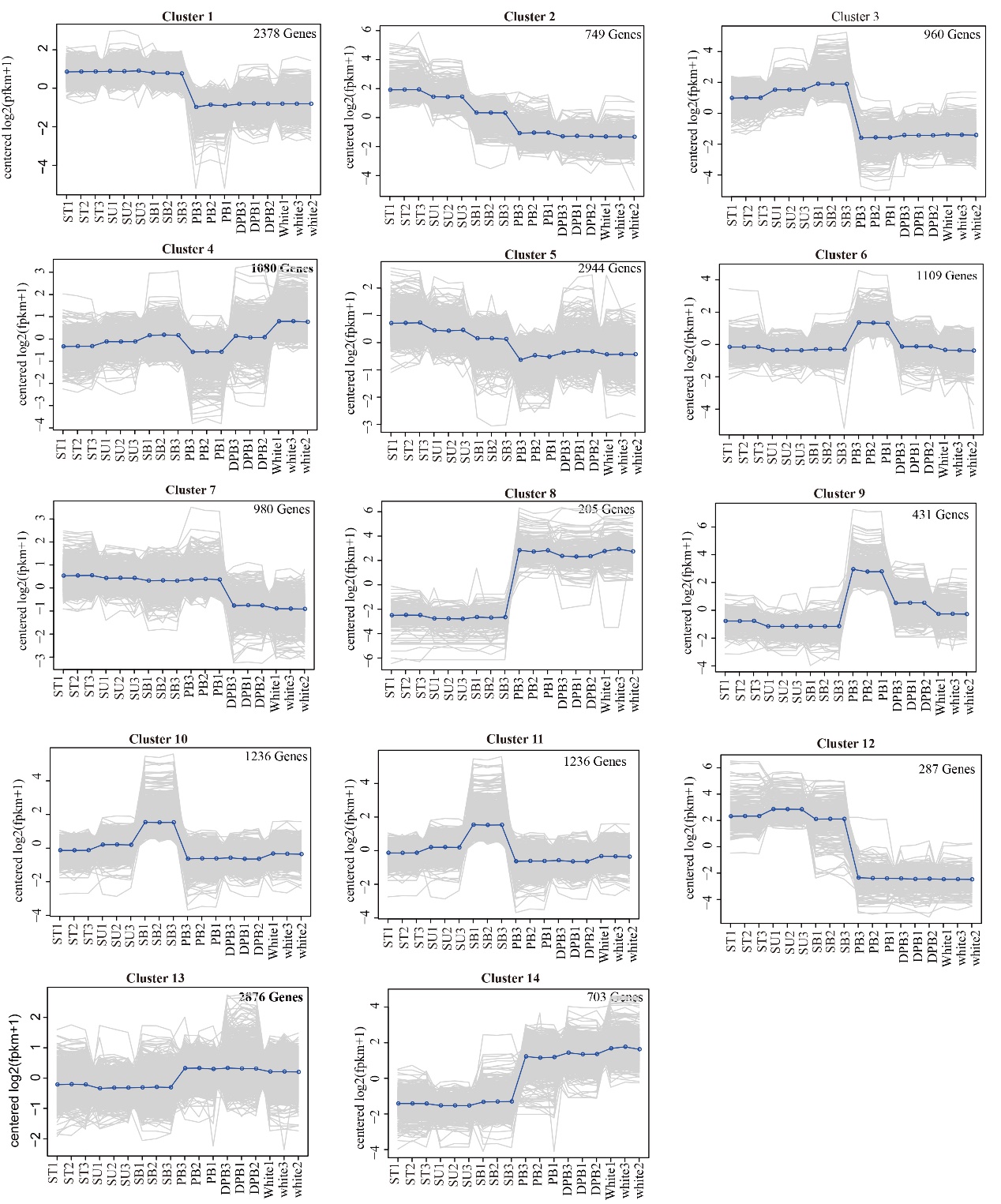


Supplementary Fig. S22 Co-expression of the DEGs in six tissues. Altogether there are14 clusters containing different numbers of DEGs. Cluster 6, 7, 9 and 11 related to flavonoid biosynthesis were extracted and depicted on Fig. 7.


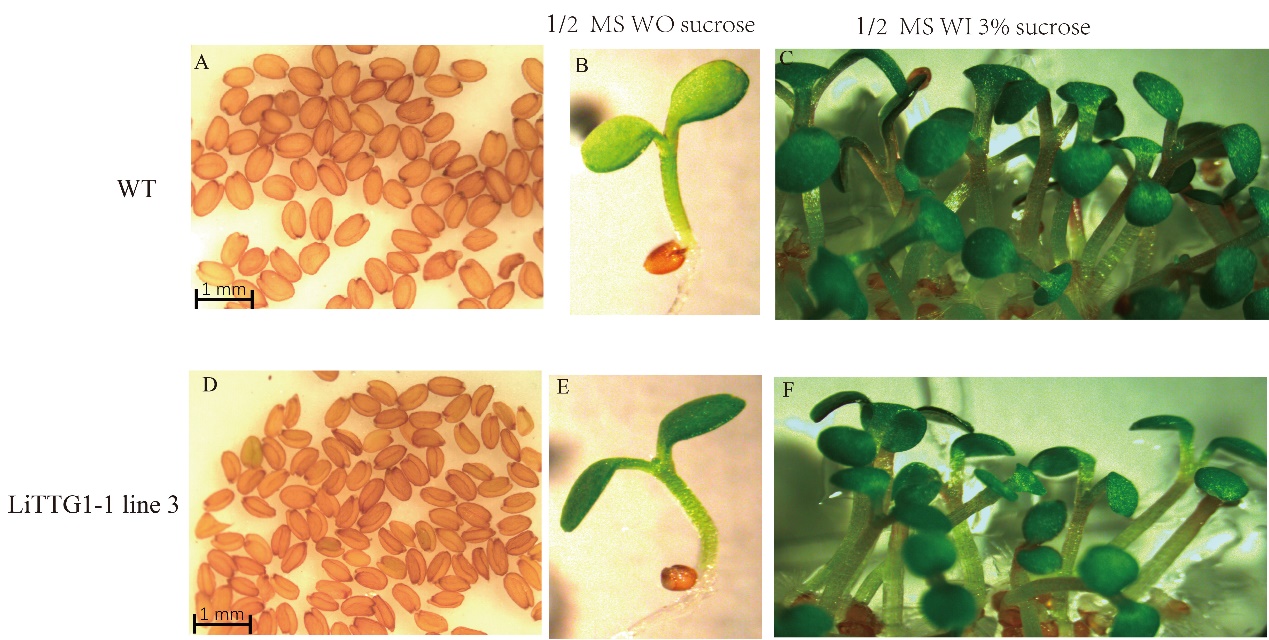


Supplementary Fig. S23 Phenotype of Arabidopsis over expression LiTTG1. A-C, wild type (Clo-0); D-F, LiTTG1 over expression line 3 (same phenotype of other OE lines). B and E, seeds growth under 1/2 MS salt medium without (WO) sucrose. C and F, seeds growth under 1/2 MS salt medium with (WI) 3% sucrose. Phenotype of seeds and young seedling were observed by Leica S8AP0 dissecting microscope and photos were taken by Leica DFC 295 CDD through Leica QWin V3 software.


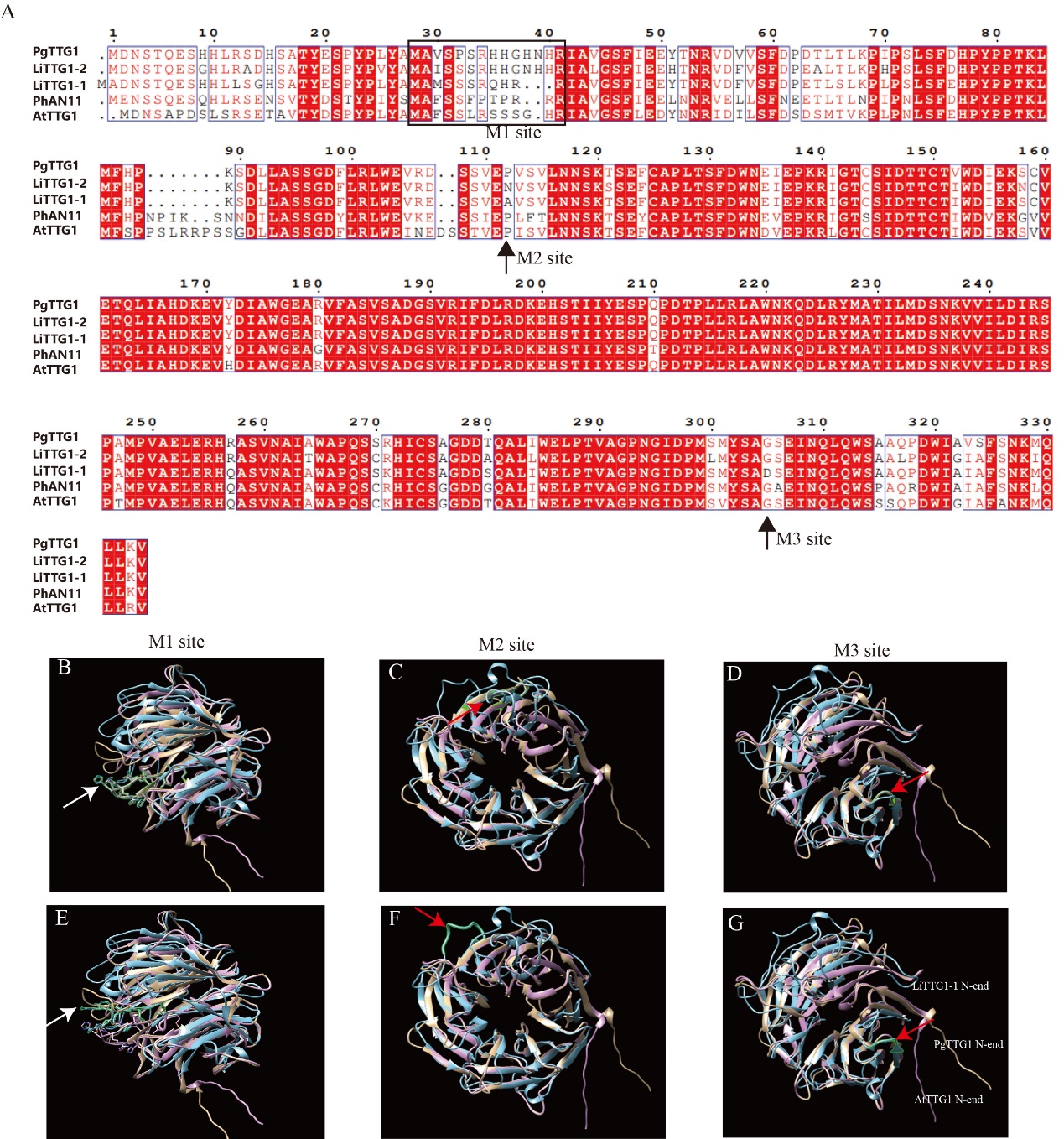


Supplementary Fig. S24 Multiple alignment and 3D structure of plant TTG1 proteins. (A) Multi-alignment of LiTTG1 (*L. indica*), PgTTG1 (*Punica granatum*, accession number HQ199314), PhAN11 (*Petunia hybrid*, accession number U94748), AtTTG1 (*Arabidopsis thaliana*, accession number AJ133743). B-G Merged 3D structure of AtTTG1, PgTTG1 and AtTTG1. (B-D) Structure variation at the M1, M2 and M3 sites in PgTTG1; (E-G) Structure variation in LiTTG1-1. PgTTG1, LiPPG1 and AtTTG1 are indicated by gold, cyan and pink, respectively.
