# Supplementary material for: Genomic and transcriptomic studies on flavonoid biosynthesis in Lagerstroemia indica
Source: BMC Plant Biol. 2024 Mar 5;24:171. doi: 10.1186/s12870-024-04776-4 (PMC10913235; doi:10.1186/s12870-024-04776-4)
Supplement: Supplementary file 2 — Supplementary Material 2: Supplementary tables [file 12870_2024_4776_MOESM2_ESM.docx]

Table S1 Overview of Illumina and PacBio reads

| Libraries | Insert size | Total data (G) | Read length (bp) | Sequence coverage (X) |
| --- | --- | --- | --- | --- |
| Illumina reads | 350 bp | 41.06 | 150 | 125.79 |
| PacBio reads | - | 206.28 | - | 631.93 |
| Total | - | 247.34 | - | 757.72 |

Table S2 Coverage of the reads

|  |  | Percentage (%) |
| --- | --- | --- |
| Reads | Mapping rate | 95.42 (%) |
| Genome | Average sequencing depth | 98.61 (%) |
|  | Coverage | 99.72 (%) |
|  | Coverage at least 4X | 99.69 (%) |
|  | Coverage at least 10X | 99.66 (%) |
|  | Coverage at least 20X | 99.61 (%) |

Table S3 BUSCO (Benchmarking Universal Single-Copy Orthologs: <http://busco.ezlab.org/>) assessment results of *L. indica* genome

|  | Percentage (%) |
| --- | --- |
| Complete BUSCOs | 98.1 |
| Complete and single-copy BUSCOs | 91.8 |
| Complete Duplicated BUSCOs | 6.3 |
| Fragmented BUSCOs | 0.7 |
| Missing BUSCOs | 1.2 |
| Total BUSCO groups searched | 1614 |

Table S4 Evaluation results of the assembly of *L. indica* by CEGMAG

|  | Number of assemble core gene | Completeness (%) |
| --- | --- | --- |
| complete | 233 | 93.95 |
| Complete + Partial | 242 | 97.58 |

* Total number of the core gene of six Eukaryotic model organisms is 248.

Table S5 The prediction of protein coding genes in *L. indica* genome

| Methods | Gene set | Number | Average transcript length(bp) | Average CDS length(bp) | Average exons per gene | Average exon length(bp) | Average intron length(bp) |
| --- | --- | --- | --- | --- | --- | --- | --- |
| De novo | Augustus | 29,205 | 2,518.51 | 1,152.82 | 4.83 | 238.47 | 356.19 |
|  | GlimmerHMM | 55,994 | 4,310.59 | 704.72 | 3.01 | 233.85 | 1,790.75 |
|  | SNAP | 35,683 | 2,478.31 | 835.58 | 3.6 | 232.09 | 631.74 |
|  | Geneid | 47,408 | 3,610.18 | 854.05 | 4.15 | 205.87 | 875.4 |
|  | Genscan | 30,156 | 7,294.27 | 1,388.23 | 6.88 | 201.76 | 1,004.34 |
| Homolog | Ptr | 24,153 | 2,422.88 | 1,129.66 | 4.85 | 232.87 | 335.8 |
|  | Egr | 23,653 | 2,541.40 | 1,168.77 | 5.06 | 230.92 | 337.97 |
|  | Vvi | 23,332 | 2,535.26 | 1,129.78 | 4.95 | 228.2 | 355.74 |
|  | Ath | 22,852 | 2,394.93 | 1,111.33 | 4.81 | 230.89 | 336.61 |
|  | Pgr | 25,542 | 2,587.36 | 1,189.51 | 5.14 | 231.39 | 337.58 |
| RNAseq | PASA | 121,662 | 3,056.36 | 1,068.95 | 5.39 | 198.48 | 453.17 |
|  | Transcripts | 50,401 | 6,807.13 | 2,188.11 | 6.82 | 320.92 | 793.9 |
| EVM | | 32,107 | 2,723.26 | 1,125.27 | 4.92 | 228.49 | 407.15 |
| Pasa-update* | | 31,807 | 2,740.92 | 1,148.33 | 4.96 | 231.42 | 401.95 |
| Final set* | | 28,812 | 2,912.85 | 1,213.74 | 5.27 | 230.29 | 397.86 |

Note: Included the un-translated-regions of genes.

Table S6 Gene structure of *L. indica* and other five kinds of plant

| Plant taxonomy | Species | Number | Average transcript length(bp) | Average CDS length(bp) | Average exons per gene | Average exon length(bp) | Average intron length(bp) |
| --- | --- | --- | --- | --- | --- | --- | --- |
| *Myrtales* (*Lythraceae*) | *L. indica* | 28,812 | 2,912.85 | 1,213.74 | 5.27 | 230.29 | 397.86 |
|  | *Punica granatum* | 29,229 | 2,789.38 | 1,342.30 | 5.48 | 245.16 | 323.36 |
| *Myrtales* (*Myrtaceae*) | *Eucalyptus grandis* | 35,931 | 4,067.16 | 1,307.81 | 4.93 | 265.17 | 701.78 |
| *Rhamnales* (*Vitaceae*) | *Vitis vinifera* | 29,927 | 4,728.63 | 1,095.81 | 4.75 | 230.72 | 968.88 |
| *Brassicales* (*Brassicaceae*) | *Arabidopsis thiliana* | 27,628 | 1,867.98 | 1,217.02 | 5.11 | 237.98 | 158.23 |
|  | *Populus trichocarpa* | 41,335 | 2,569.22 | 1,158.77 | 4.79 | 242.05 | 372.42 |

Table S7 Function prediction of protein genes in *L. indica* and other five kinds of plant

| Database | Gene number | Percentage (%) |
| --- | --- | --- |
| Swissprot | 23,140 | 80.31 |
| Nr | 27,494 | 95.43 |
| KEGG | 22,094 | 76.68 |
| InterPro | 27,636 | 95.92 |
| GO | 17,514 | 60.79 |
| Pfam | 22,606 | 78.46 |
| Annotated | 27,968 | 97.07 |
| Unannotated | 844 | 2.93 |
| Total | 28812* | - |

Redundant sequences were removed.

Table S8 The categories of non-coding RNA

| Type | | Copy number | Average length(bp) | Total length(bp) | % of genome |
| --- | --- | --- | --- | --- | --- |
| miRNA | | 327 | 140.97 | 46,097 | 0.014227 |
| tRNA | | 552 | 74.67 | 41,217 | 0.012721 |
| rRNA | rRNA | 214 | 208.47 | 44,612 | 0.013768 |
|  | 18S | 25 | 857.48 | 21,437 | 0.006616 |
|  | 28S | 47 | 138.15 | 6,493 | 0.002004 |
|  | 5.8S | 17 | 141.06 | 2,398 | 0.00074 |
|  | 5S | 125 | 114.27 | 14,284 | 0.004408 |
| snRNA | snRNA | 607 | 115.19 | 69,920 | 0.021579 |
|  | CD-box | 369 | 104.38 | 38,515 | 0.011887 |
|  | HACA-box | 67 | 127.33 | 8,531 | 0.002633 |
|  | splicing | 170 | 132.79 | 22,575 | 0.006967 |
|  | scaRNA | 1 | 299 | 299 | 0.00009 |

Table S9 Repeat sequence of the *L. indica* genome

| Type | Repeat Size(bp) | % of genome |
| --- | --- | --- |
| Tandem repeat fragment | 10,613,872 | 3.28 |
| Repeatmasker | 138,951,432 | 42.88 |
| Proteinmask | 14,436,302 | 4.46 |
| Total | 141,579,781 | 43.69 |

Table S10 Type of transposons elements of the *L. indica* genome

|  | Denovo+Repbase | | TE Proteins | | Combined TEs | |
| --- | --- | --- | --- | --- | --- | --- |
|  | Length(bp) | % in Genome | Length(bp) | % in Genome | Length(bp) | % in Genome |
| DNA | 5,892,812 | 1.82 | 181,137 | 0.06 | 5,932,052 | 1.83 |
| LINE | 3,404,049 | 1.05 | 503,611 | 0.16 | 3,618,816 | 1.12 |
| SINE | 2,592 | 0.00 | 0 | 0 | 2,592 | 0.00 |
| LTR | 103,260,275 | 31.87 | 13,751,743 | 4.24 | 103,933,033 | 32.08 |
| Unknown | 33,328,338 | 10.29 | 0 | 0 | 33,328,338 | 10.29 |
| Total | 138,951,432 | 42.88 | 14,436,302 | 4.46 | 139,471,565 | 43.04 |

LINE: long interspersed nuclear element

SINE: short interspersed nuclear elements

LTR: long terminal retrotransposon

Table S11 The orthologous gene number of *Lagerstroemia india* and other 13 plants

| Species | Single-copy genes | Multiple-copy genes | Unique | Other | Uncluster | Total gene number |
| --- | --- | --- | --- | --- | --- | --- |
| *Ginkgo biloba* | 3602 | 5701 | 7271 | 5990 | 9218 | 31782 |
| *Cerasus serrulata* | 3477 | 5969 | 2087 | 13914 | 3647 | 29094 |
| *Prunus mume* | 3741 | 4997 | 229 | 12760 | 1401 | 23128 |
| *Pyrus bretschneideri* | 1799 | 12264 | 1116 | 16726 | 2738 | 34643 |
| *Rosa chinensis* | 3594 | 6200 | 3365 | 13603 | 11891 | 38653 |
| *Acer truncatum* | 3529 | 5239 | 2008 | 12352 | 5280 | 28408 |
| *Arabidopsis thiliana* | 3233 | 6507 | 2949 | 10998 | 3619 | 27306 |
| *Populus trichocarpa* | 1874 | 11346 | 2807 | 17483 | 7724 | 41234 |
| *Punica granatum_L.* | 3622 | 5266 | 604 | 12458 | 1693 | 23643 |
| *Lagerstroemia indica* | 2726 | 8252 | 466 | 12195 | 5172 | 28811 |
| *Eucalyptus grandis* | 3463 | 7562 | 3933 | 16842 | 3932 | 35732 |
| *Vitis vinifera* | 3639 | 5503 | 1972 | 11223 | 6030 | 28367 |
| *Oryza sativa* | 3472 | 5656 | 6256 | 8523 | 11452 | 35359 |

Table S12 Composition of the Anthocyanidins/Flavonoid

| anthocyanidin/Flavonoid | Content (μg /g dry weight) | | |
| --- | --- | --- | --- |
|  | Mea SD | Mean ± SD | Mean ± SD |
| Cya-3,5-O-DiGlu | 0.027784 ± 0.000615 | 1.6225 ± 0.094284 | 0.640976 ±0.038294 |
| Cya-3-(6-O-p-Caf)-Glu | nd | nd | 0.024732 ± 0.002941 |
| Cya-3-O-gal | nd | 4.102908 ± 0.1888 | 2.750701 ± 0.288025 |
| Cya-3-O-Xyl | 0.007762 ± 0.001288 | 0.312392 ± 0.013449 | 0.245245 ± 0.00615 |
| Cya-3-O-Soph | nd | 0.025974 ± 0.000183 | 0.013952 ± 0.000728 |
| Cya-3-O-Rut | 0.12513±0.010168 | 0.09883 ± 0.00286 | 0.173487 ± 0.008199 |
| Cya-3-O-(6-O-p-Cou)-Glu | 0.532452 ± 0.053824 | 0.709374 ± 0.053458 | 0.22957 ± 0.013102 |
| Cya-3-O-Glu | 5.456305 ± 0.187121 | 232.4581 ± 3.761664 | 154.9189 ± 2.111887 |
| Del-3-O-Rut-5-O-Glu | nd | 0.014796 ± 0.000373 | 0.013067 ± 0.001555 |
| Del-3-O-Ara | nd | 0.153305 ± 0.010007 | 0.31776 ± 0.004854 |
| Del-3,5-O-DiGlu | nd | 2.437065 ± 0.153035 | 0.899362 ± 0.05073 |
| Del-3-O-rhamnoside | 0.022311 ± 0.001728 | 0.167771 ± 0.004102 | 0.173742 ± 0.002449 |
| Del-3-O-(6-O-p-Cou)-Glu | 0.036171 ± 0.004075 | 0.153355 ± 0.00979 | 0.392835 ± 0.002386 |
| Del-3-O-Rut | 0.011809 ± 0.001866 | 0.014684 ± 0.002396 | 0.020036 ± 0.00079 |
| Del-3-O-Glu | 67.03091 ± 0.759011 | 2262.489 ± 42.27914 | 2191.505 ± 73.75705 |
| Mal-3-O-(6-O-p-Cou)-Glu | 0.659661± 0.067314 | 1.301875 ± 0.066844 | 0.502699 ± 0.019109 |
| Mal-3-O-Ara | 0.020881±0.001506 | 0.550453 ± 0.0413 | 0.382272 ± 0.007145 |
| Mal-3,5-O-DiGlu | 7.18959±0.418048 | 114.3534 ± 4.143953 | 42.18818 ± 0.302002 |
| Mal-3-O-Rut | 0.097537±0.000487 | 0.057237 ± 0.004634 | 0.073495 ± 0.002862 |
| Mal-3-O-Glu | 343.9832±8.763285 | 1819.597 ± 43.26411 | 1059.881 ± 33.08293 |
| Mal-3-O-(6-O-malonyl-beta-D-Glu) | nd | 0.016897 ± 0.000826 | 0.017058 ± 0.000763 |
| Pel-3-O-galactoside | nd | 0.063057 ± 0.004236 | nd |
| Pel-3,5-O-DiGlu | nd | 0.011685 ± 0.000972 | 0.006216 ± 0.000733 |
| Pel-3-O-Glu | 0.052404±0.003444 | 0.715122 ± 0.011421 | 0.314491 ± 0.002615 |
| Peo-3-O-Rut | 0.16695±0.00455 | 0.040138 ± 0.006066 | 0.063555 ± 0.007488 |
| Peo-3-O-Sam-5-O-Glu | nd | 0.020455 ± 0.001819 | nd |
| Peo-3-O-Ara | nd | 0.001753 ± 0.000427 | nd |
| Peo-3-O-Sam | 0.059759 ± 0.009588 | 0.048535 ± 0.001722 | 0.045032 ± 0.003215 |
| Peo-3-O-Glu | 3.38442 ± 0.177159 | 25.15653 ± 0.528854 | 12.08537 ± 0.260271 |
| Peo-3-O-(6-O-p-Cou)-Glu | 0.083161 ± 0.01214 | 0.026558 ± 0.002013 | 0.007781 ± 0.000226 |
| Peo-3,5-O-DiGlu | 0.241618 ± 0.00611 | 5.973802 ± 0.165979 | 1.166286 ± 0.044432 |
| Pet-3-O-galactoside | 10.96814 ± 0.701126 | 197.7115 ± 3.830596 | 156.0521 ± 5.693519 |
| Pet-3-O-Ara | 0.034963 ± 0.004089 | 1.983133 ± 0.04775 | 1.90131 ± 0.058081 |
| Pet-3-O-Glu | 94.91469 ± 3.316585 | 1623.73 ± 28.00094 | 1364.056 ± 26.81074 |
| PA B1 | 0.030935 ± 0.000958 | 0.01262 ± 0.002069 | 0.010308 ± 0.001083 |
| PA B3 | 0.018902 ± 0.001741 | 0.020261 ± 0.001846 | 0.015519 ± 0.001141 |
| Dihydrokaempferol | 0.013787 ± 0.000641 | 0.273118 ± 0.014233 | 0.022358 ± 0.001185 |
| Dihydromyricetin | 22.89846 ± 0.36039 | 42.08253 ± 1.272595 | 41.9302 ± 0.922844 |
| Naringenin | 0.232768 ± 0.006787 | 1.363847 ± 0.052426 | 0.199188 ± 0.017878 |
| Naringenin-7-O-Glu | 0.467229 ± 0.042164 | 0.894379 ± 0.026251 | 0.207493 ± 0.029433 |
| Quercetin-3-O-Glu | 8.244496 ± 0.174055 | 34.24097 ± 2.860838 | 14.91071 ± 0.747855 |
| Kaempferol-3-O-Rut | 3.603312 ± 0.162056 | 1.458911 ± 0.037669 | 3.040185 ± 0.168075 |
| Rutin | 6.002233 ± 0.573115 | 21.23644 ± 1.684862 | 16.45102 ± 0.837406 |
| Afzelin | 0.288205 ± 0.026059 | 0.177408 ± 0.002009 | 0.577704 ± 0.02209 |

Cya, Cyanidin; DiGlu, diglucoside; Gal, galactoside; p-caff, p-caffeoyl; Xyl, xyloside; Soph, sophoroside; Rut, rutinoside; Glu,glucoside; Del, Delphinidin; Ara, arabinoside; p-Cou, p-coumaroyl; Mal, Malvidin; Pel, Pelargonidin; Peo, Peonidin; Sam, sambubioside; Pet, Petunidin; PA, Procyanidin.

Table S13 PAL and anthocyanin pathway genes in L. indica genome

| Pathway | Gene/enzyme function | L. indica | *Punica granatum* | *E. grandis*, |
| --- | --- | --- | --- | --- |
| PAL | phenylalanine ammonialyase (PAL) | evm.model.Chr1.988_ LiPAL1  evm.model.Chr2.986_ LiPAL2  evm.model.Chr2.1238_ LiPAL3  evm.model.Chr3.626_ LiPAL4  evm.model.Chr7.953_ LiPAL5  evm.model.Chr18.68 LiPAL6 | rna-XM_031525122.1  rna-XM_031526023.1  rna-XM_031516628.1 | Eucgr.A01144.1_EgrPAL1  Eucgr.C03570.1_EgrPAL2  Eucgr.G02848.1_EgrPAL3  Eucgr.G02849.1_EgrPAL4  Eucgr.G02850.1_EgrPAL5  Eucgr.G02851.1_EgrPAL6  Eucgr.G02852.1_EgrPAL7  Eucgr.J00907.1_EgrPAL8  Eucgr.J01079.1_EgrPAL9 |
|  | Cinnamate 4-hydroxylase (C4H) | evm.model.Chr2.346_LiC4H1  evm.model.Chr8.2_LiC4H2  evm.model.Chr15.1216 _LiC4H3 | rna-XM_031529725.1  rna-XM_031529726.1  rna-XM_031525063.1 | Eucgr.J01844.1_EgrC4H1  Eucgr.C00065.1_EgrC4H2 |
|  | 4-coumarate CoA ligase (4CL) | evm.model.Chr7.1630 Li4CL1  evm.model.Chr8.1392.2 Li4CL2  evm.model.Chr15.23 Li4CL3 | rna-XM 031530258.1  rna-XM 031526422.1  rna-XM 031526423.1 | Eucgr.C02284.1_Egr4CL1  Eucgr.K00087.2_Egr4CL2 |
| Flavonoid skeleton biosynthesis | Chalcone synthase (CHS) | evm.model.Chr2.748_LiCHS1  L. indica newGene 1711_LiCHS2  L. indica newGene 4627_LiCHS3  L. indica newGene 1381_LiCHS-like 1  L. indica newGene 6485_LiCHS-like 2 | rna-XM 031525740.1  rna-XM 031526042.1 | Eucgr.I01579.1  Eucgr.H02828.1  Eucgr.H03914.1 |
|  | Chalcone isomerase (CHI) | evm.model.Chr7.744_LiCHI | rna-XM_031523868.1  (KF841616\|PgrCHI) | Eucgr.F03816.1 |
| LABG | Flavanone 3-hydroxylase (F3H) | evm.model.Chr24.886_LiF3H | rna-XM 031545642.1  rna-XM 031532291.1 | Eucgr.J02430.1  Eucgr.J02430.2 |
|  | Dihydroflavonol-4-reductase (DFR) | evm.model.Chr12.807.1_LiDFR | rna-XM 031551222.1  rna-XM 031549467.1  rna-XM 031549468.1 | Eucgr.F01589.1 |
|  | Leucoanthocyanidin oxygenase /anthocyanidin synthase (LDOX/ANS) | evm.model.Chr4.1404_ LiLODX/ANS | rna-XM 031548104.1 | Eucgr.D01945.1 |

Table S13 PAL and anthocyanin pathway genes in L. indica genome (continued)

| Pathway | Gene/enzyme function | L. indica | *Punica granatum* | *E. grandis*, |
| --- | --- | --- | --- | --- |
| B-ring Hydroxylation of flavonoid | Flavonoid 3'-hydroxylase (F3′H) | evm.model.Chr14.907_LiF3'H1  evm.model.Chr16.350_LiF3'H2  evm.model.Chr16.351_LiF3'H3 | rna-XM_031550730.1 | Eucgr.B00578.1  Eucgr.B00580.1  Eucgr.B00582.1 |
|  | Flavonoid 3'5'-hydroxylase (F3′5′H) | evm.model.Chr8.398_LiF3'5’H | rna-XM_031531118  rna-XM_031531119 | Eucgr.B03742.1  Eucgr.B03746.1  Eucgr.J00685.1 |
| Flavone biosynthesis | flavone synthase I (FNSI) | evm.model.Chr2.1730_LiFNSI1  evm.model.Chr4.1518_LiFNSI2  evm.model.Chr6.1345_LiFNSI3 | rna-XM 031526469.1  rna-XM 031526470.1  rna-XM 031536133.1  rna-XM 031526857.1 | Eucgr.K00448.1  Eucgr.K00039.1  Eucgr.K00447.1  Eucgr.K00040.1 |
|  | flavone synthase II (FNSII) | evm.model.Chr8.72 LiFNSII1  evm.model.Chr11.99 LiFNSII2  evm.model.Chr11.101 LiFNSII3 | rna-XM 031533622.1  rna-XM 031547999.1  rna-XM 031519411.1  rna-XM 031542802.1  rna-XM 031539802.1 | Eucgr.B02410.1  Eucgr.B02408.1  Eucgr.I01315.1  Eucgr.A02424.1  Eucgr.A02423.1 |
| Flavonol biosynthesis | Flavonol synthase(FLS) | evm.model.Chr6.328_evm.model.Chr6.329_LiFLS1  evm.model.Chr6.332_LiFLS2  evm.model.Chr17.1598_LiFLS3 | rna-XM 031529124.1  rna-XM 031537792.1  rna-XM 031548104.1  rna-XM 031534685.1 | Eucgr.L00738.1  Eucgr.E01689.1  Eucgr.F03761.1  Eucgr.F03763.1 |
| Flavanol  (Catechin and Epicatechin) | Leucoanthocyanidin reductase (LAR) | evm.model.Chr11.119_LiLAR1  evm.model.Chr15.1103.10_LiLAR2 | rna-XM 031533569.1  rna-XM 031545073.1 | Eucgr.B03055.1  Eucgr.K02656.1  Eucgr.K02657.1 |
|  | Anthocyanidin reductase (ANR) | evm.model.Chr20.974_LiANR | rna-XM 031542370.1  rna-XM 031542369.1 | Eucgr.D00689.1  Eucgr.D00700.1  Eucgr.D00858.1 |

Table S14 Genes related to the flavonoid modification in *L. indica* genome

| Pathway | Gene/enzyme function | Gene ID and name |
| --- | --- | --- |
| Glycoside | UDP-glucose: Flavonol O- glucosyltransferases (UFOGT) | evm.model.Chr21.359_LiUFOGT1*  evm.model.Chr3.52_LiUFOGT2  evm.model.Chr4.1110_LiUFOGT3  evm.model.Chr6.1399_LiUFOGT4  evm.model.Chr9.291_LiUFOGT5  evm.model.Chr11.1053_LiUFOGT6  evm.model.Chr11.812.1_LiUFOGT7  evm.model.Chr16.367_LiUFOGT8  evm.model.Chr16.368_LiUFOGT9  evm.model.Chr17.79_LiUFOGT10  evm.model.Chr19.1125_LiUFOGT11  evm.model.Chr19.81_LiUFOGT12  evm.model.Chr23.248_LiUFOGT13  evm.model.Chr24.820_LiUFOGT14 |
|  | UDP-glucose:Flavonol C-glucosyltransferases (UFCGT) | evm.model.Chr16.483_LiUFCGT1  evm.model.Chr24.19_LiUFCGT2 |
|  | UDP-glucose: anthocyanin -5-O glucosyltransferase  (UA5OGT) | evm.model.Chr12.570_LiUA5OGT1  evm.model.Chr16.574.1_LiUA5OGT2 |
|  | Anthocyanin 3' or 7-O –glucosyltransferase  (UA7OGT) | evm.model.Chr3.445_LiUA7OGT1  evm.model.Chr3.447_LiUA7OGT2  evm.model.Chr6.656_LiUA7OGT3  evm.model.Chr13.294_LiUA7OGT4  evm.model.Chr13.295_LiUA7OGT5  evm.model.Chr20.215_LiUA7OGT6  evm.model.Chr20.216_LiUA7OGT7 |
|  | Anthocyanidin 3-O-glucosyltransferase  (UA3OGT) | evm.model.Chr8.1329_LiUA3OGT1  evm.model.Chr8.1330_Li UA3OGT2  Lagerstroemia indica newGene 4281_LiUA3OGT3  Lagerstroemia indica newGene 4284_Li UA3OGT4 |
|  | glycoside hydrolase family 1 glucosyltransferase (GH1-GT) | evm.model.Chr3.844_LiGH1GT1  evm.model.Chr5.264_LiGH1GT2  evm.model.Chr5.798_LiGH1GT3  evm.model.Chr6.402_LiGH1GT4  evm.model.Chr6.406_LiGH1GT5  evm.model.Chr7.515_LiGH1GT6  evm.model.Chr8.368_LiGH1GT7  evm.model.Chr8.369_LiGH1GT8  evm.model.Chr12.560_LiGH1GT9  evm.model.Chr14.846_LiGH1GT10  evm.model.Chr15.899_LiGH1GT11  evm.model.Chr17.568_evm.model.Chr17.569_LiGH1GT12  evm.model.Chr18.620_LiGH1GT13  evm.model.Chr22.159_LiGH1GT14  evm.model.Chr22.160_LiGH1GT15  evm.model.Chr24.201.1_LiGH1GT16 |

Table S14 Genes related to the flavonoid modification in *L. indica* genome (continued)

| Pathway | Gene/enzyme function | Gene ID and name |
| --- | --- | --- |
| Methylation | S-adenosyl-L-methionine (SAM)- dependent O-methyltransferases (Class II) | evm.model.Chr1.878_LiOMT1  evm.model.Chr6.152_LiOMT2  evm.model.Chr6.831_LiOMT3  evm.model.Chr6.1243_LiOMT4  evm.model.Chr6.1244_LiOMT5  evm.model.Chr13.292_LiAOMT6  evm.model.Chr16.616_LiOMT7  evm.model.Chr17.482.4_LiOMT8  evm.model.Chr19.1101_LiOMT9  evm.model.Chr19.1102_LiOMT10 evm.model.Chr19.1103_LiOMT11  evm.model.Chr19.1104_LiOMT12  evm.model.Chr23.751_LiOMT13 |
|  | S-adenosyl-L-methionine (SAM)- dependent O-methyltransferases (Class I) | evm.model.Chr7.459_ evm.model.Chr7.464_LiAOMT1  evm.model.Chr15.169_LiAOMT2  evm.model.Chr22.246_LiAOMT3 |
|  |  | evm.model.Chr6.652_LiCCoAOMT1  evm.model.Chr19.935_LiACCoAOMT2 |

*The function of orthologs have been identified.

Table S15 R2R3 MYBs in *L. indica*

| ID | Name | ID | Name |
| --- | --- | --- | --- |
| evm.model.Chr1.18 | LiMYB1 | evm.model.Chr6.813 | LiMYB48 |
| evm.model.Chr1.107 | LiMYB2 | evm.model.Chr6.1069 | LiMYB49 |
| evm.model.Chr1.193 | LiMYB3 | evm.model.Chr6.1124 | LiMYB50 |
| evm.model.Chr1.489 | LiMYB4 | evm.model.Chr6.1255 | LiMYB51 |
| evm.model.Chr1.753 | LiMYB5 | evm.model.Chr6.1256 | LiMYB52 |
| evm.model.Chr1.793 | LiMYB6 | evm.model.Chr6.1258 | LiMYB53 |
| evm.model.Chr1.923 | LiMYB7 | evm.model.Chr6.1331 | LiMYB54 |
| evm.model.Chr2.39 | LiMYB8 | evm.model.Chr7.925 | LiMYB55 |
| evm.model.Chr2.274 | LiMYB9 | evm.model.Chr7.114 | LiMYB56 |
| evm.model.Chr2.617 | LiMYB10 | evm.model.Chr7.115 | LiMYB57 |
| evm.model.Chr2.1087.1 | LiMYB11 | evm.model.Chr7.1311 | LiMYB58 |
| evm.model.Chr2.1253 | LiMYB12 | evm.model.Chr7.1477 | LiMYB59 |
| evm.model.Chr2.1716 | LiMYB13 | evm.model.Chr7.1589 | LiMYB60 |
| evm.model.Chr2.1794 | LiMYB14 | evm.model.Chr8.251 | LiMYB61 |
| evm.model.Chr3.267 | LiMYB15 | evm.model.Chr8.386 | LiMYB62 |
| evm.model.Chr3.270 | LiMYB16 | evm.model.Chr8.805 | LiMYB63 |
| evm.model.Chr3.606 | LiMYB17 | evm.model.Chr8.905 | LiMYB64 |
| evm.model.Chr3.648 | LiMYB18 | evm.model.Chr8.1327 | LiMYB65 |
| evm.model.Chr3.756 | LiMYB19 | evm.model.Chr9.23 | LiMYB66 |
| evm.model.Chr3.1353 | LiMYB20 | evm.model.Chr9.640 | LiMYB67 |
| evm.model.Chr4.77 | LiMYB21 | evm.model.Chr9.94 | LiMYB68 |
| evm.model.Chr4.590 | LiMYB22 | evm.model.Chr10.117 | LiMYB69 |
| evm.model.Chr4.800_evm.model.Chr4.801 | LiMYB23 | evm.model.Chr10.214 | LiMYB70 |
| evm.model.Chr4.846 | LiMYB24 | evm.model.Chr10.450 | LiMYB71 |
| evm.model.Chr4.865 | LiMYB25 | evm.model.Chr10.451 | LiMYB72 |
| evm.model.Chr4.866 | LiMYB26 | evm.model.Chr10.461 | LiMYB73 |
| evm.model.Chr4.1224 | LiMYB27 | evm.model.Chr10.574 | LiMYB74 |
| evm.model.Chr4.1292 | LiMYB28 | evm.model.Chr11.594 | LiMYB75 |
| evm.model.Chr4.1495 | LiMYB29 | evm.model.Chr11.918 | LiMYB76 |
| evm.model.Chr5.471 | LiMYB30 | evm.model.Chr11.953.1 | LiMYB77 |
| evm.model.Chr5.506 | LiMYB31 | evm.model.Chr12.39 | LiMYB78 |
| evm.model.Chr5.591 | LiMYB32 | evm.model.Chr12.122 | LiMYB79 |
| evm.model.Chr5.846 | LiMYB33 | evm.model.Chr12.348 | LiMYB80 |
| evm.model.Chr5.847 | LiMYB34 | evm.model.Chr12.368 | LiMYB81 |
| evm.model.Chr5.852.1 | LiMYB35 | evm.model.Chr12.702 | LiMYB82 |
| evm.model.Chr5.933 | LiMYB36 | evm.model.Chr13.90 | LiMYB83 |
| evm.model.Chr5.934 | LiMYB37 | evm.model.Chr13.128_evm.model.Chr13.129 | LiMYB84 |
| evm.model.Chr5.955 | LiMYB38 | evm.model.Chr14.398 | LiMYB85 |
| evm.model.Chr5.969 | LiMYB39 | evm.model.Chr13.410 | LiMYB86 |
| evm.model.Chr5.1041 | LiMYB40 | evm.model.Chr14.696 | LiMYB87 |
| evm.model.Chr5.1145 | LiMYB41 | evm.model.Chr13.700 | LiMYB88 |
| evm.model.Chr5.1301.2 | LiMYB42 | evm.model.Chr13.725 | LiMYB89 |
| evm.model.Chr5.1643 | LiMYB43 | evm.model.Chr13.726 | LiMYB90 |
| evm.model.Chr6.108 | LiMYB44 | evm.model.Chr14.844 | LiMYB91 |
| evm.model.Chr6.397 | LiMYB45 | evm.model.Chr14.926 | LiMYB92 |
| evm.model.Chr6.405 | LiMYB46 | evm.model.Chr15.444 | LiMYB93 |
| evm.model.Chr6.555 | LiMYB47 | evm.model.Chr15.744 | LiMYB94 |

Table S15 R2R3 MYBs in *L. indica* (continued)

| ID | Name | ID | Name |
| --- | --- | --- | --- |
| evm.model.Chr15.894 | LiMYB95 | evm.model.Chr20.657 | LiMYB117 |
| evm.model.Chr16.316 | LiMYB96 | evm.model.Chr21.147.1 | LiMYB118 |
| evm.model.Chr16.434 | LiMYB97 | evm.model.Chr21.520 | LiMYB119 |
| evm.model.Chr16.729 | LiMYB98 | evm.model.Chr21.813 | LiMYB120 |
| evm.model.Chr16.738 | LiMYB99 | evm.model.Chr22.38_evm.model.Chr22.37 | LiMYB121 |
| evm.model.Chr16.979 | LiMYB100 | evm.model.Chr22.75 | LiMYB122 |
| evm.model.Chr16.1057 | LiMYB101 | evm.model.Chr22.217.1 | LiMYB123 |
| evm.model.Chr16.1081 | LiMYB102 | evm.model.Chr22.415 | LiMYB124 |
| evm.model.Chr17.153 | LiMYB103 | evm.model.Chr22.828 | LiMYB125 |
| evm.model.Chr17.538 | LiMYB104 | evm.model.Chr23.65 | LiMYB126 |
| evm.model.Chr17.1147 | LiMYB105 | evm.model.Chr23.410 | LiMYB127 |
| evm.model.Chr17.1243 | LiMYB106 | evm.model.Chr23.606* | LiMYB128 |
| evm.model.Chr17.1318.1 | LiMYB107 | evm.model.Chr23.792 | LiMYB129 |
| evm.model.Chr17.1565 | LiMYB108 | evm.model.Chr23.986 | LiMYB130 |
| evm.model.Chr18.295 | LiMYB109 | evm.model.Chr23.1029 | LiMYB131 |
| evm.model.Chr18.816 | LiMYB110 | evm.model.Chr23.1058 | LiMYB132 |
| evm.model.Chr19.324 | LiMYB111 | evm.model.Chr24.20 | LiMYB133 |
| evm.model.Chr19.1156 | LiMYB112 | evm.model.Chr24.482* | LiMYB134 |
| evm.model.Chr19.1211 | LiMYB113 | evm.model.Chr24.497 | LiMYB135 |
| evm.model.Chr19.1284 | LiMYB114 | evm.model.Chr24.611.1 | LiMYB136 |
| evm.model.Chr19.1419 | LiMYB115 | evm.model.Chr24.980 | LiMYB137 |
| evm.model.Chr20.112 | LiMYB116 |  |  |

*Two genes not included in any of the subgroups.

Table S16 Comparing R2R3 MYB numbers among *Eucalyptus grandis*, *Lagerstroemia indica* L and Arabidopsis

| Subgroup (S) | *L. indica* | *Eucalyptus grandis* | *Arabidopsis thiliana* | *Subgroup (S)* | *L. indica* | *Eucalyptus grandis* | *Arabidopsis thiliana* |
| --- | --- | --- | --- | --- | --- | --- | --- |
| S1 | 9↑ | 4 | 5 | S23 | 3 | 2 | 3 |
| S2 & S3 +AtM10 & AtM72 | 8↑ | 4 | 7 | S25 + AtM98 | 4↓ | 2 | 7 |
| S4 + AtM6 & AtM8 | 6 | 4 | 6 | SAtM5 | 2 | 2 | 1 |
| S5 | 8↑ | 16 | 1 | SAtM27, AtM48 & AtM59 | 2 | 1 | 3 |
| S6 | 2↓ | 11 | 4 | SAtM46 & AtM83 | 4↑ | 2 | 2 |
| S7 | 2 | 1 | 3 | SAtM47 & AtM95 | 0 | 0 | 2 |
| S9 | 4 | 3 | 3 | SAtM71, AtM79 & AtM121 | 2 | 1 | 3 |
| S10 & S24 | 4 | 5 | 6 | SAtM88 & AtM124 | 0↓ | 2 | 2 |
| S11 + AtM49 | 4 | 5 | 4 | SAtM35 | 0↓ | 1 | 1 |
| S12 | 0 | 0 | 6 | SAtM80 | 0↓ | 1 | 1 |
| S13 | 13↑ | 7 | 4 | SAtM82 | 0↓ | 1 | 1 |
| AtM26 + AtM67+ AtM103 | 0 | 0 | 3 | SAtM20, AtM40, AtM42, AtM43, AtM85 & AtM99 | 5 | 6 | 6 |
| S14 | 9↑ | 10 | 6 | SAtM91 | 1 | 1 | 1 |
| S15 | 2 | 4 | 3 | SAtM125 | 0↓ | 1 | 1 |
| S16 | 1 | 2 | 3 | WPS I | 4 | 6 | 0 |
| S18 | 2↓ | 2 | 7 | WPS II | 2 | 3 | 0 |
| S19 + AtM57 | 0↓ | 2 | 3 | WPS III | 4 | 4 | 0 |
| S20 | 10↑ | 4 | 6 | WPS IV | 0↓ | 6 | 0 |
| S21 + AtM89 | 11↑ | 6 | 8 | WPS V | 2 | 2 | 0 |
| S22 | 7↑ | 5 | 4 | *L. indica s*pecific | 2↑ | 0 | 0 |

Table S17 LiMYBs co-expressed to the flavonoid biosynthesis pathway

| Number | Gene Name | Cluster | Subgroup |
| --- | --- | --- | --- |
| 1 | LiMYB12 | cluster6 | S14 |
| 2 | LiMYB15 | cluster6 | S21 + AtM89 |
| 3 | LiMYB24 | cluster6 | S2 & S3 + SAtM10 & SAtM72 |
| 4 | LiMYB41 | cluster6 | S22 |
| 5 | LiMYB42 | cluster6 | S2 & S3 + SAtM10 & SAtM72 |
| 6 | LiMYB36 | cluster6 | S2 & S3 + SAtM10 & SAtM72 |
| 7 | LiMYB37 | cluster6 | S2 & S3 + SAtM10 & SAtM72 |
| 8 | LiMYB49 | cluster6 | S11 + AtM49 |
| 9 | LiMYB58 | cluster6 | S21 + AtM89 |
| 10 | LiMYB72 | cluster6 | S6 |
| 11 | LiMYB86 | cluster6 | S10 & S24 |
| 12 | LiMYB90 | cluster6 | S2 & S3 + SAtM10 & SAtM72 |
| 13 | LiMYB99 | cluster6 | S2 & S3 + SAtM10 & SAtM72 |
| 14 | LiMYB107 | cluster6 | WIII |
| 15 | LiMYB117 | cluster6 | S20 |
| 16 | LiMYB8 | cluster7 | S2 & S3 + SAtM10 & SAtM72 |
| 17 | LiMYB29 | cluster7 | WII |
| 18 | LiMYB92 | cluster7 | S1 |
| 19 | LiMYB118 | cluster7 | S11 + AtM49 |
| 20 | LiMYB119 | cluster7 | S20 |
| 21 | LiMYB125 | cluster7 | WI |
| 22 | LiMYB9 | cluster9 | S4 + AtM6 & AtM8 |
| 23 | LiMYB75 | cluster9 | S20 |
| 24 | LiMYB81 | cluster9 | S13 |
| 25 | LiMYB26 | cluster11 | WI |
| 26 | LiMYB46 | cluster11 | WIII |
| 27 | LiMYB59 | cluster11 | S13 |
| 28 | LiMYB69 | cluster11 | S1 |

Table S18 Primers used in this study

| Primer name | | Sequence (5′ to 3′) | Usage |
| --- | --- | --- | --- |
| *LiTTG1 F* | GCAGAGCGGAAATGGACAA | Cloning cDNA of LiTTG1 |  |
| *LITTG1 R* | GAATCAACATTAATAGGATGAGCTG |  |  |
| *pWM101-TTG1-F* | TCGAGCTTTCGCGAGCTCGGTACCATGGACAACTCGACTCAGGAG | Construction plant expression vector |  |
| *pWM101-TTG1-R* | GCATGCCTGCAGGTCGACTCTAGATCAAACTTTCAAGAGCTGCATTTTGTT |  |  |
| *NbActin-F* | AATGATCGGAATGGAAGCTG | Internal control qPCR |  |
| *NtActin-R* | TGGTACCACCACTGAGGACA |  |  |
| *NtDFR-F* | GCTGTTCATACCTCTTCTCCTC |  |  |
| *NtDFR-R* | GCTTGACCTTCTTGTTGTTCTC |  |  |
| *NtANS-F* | TGGCGTTGAAGCTCATACTG |  |  |
| *NtANS-R* | GGAATTAGGCACACACTTTGC |  |  |
| *NtUFGT-F* | GAGTGCATTGGATGCCTTTT |  |  |
| *NtUFGT-R* | CCAGCTCCATTAGGTCCTTG |  |  |
